# Trans‐Synaptic Virus Tracing Reveals Reciprocal Connections of the Interpeduncular Nucleus With the Mood, Exteroceptive, Interoceptive, and Motor Control Systems

**DOI:** 10.1002/cne.70105

**Published:** 2025-11-14

**Authors:** Audrey K. Wong, Bor‐Wei Cherng, Hisaya Kakinuma, Claire Wyart, Hitoshi Okamoto

**Affiliations:** ^1^ RIKEN Center for Brain Science Wako Saitama Japan; ^2^ Sorbonne University, Institut du Cerveau, INSERM U1127 Paris France; ^3^ Center for Advanced Biomedical Sciences, Faculty of Science and Engineering Waseda University Shinjuku Tokyo Japan; ^4^ Institute of Neuropsychiatry Shinjuku Tokyo Japan

**Keywords:** griseum centrale, habenula, interoception, interpeduncular nucleus (IPN), periaqueductal gray (PAG), stress resilience, trans‐synaptic viral tracing

## Abstract

The interpeduncular nucleus (IPN) is an evolutionarily conserved brain nucleus that receives direct input from the habenula (Hb). In zebrafish (*Danio rerio*), it is involved in decision‐making dependent on the idiothetic perception of the status of the body, such as head direction and posture. It is also known that the potentiation of the Hb–IPN–griseum centrale (GC) circuit makes fish resilient to stress in coping with fear and social conflict. To address why the same neural circuit controls these two distinctive physiological aspects, we performed anterograde and retrograde trans‐synaptic viral tracing, retrograde mono‐synaptic viral tracing, and lipophilic dye tracing to map the connectivity from the dorsal and intermediate IPN. We revealed reciprocal connections of d/iIPN and GC neurons with multiple brain regions for sensory inputs, including interoceptive systems that receive proprioception and the perception of balance and water flow, autonomic nervous systems for visceral control, and exteroceptive systems that receive vision and olfaction through the Hb. The positively labeled signals also cover the emotional regulatory system (i.e., serotonin, noradrenaline, and dopamine neurons) and the motor control system that administers the presentation of behaviors. Our anatomical results imply that multimodal sensorimotor information may converge in the Hb–IPN–GC circuit, hinting at its possible involvement in integrating inner states for responses to upcoming external challenges and in regulating allostasis through anticipatory biological reactions.

## Graphical abstract

The interpeduncular nucleus (IPN) in zebrafish plays a pivotal role in directional decision‐making, social conflict, and fear responses. The anterograde and retrograde connections of the dorsal and intermediate IPN are revealed through the use of monosynaptic and trans‐synaptic viral tracers.

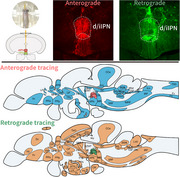

**Abbreviations**
A: anterior thalamic nucleus
aGC: anterior griseum centrale
ALLN: anterior lateral line nerve
CB: cerebellum
CCe: corpus cerebelli
CP: central posterior thalamic nucleus
dHb: dorsal habenula
dIPN: dorsal interpeduncular nucleus
DiV: diencephalic ventricle
DON: descending octaval nucleus
Dp: posterior zone of the dorsal pallium
EG: granular eminence
Flv: funiculus lateralis pars ventralis
ENd: entopeduncular nucleus
FR: fasciculus retroflexus
Fv: funiculus ventralis
GC: griseum centrale
Hb: habenula
HbM: medial habenula
Hc: caudal zone of the periventricular hypothalamus
HT: hypothalamus
iIPN: intermediate interpeduncular nucleus
IMRF: intermediate reticular formation
IPN: interpeduncular nucleus
IR: inferior raphe
IRF: inferior reticular formation
LC: locus coeruleus
LFB: lateral forebrain bundle
LH: lateral hypothalamic nucleus
LLF: lateral longitudinal fascicle
LR: lateral recess of the diencephalic ventricle
LX: lobus vagus
MFB: medial forebrain bundle
MLF: medial longitudinal fascicle
MNV: mesencephalic nucleus of trigeminal nerve
MO: medulla oblongata
MON: medial octavolateralis nucleus
MR: median raphe
MS: medulla spinalis
NIII: oculomotor nucleus
NLV: nucleus lateralis valvulae
OB: olfactory bulbs
ON: optic nerve
OT: optical tectum
P: posterior thalamic nucleus
pGC: posterior griseum centrale
PGZ: periventricular gray zone of optic tectum
PPa: parvocellular preoptic nucleus, anterior part
PPp: parvocellular preoptic nucleus, posterior part
PR: posterior recess of DiV
PTN: posterior tuberal nucleus
RV: rhombencephalic ventricle
SO: secondary octaval population (of McCormick and Hernandez)
SRF: superior reticular formation
SZMT: subventricular zone of mesencephalic tegmentum
TBS: bulbospinal tract
Tel: telencephalon
TeO: tectum opticum
TeV: telencephalic ventricle
TPp: periventricular nucleus of posterior tuberculum
TS: torus semicircularis
TSc: central nucleus of torus semicircularis
TSvl: ventrolateral nucleus of torus semicircularis
TTB: tectobulbar tract
TTBc: crossed tectobulbar tract
TTBr: uncrossed tectobulbar tract
vHb: ventral habenula
VIIm: facial nerve motor nucleus
VIImr: facial motor root
VIIsr: facial sensory root
vIPN: ventral interpeduncular nucleus
VL: ventrolateral thalamic nucleus
VM: ventromedial thalamic nucleus
Vv: ventral nucleus of ventral telencephalic area
Xm: vagal nerve motor nucleus

## 1 Introduction

The interpeduncular nucleus (IPN) is an unpaired structure located at the ventral midline of the tegmentum in the midbrain that has been proposed to be a critical integrative center in the limbic system (Morley 1986; Okamoto et al. 2011, 2021; Lima et al. 2017; Quina et al. 2017). A major source of innervation to the IPN is the medial habenula (HbM), and together they form the habenula (Hb)–IPN pathway via the fasciculus retroflexus (FR) (Herkenham and Nauta 1979; Sutherland 1982; Aizawa et al. 2005; Gamse et al. 2005; Kim 2009). The Hb–IPN circuit is one of the most evolutionarily conserved components of the vertebrate brain (Amo et al. 2010; Aizawa et al. 2011, 2012; Okamoto et al. 2011) and has been associated with a broad range of behaviors, including addiction, mood regulation, modulation of fear response, social aggression, and directional decision‐making (Jesuthasan 2012; Chou et al. 2016; McLaughlin et al. 2017; Dragomir et al. 2019; Cherng et al. 2020; Nakajo et al. 2020; Petrucco et al. 2023).

**FIGURE 1:**
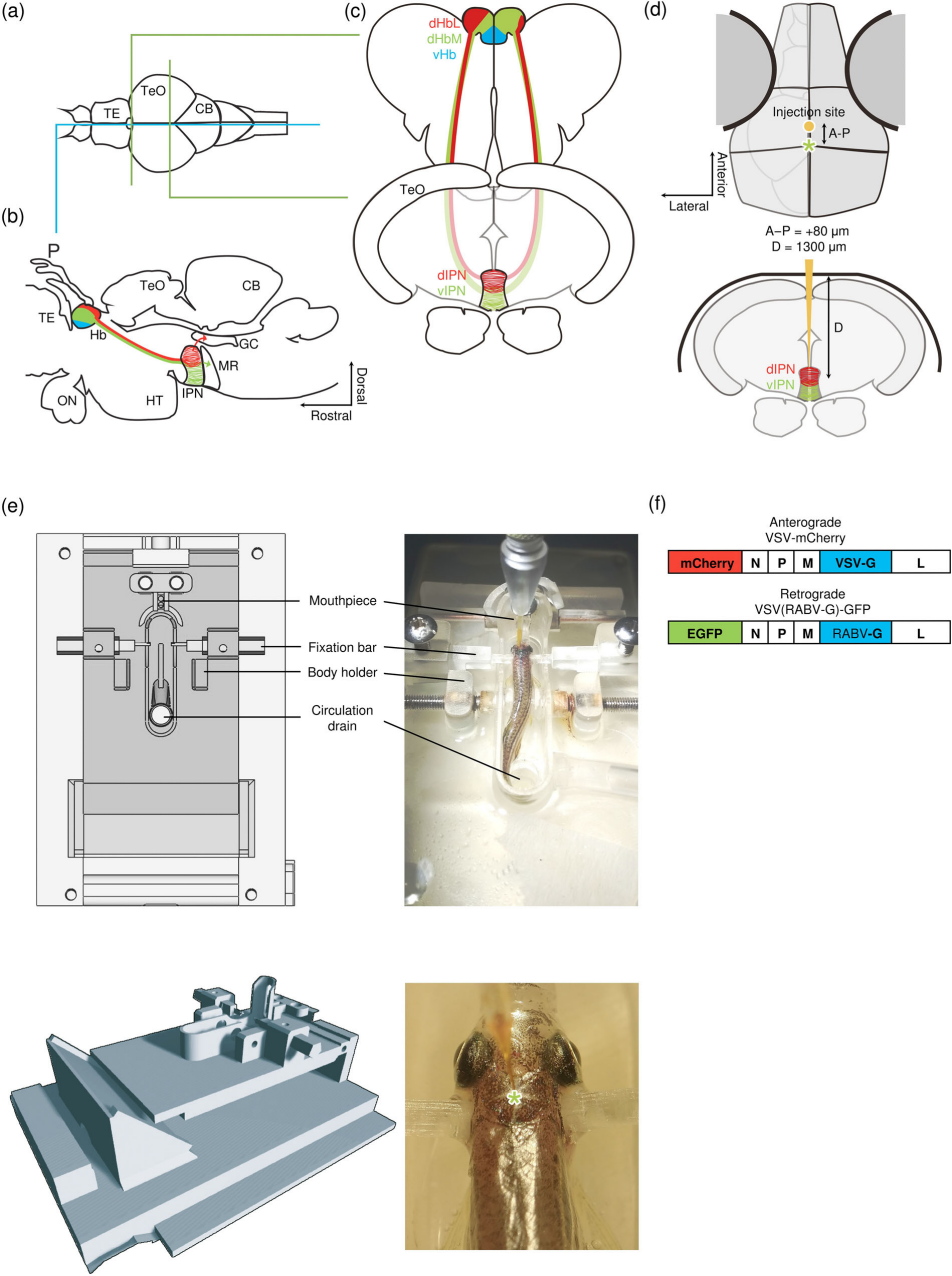
Schematic diagrams illustrating the methodology used to achieve in vivo tracing from the interpeduncular nucleus in the adult zebrafish. (a–c) Schematic diagram of the zebrafish habenula–interpeduncular nucleus circuit. (a) Dorsal view of adult zebrafish brain. The blue line indicates the level of the sagittal section in (b); the green lines indicate the level of the coronal section in (c). (b) Sagittal section of the adult zebrafish brain showing the projection of the lateral subregion of the dorsal habenula (dHbL) and the medial subregion of the dorsal habenula (dHbM) to the d/iIPN (red) and the i/vIPN (green). The ventral habenula (vHb) is shown in blue. The griseum centrale (GC) receives efferent input from d/iIPN (red arrow), whereas the dHbM‐i/vIPN pathway is connected to the median raphe (MR) (green arrow). (c) Coronal section of the adult zebrafish brain showing the connections of the habenula subnuclei with the IPN. (d) Stereotaxic coordinates used to locate the IPN for microinjection. A–P, anterior–posterior axis; D, depth from the surface of the skull. (e) The fixation device designed specifically for stereotaxic microinjection in adult zebrafish. Top left, schematic 2D diagram of the device. Top right, a fish undergoing microinjection. Bottom left, 3D rendering of the device. Bottom right, close‐up of zebrafish held in place using the device during microinjection. The asterisk indicates the reference position (lambda) used to locate the IPN. (f) Genomic structure of the vesicular stomatitis virus (VSV) vectors. Both VSV vectors contain four essential proteins—N, P, M, and L. VSV‐mCherry and VSV(RABV‐G)‐GFP encodes the VSV‐G gene or the RABV‐G gene in their genome that allows them to spread trans‐synaptically in anterograde or in retrograde directions, respectively.

The IPN is conserved among vertebrates. The mammalian IPN is more complex, containing seven subnuclei in rodents (Lenn and Hamill 1984), whereas in zebrafish, the IPN can largely be subdivided into three subregions based on its afferent inputs from the dorsal Hb (dHb; the zebrafish homolog of the mammalian HbM): dorsal (dIPN), intermediate (iIPN), and ventral (vIPN) (Figure 1a–c) (Aizawa et al. 2005; Gamse et al. 2005; Amo et al. 2010), although there are studies in which the IPN has been further subdivided (deCarvalho et al. 2014).

In zebrafish, the Hb can be divided into two subnuclei: the dorsal habenula (dHb) and the ventral habenula (vHb) (Figure 1a–c), with the dHb and vHb corresponding to the mammalian medial and the lateral habenula (MHb and LHb), respectively. The dHb can be further divided into two different subnuclei: the lateral subregion (dHbL) and the medial subregion (dHbM). The dHbL projects to the dorsal and intermediate parts of the IPN (dIPN and iIPN, d/iIPN), whereas the dHbM projects to the ventral and intermediate parts of the IPN (vIPN and iIPN, v/iIPN). The dHb is known to show conspicuous left–right asymmetry—in the left dHb, the dHbL is larger than the dHbM; in the right dHb, the dHbM is larger than the dHbL. Due to this size asymmetry between the dHbL and dHbM, the left dHb appears to mostly project to the dIPN, while the right dHb appears to mostly project to the vIPN (Aizawa et al. 2005; Gamse et al. 2005). The vHb directly projects to the ventral–anterior corner of the median raphe (MR) (Amo et al. 2010).

Axons from the dIPN project bilaterally in the dorsal direction, through the region putatively corresponding to the dorsal raphe (DR) (Agetsuma et al. 2010). They further extend laterally around the medial longitudinal fascicle (MLF) and then turn caudally and elongate through the longitudinally extended region known as the dorsal tegmental area (DTA) underlying the rhombencephalic ventricle (RV), forming a bilateral, fasciculated fiber bundle. The DTA is a periventricular structure that broadly includes the griseum centrale (GC), which corresponds to the mammalian periaqueductal gray (PAG), the dorsal tegmental nucleus (DTN), and the nucleus incertus. In contrast, the vIPN has a reciprocal neural connection with the MR (Agetsuma et al. 2010).

The IPN, PAG, and MR have been implicated in the control of behaviors under fear or stress conditions, suggesting that the dHb–IPN pathways may contribute to the modulation of fear responses both in zebrafish (Agetsuma et al. 2010; Lee et al. 2010; Jetti et al. 2014; Chou et al. 2016; Duboué et al. 2017) and in mammals (Koutsikou et al. 2017; Molas et al. 2017; Metzger et al. 2021; Liang et al. 2024; Handa et al. 2025; Matsumata et al. 2025).

In cued fear conditioning, the first encounter with an unconditioned stimulus paired with a conditioned stimulus induced in both control and genetically dHbL‐silenced fish freezing at the same level; such responses generally waned in control fish during memory retrieval. On the contrary, the tendency of dHbL‐silenced fish to freeze increased in comparison to that of control fish during the retrieval session. Following the learning of cued fear conditioning, control sibling fish showed enhanced turning response, suggesting an increase in agitation or flight behaviors (Agetsuma et al. 2010). These results suggest that the dHbL–d/iIPN pathway is essential for fish in making the experience‐dependent behavioral transition from passive (freezing) to active coping (agitation) in response to aversive stimuli. In conditioned place avoidance learning, ablation of the dHbL caused fish to fail in adapting to a changing learning rule, whereas control animals exhibited rapid adaptation (Palumbo et al. 2020). These observations suggest that the dHbL–d/iIPN pathway plays a central role in switching strategies while integrating new evidence with prior experience.

In goal‐directed navigation, the choice between the strategies of depending on landmarks or on dead reckoning allows for the conceptual division of information with regard to source differences. In landmark navigation, animals perceive external cues through primary sensory modalities such as olfaction, hearing, and vision, whereas dead reckoning relies on an integrated internal signal such as vestibular input and motor command. Intriguingly, dHbL‐impaired fish showed a specific defect in learning through the exploitation of internal directional information, while its capacity to learn through external cued information was retained (Cherng et al. 2020; Okamoto et al. 2021). In conjunction with this result, a recent study using a brain‐wide functional screen revealed that the IPN circuit may encode the left–right turning rate in zebrafish larvae; during perceptual decision‐making tasks, fish responded to leftward or rightward moving dots projected on a screen with varying directional coherence (Dragomir et al. 2019; Petrucco et al. 2023; Palieri et al. 2024). These observations suggest that the dHbL–IPN pathway may function to either encode or predict the appropriate internal (interoceptive) and behavioral (motor output) states in response to visual information input, such as the sight of a turning corner or moving dots on the screen when dead reckoning should be activated (Okamoto et al. 2021).

In contrast to the dHbL, the dHbM–v/iIPN–MR pathway is implicated to be essential in homeostatic navigation from an unfavorable to an optimal environment in larval zebrafish, suggesting that this pathway directs fish to become more attentive to its evaluation of the external environment (Okamoto et al. 2021; Palieri et al. 2024).

Dyadic fights in male zebrafish proceed stereotypically, starting with the exhibition of display behaviors from each animal. This is followed by circling and biting attacks and ends when one fish shows fleeing behavior to indicate surrender. Within 24 h following the initial fight, the winner and loser fish continue their respective tendencies to win or lose upon encountering a fight with naïve fish, showing that they maintain altered brain states. The dHbL–d/iIPN–DTA pathway is intensely potentiated in the winner fish; in contrast, intense potentiation is observed in the dHbM–v/iIPN–MR pathway in the loser fish. Furthermore, dHbL‐silenced fish show a significantly higher tendency to lose fights, whereas dHbM‐silenced fish become more resilient to surrender and tend to win. These findings demonstrate that the dHbL–d/iIPN–DTA pathway and the dHbM–i/vIPN–MR pathway are responsible for the behavioral characteristics of the winners and losers, respectively (Chou et al. 2016).

Given the significance of the Hb–IPN pathways in multimodal regulation, it is essential to comprehensively characterize the connections of this circuit from the IPN in adult zebrafish. To achieve this primary goal, we used two tracing methods—viral tracers and a lipophilic dye. In viral tracing, replication‐competent viruses replicate in the initially infected cells and produce infectious virions, which are then transmitted to connected cells, revealing even long‐distance, indirect connections (Nassi et al. 2015). Using adult zebrafish, recombinant vesicular stomatitis virus (VSV) (Beier et al. 2011) or herpes simplex virus (HSV) (Satou et al. 2022) was injected into the dorsal and intermediate subnuclei of IPN or the GC, a region known to be directly connected to the d/iIPN in zebrafish (Agetsuma et al. 2010).

This modern approach to the tract tracing of the IPN is the first step toward understanding its neural mechanism and has revealed its connectivity in a great variety of brain regions in zebrafish, allowing us to infer the functions of the IPN in future investigation. The high degree of conservation among vertebrates also enables the IPN projectome in zebrafish to be used as a foundation for studies in other model organisms.

## 2 Materials and Methods

### 2.1 Animals

A total of nine adult zebrafish (*Danio rerio*) of both sexes were used in the present study. They ranged from 2.5 to 2.7 cm in body length. This study used adult wild‐type (WT) zebrafish (RIKEN‐Wako, Saitama, Japan) (Sadamitsu et al. 2025). Fish were maintained in 7‐L tanks with continuous water exchange at 28.5°C under 14‐h light/10‐h dark cycling. All methods described here have been reviewed and approved by the Animal Care and Use Committees of RIKEN Center for Brain Science.

### 2.2 Virus Construction and Preparation

For anterograde tracing, replication‐competent VSV‐mCherry (1.0 × 10^12^ iu/mL) was used. The VSV vector, pVSV‐mCherry (Figure 1f), was based on pVSV Venus VSVG gifted from by Connie Cepko (RRID:Addgene_36399, Addgene plasmid # 36399) (Beier et al. 2011). It was generated from pVSV Venus VSVG through the replacement of the first Venus position with mCherry by using the XhoI and MscI sites with the following primers:

SalI_mCherry_U:

5ʹ‐aagtcgacGATCCACCGGTCGCCACCATGGTGAGCAAGGGCGAGGA‐3ʹ

mCherry_NruI_L:

5ʹ‐aatcgcgaGCACCTGAGGAGGGCGGCCGCTTTACTTGTACAGCTCGTCCA‐3ʹ

Based on the rescue protocol for rabies virus (Wickersham et al. 2010), VSV was rescued with the VSV support plasmids pCAG‐VSV‐N, ‐P, and ‐L gifted by Ian Wickersham (RRID:Addgene_64087, RRID:Addgene_64088, RRID:Addgene_64085, Addgene plasmid # 64087, # 64088, and # 64085) and T7opt in pCAGGS gifted by Benhur Lee (RRID:Addgene_65974, Addgene, plasmid #65974) (Yun et al. 2014). Human 293T cells were plated at 6.0–7.0 × 10^6^ cells per well in 150‐mm dishes, and medium was added to make up a total of 22.5 mL. The following day, the cells were transfected with a mixture of the following plasmids: pVSV‐mCherry 45.9 µg, pCAG‐VSVN 23.0 µg, pCAG‐VSVP 11.6 µg, pCAG‐VSVL 11.6 µg, and T7opt in pCAGGS 13.8 µg. PEI MAX (Polysciences, #24765‐1) was used for transfection following the manufacturer's instructions. After 72 h, virus supernatants were collected at 24‐h intervals for 4 days and filtered through a 0.45‐µm filter. The collected supernatants were combined and ultracentrifuged at 20,000 rpm for 3 h with an SW28 rotor. For titer determination, concentrated virus solutions were applied in a dilution series to 293T cells. The number of infected cells was examined 24 h following the application of the viruses (Wickersham et al. 2010).

For trans‐synaptic retrograde tracing, replication‐competent VSV(RABV‐G)‐GFP (titer not determined) was used. The virus production and purification were performed as described above with the plasmid pVSV eGFP RABV‐G (Figure 1f), which was gifted from Connie Cepko (RRID:Addgene_31833, Addgene plasmid #31833) (Beier et al. 2011), instead of pVSV‐mCherry.

For monosynaptic retrograde virus tracing using HSV‐GFP, the HSV vector LT‐HSV‐EGFP (RN800; 5.0 × 10^9^ iu/mL), which was established by Rachael L Neve and obtained from the Massachusetts General Hospital (MGH) Gene Delivery Technology Core (USA), was used (Satou et al. 2022).

All procedures utilizing virus vectors have been approved by the Animal Care and Use Committees of RIKEN Center for Brain Science and MEXT.

### 2.3 In Vitro Microinjections

Adult zebrafish were deeply anesthetized with 0.02% tricaine (ethyl 3‐aminobenzoate methane sulfonate salt, Sigma–Aldrich, MO). Following the cessation of gill movements, fish were mounted onto a 3D‐printed stereotaxic apparatus (Figure 1e). The head was held in place by two rods on each side of the pterotic bone and the top edge of the opercle, with a pair of body holders holding the body straight. The incline of the fish's head was adjusted by a height‐adjustable mouthpiece from which Ringer's solution was circulated through the gills. A 0.02% tricaine solution was continuously circulated throughout the system with a peristaltic pump to maintain aestheticization and to provide oxygen.

Due to the absence of stereotaxic coordinates in the adult zebrafish brain atlas, the anatomical location of the IPN and GC was refined empirically (Table 3). The interface between the coronal suture (bregma) and the sagittal suture (lambda) at the top of the skull was chosen as the zero point in referencing the rostrocaudal position of the IPN. From the reference point, the microdrill (World Precision Instruments, FL) was advanced approximately 80 µm rostrally depending on fish size, and a tiny burr hole was made in the skull (Figure 1d) (see coordinates in Table 3).

Glass micropipettes (GD‐2; Narishige, Japan) were pulled with a pipette puller (Sutter Instrument, CA) and polished with a microgrinder (Narishige, Japan) to achieve a tip diameter of 60 µm for all microinjections. Its non‐pulled end was connected to the needle of a microliter syringe (Hamilton Company, NV) with soft‐walled tubing and back filled with Fluorinert (3 M, MN). Using the microliter syringe, the micropipette was then front filled with the virus solution (VSV‐mCherry: diluted 1:1 with Ringer's solution; HSV‐GFP: diluted 1:3 with Ringer's solution; VSV(RABV‐G)‐GFP: diluted 1:10 with Ringer's solution) and inserted through the burr hole to the IPN using a micromanipulator (Narishige, Japan). The insertion depth of the micropipette ranged between 1250 and 1400 µm.

Microinjections were controlled by a microsyringe pump (World Precision Instruments, FL) at a rate of 10 nL/min, and each injection delivered 50–100 nL of VSV solution to the brain. The micropipettes were left in position for 5 min, after which they were slowly retracted. A small piece of ion‐beamed Teflon sheet (Takahashi, Suzuki, Ujiie, Hori, et al. 2007; Takahashi, Suzuki, Ujiie, Iwaki, et al. 2007) was placed over the burr hole and affixed with tissue glue (Aron alpha A; Daiichi‐Sankyo, Japan). 0.02% tricaine solution was then switched out for Ringer's solution to discontinue the administration of anesthesia and to allow the fish to regain consciousness.

Following recovery, fish were housed in individual tanks of Ringer's solution at room temperature for 5 days when tracing with VSV and at 37°C for 6 days when tracing with HSV.

### 2.4 Fixation and Slice Preparation

On the fifth postoperative day for VSV‐injected fish and on the sixth for HSV‐injected fish, fish were deeply anesthetized with 0.1% tricaine and perfused intracardially with 0.1 M Ringer's solution. Fish were then decapitated and fixed overnight in 4% paraformaldehyde (PFA) in 0.1 M phosphate‐buffered saline (PBS; pH 7.4). The brain was then removed and embedded in an agarose solution (3% agarose, 4% sucrose in PBS), which was then cryoprotected with 30% sucrose in PBS at 4°C. The agarose blocks were mounted onto the specimen holder with an optimal cutting temperature compound (Sakura Finetek, Japan), and the brains were sliced coronally in 80‐µm‐thick serial sections using a cryostat (HM525 NX; Thermo Scientific, MA) and collected in PBS.

### 2.5 Immunohistochemistry

#### 2.5.1 For Viral Tracing

Sections were permeabilized in PBS containing 0.1% Triton X‐100 (PBST) for 15 min at room temperature and incubated overnight (20 h) at 4°C with the primary antibodies (see Table 1 for characterization of the primary antibodies used) in 1% blocking solution (Roche, Switzerland) in PBST. After four washes in PBST (20 min each), sections were incubated overnight with the appropriate secondary antibodies (see Table 2 for characterization of the secondary antibodies used) and 4ʹ,6‐diamidino‐2‐phenylindole (DAPI) at 4°C. Sections were washed in PBST, wet‐mounted using ProLong Diamond Antifade Mountant (Invitrogen, MA), and cover‐slipped. A different emission wavelength was chosen for the secondary antibody to differentiate between the native fluorescence imparted from the expression of mCherry or GFP and the amplified signal from immunohistochemistry.

**TABLE 1:** Primary antibodies used.

Antibody	Antibody target	Manufacturer, cat. no., RRID, species	Dilution
DsRed polyclonal	mCherry	Takara Bio Cat# 632496, RRID:AB_10013483, rabbit	1:1000
GFP polyclonal	GFP	Aves Labs Cat# GFP‐1020, RRID:AB_10000240, chicken	1:1000
GFP polyclonal	GTP	Aves Labs Cat# GFP‐1010, RRID:AB_2307313, chicken	1:5000

**TABLE 2:** Secondary antibodies used.

Antibody	Fluorescent label conjugate	Manufacturer, cat. no., RRID, species	Dilution
Anti‐rabbit IgG	Alexa Fluor 488	Thermo Fisher Scientific Cat# A‐11008,RRID:AB_143165, goat	1:500
Anti‐chicken IgG	Alexa Fluor 647	Thermo Fisher Scientific Cat# A‐21244,RRID:AB_2535812, goat	1:500
Anti‐rabbit IgG	Cy3	Jackson ImmunoResearch Labs, Cat# 711‐165‐152,RRID:AB_2307443, Donkey	1:1000
Anti‐chicken IgG	Alexa Fluor 488	Jackson ImmunoResearch Labs, Cat# 703‐545‐155,RRID:AB_2340375, Donkey	1:1000

#### 2.5.2 For Transgenic Lines *Tg(gfap:dTomato)* and *Tg(pkd2l1:GAL4; UAS:GFP)*

Sections were permeabilized in PBST for 10 min and incubated in 1% blocking solution for 60 min. They were then incubated overnight at 4°C with the primary antibodies (see Table 1 for characterization of the primary antibodies used) in 1% blocking solution (Roche, Switzerland) in PBST. After three washes in PBST (10 min each), sections were incubated overnight at 4°C with the appropriate secondary antibodies (see Table 2 for characterization of the secondary antibodies used). Sections were washed in PSBT, wet‐mounted using glycerol, and cover‐slipped.

### 2.6 Lipophilic Dye Injection

Adult zebrafish were perfused, decapitated, and fixed overnight in 4% PFA in PBS. The brain was then removed and embedded in an agarose solution (3% agarose, 4% sucrose in PBS). The agarose block was then sliced in half just rostral to the approximate location of the d/iIPN as recognized by its anatomical structure (caudal endpoint of the midline of the left and the right optical tectum). The brain was then cryoprotected with 30% sucrose in PBS at 4°C and mounted onto the specimen holder with an optimal cutting temperature compound (Sakura Finetek, Japan). It was then sliced coronally using a cryostat (HM525 NX; Thermo Scientific, MA) until the d/iIPN is exposed.

Glass capillaries (GD‐1; Narishige, Japan) were filled with dissolved DiI and left to air‐dry. Using a micromanipulator (Narishige, Japan), DiI was injected into the d/iIPN for a few seconds under a stereo microscope. The injected brains were then incubated in PBS at 37°C for 7–14 days and sectioned afterward as described in section 2–4.

### 2.7 Microscopy

Immunofluorescence was visualized using a NIKON C2‐DUVB confocal laser‐scanning microscope (Nikon, Japan) with 4x, 10x, and 20x objective lenses. The emission wavelength for DAPI was set to 425–475 nm, AlexaFluor 488 to 503–530 nm, and mCherry to 560–602 nm. Images were acquired with the Nikon NIS‐Elements AR (Ver 5.21.03) software. Due to the intense fluorescence of the images, representative *z*‐stacks that best exhibit the trajectory of neurites were chosen and processed through the maximum projection algorithm in Fiji ImageJ. This was done to display the maximum intensity values such that finely labeled structures could be visualized.

Schematic diagrams were illustrated on the Clip Studio Paint 2.0 (Celsys, Tokyo, Japan) software.

### 2.8 Cytoarchitectural Identification and Nomenclature

The adult zebrafish brain atlas (Wullimann et al. 1996) was used as a reference in identifying the anatomical structures and in the neuroanatomic terminology used. Some of the nomenclature employed in the present study was modified for ease of use.

Due to the trans‐synaptic properties of the viral tracers, it is difficult to discern between axons and dendrites; as such, labeled projections are referred to as “neurites” and labeled axonal projections are referred to as “fibers.”

## 3 Results

In the present study, the anterograde connections of the d/iIPN, iIPN, and GC and the retrograde connections of the d/iIPN and iIPN were determined by the expression pattern of the fluorescent protein expressed by the viral tracers and the amplification of the tracer signal through immunohistochemistry staining.

Injection sites were defined as the regions exhibiting the strongest tracer signal intensity. Although we found that signal strength was variable among individuals, the injection sites where primary viral infection took place consistently showed the highest levels of native fluorescence of the viral tracer. The success of viral tracer delivery to the target nuclei was judged by the localization and the expression level of the fluorescent reporter protein. Due to the trans‐synaptic feature of viral tracing, the tracing direction could be intermingled. The terms “neurites” or “fibers” were used to prevent any potential incorrect interpretations. “Neurites” were used when we were unsure whether the structures were dendrites or axons, and “fibers” when we are more confident that they are axons.

All figures are oriented with dorsal upward, and a consistent left–right orientation is maintained throughout all samples.

### 3.1 Anterograde Tracing

Red represents the native fluorescence from the anterograde tracer, and green represents the amplified tracer signal through immunohistochemistry staining.

#### 3.1.1 Anterograde Trans‐Synaptic Tracing From d/iIPN

In the present study, VSV‐mCherry was injected into the d/iIPN as a trans‐synaptic anterograde tracer. We included only cases where the injection site was mostly restricted to the dorsal and intermediate subnuclei. Two samples were obtained, and representative images of the labeled regions were chosen.

##### 3.1.1.1 IPN and Regions Caudal to IPN

**FIGURE 2:**
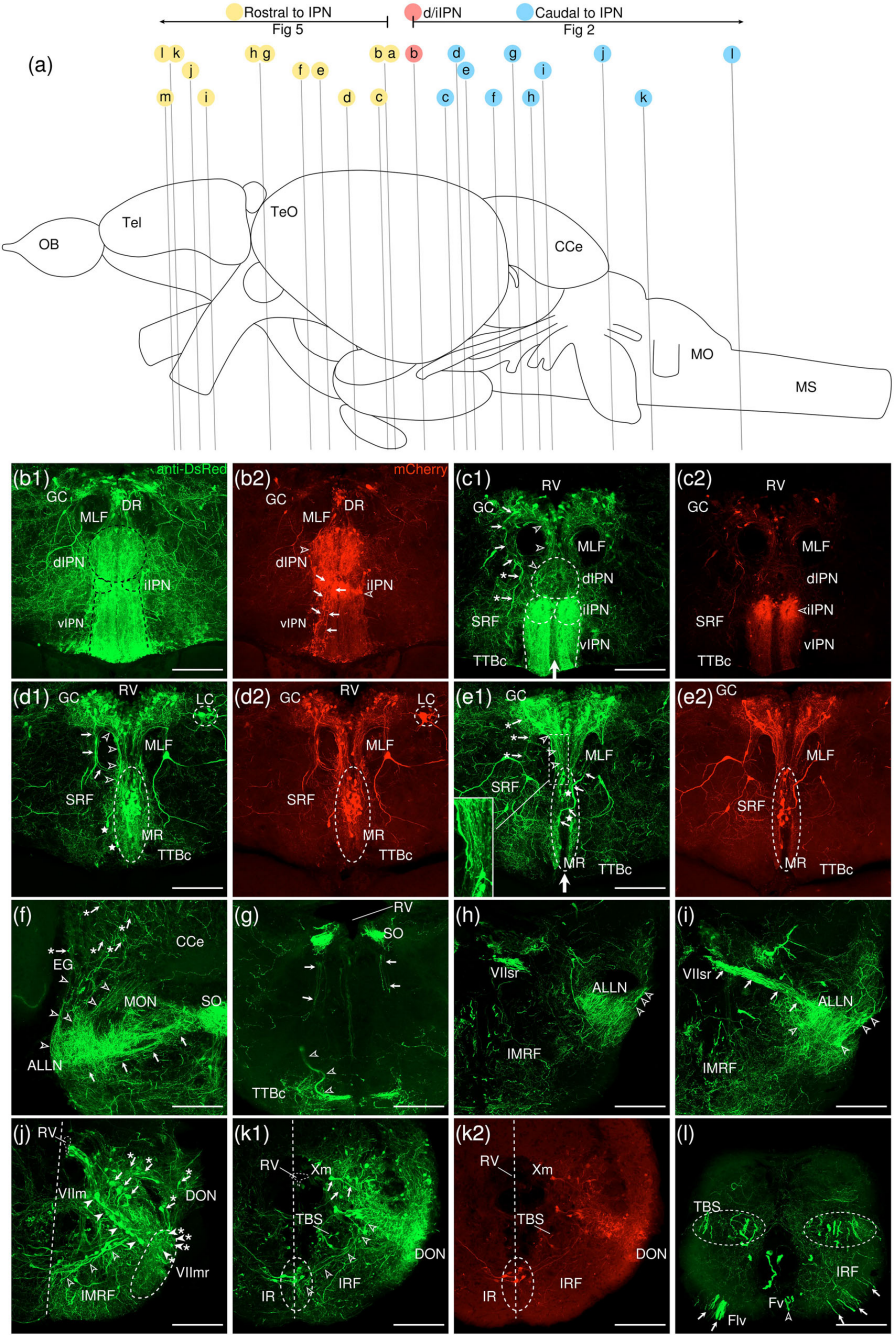
Anterograde trans‐synaptic tracing from the d/iIPN using VSV‐mCherry; regions caudal to IPN. (a) Schematic diagram of the adult zebrafish brain indicating the level of sections (adapted from Wullimann et al. 1996). Red denotes the injection site at the d/iIPN (2b); blue denotes the regions caudal to the IPN (2c–l); yellow denotes the regions rostral to the IPN (5a–m). (b–l) Coronal sections of the adult zebrafish brain showing labeled areas in the d/iIPN (b) and regions caudal to the IPN (d–l) following injection of the trans‐synaptic anterograde tracer VSV‐mCherry in the d/iIPN. Pictures are confocal optical projections of serial cryotome‐cut coronal sections immunostained for mCherry (red; b2, c2) with anti‐DsRed (green; b1, c1, d–l). Figures are arranged in rostrocaudal order from the IPN. All panels are oriented with dorsal upward. For abbreviations, see the list. (b, c) Level of the IPN. Dotted outline in (b1) and (c1) shows the IPN. Arrowheads in (b2) and (c2) refer to the injection site of VSV‐mCherry at the dIPN and iIPN. Arrows in (b2) point to fibers from the iIPN projecting to the vIPN. Arrows with asterisk in (c1) point to neurites from the SRF entering the GC; arrowheads in (c1) point to neurites from the rostral GC extending into the dIPN; and the filled arrow in (c1) points to the gap along the midline in the vIPN. (d, e) Level of the MR and GC. Dotted outline in (d) and (e) shows the MR (center) and in (d) shows the LC (right). Arrows in (d) point to neurites from the GC that travel around the outer border of the MLF to enter the MR; arrowheads point in (d) to neurites from the GC that extend ventrally into the MR; and stars in (d) refer to somata located at the ventrolateral edge of the MR. Arrows in (e) point to SRF neurites entering the MR; arrows with asterisk point to SRF neurites entering the GC; arrowheads point to MR neurites that can be traced to the GC; and the large arrow refers to the midline gap in the MR that is devoid of labeled fibers. (d2, e2) Pictures prior to immunostaining show labeled areas following injection of the trans‐synaptic anterograde tracer VSV‐mCherry in the iIPN to show the expression of the mCherry. (f) Arrows point to neurites from the SO that extend into the ALLN; arrows with asterisk point to fibers in the EG; and arrowheads point to neurites from the ALLN entering the MON and EG. (g) Arrows point to ventrally projecting neurites from the SO. (h, i) Level of the ALLN showing its hallmark morphology of parallel fibers compactly organized in a diagonally oriented pattern. Arrowheads in (h, i) point to the small somata intermingled with fibers in the ALLN. Arrows in (i) point to the VIIsr extending into the ALLN. (j) Dotted outline at the bottom right shows the VIImr, and dotted line shows the midline. Arrows point to the large somata along the VIIm–DON boundary; arrows with asterisk point to the smaller somata in the DON; open arrowheads point to a bundle of fibers studded with somata crossing the midline to the level of the IMRF; filled arrowheads point to somata in the VIIm; and filled arrowheads with asterisk point to somata in the VIImr. (k) Dotted outline shows the IR, and dotted line shows the midline. Arrowheads point to the fibers extending to the level of the IRF and toward the IR, and arrows point to the few somata in the Xm neuropil. (k2) Pictures prior to immunostaining show labeled areas following injection of the trans‐synaptic anterograde tracer VSV‐mCherry in the iIPN to show the expression of the mCherry. (l) Dotted outline shows the TBS. Arrows point to the short, diagonally oriented fibers in the Flv, and the open arrowhead points to a thick neurite in the Fv. Scale bars = 150 µm.

**FIGURE 3:**
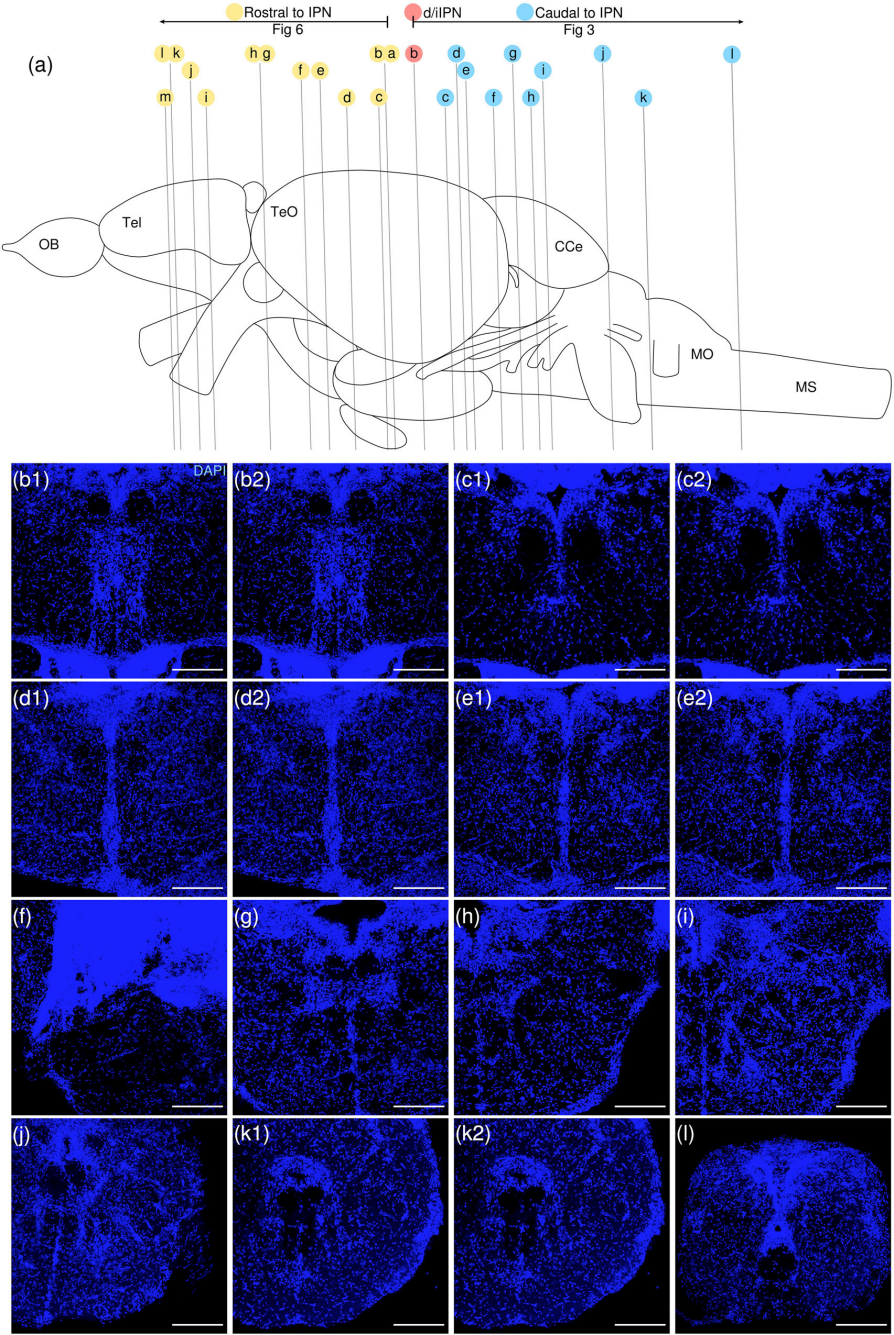
DAPI staining corresponding to Figure 2.

The levels of sections are indicated in Figure 2a. Figure 2b–l shows serial sections arranged in rostrocaudal order from the IPN to the medulla spinalis (MS) (see Figure 3 for counterstaining with the corresponding panel number of Figure 2).

###### 3.1.1.1.1 IPN and GC

The dorsal and intermediate subregions of the IPN (dIPN and iIPN) express the highest levels of mCherry reporter protein, indicating successful delivery and infection of the VSV vector at the target site (Figure 2b2,c2, arrowheads). Somata found in the dIPN and iIPN are small and round. In the iIPN, fibers projecting to the vIPN are present (Figure 2b2, arrows). The vIPN appears to be split into halves along the midline toward its caudal end, with a thin gap where the density of labeled fibers is markedly lower (Figure 2c1, filled arrow).

In the GC dorsal to the IPN, cell somata prominently expressing mCherry are found along the whole length of the nuclei, which extend from the dorsal tegmentum of the midbrain to the hindbrain (Figure 2b–e). However, the distribution of labeled cells in the GC changes rostrocaudally, with cells in the rostral region of the GC loosely organized along the dorsal surface of the nucleus (Figure 2c,d). In contrast, in the caudal region, the distribution of cells gradually shifts ventromedially toward the midline (Figure 2e). A few of the mCherry‐expressing somata in the rostral GC have neurites that extend directly into the dIPN (Figure 2c1, arrowheads).

Throughout the rostrocaudal length of the GC, the GC is characterized by a high density of large, strongly mCherry‐positive somata with neurites that extend around the MLF, virtually encircling it (Figure 2c1,d, arrowheads). The morphology of the labeled somata in the GC appears to vary, with the population in the rostral GC taking on a rounded shape (Figure 2c,d) and the cohort in the caudal GC exhibiting a more elongated, ovoid appearance (Figure 2e).

###### 3.1.1.1.2 Rostral Rhombencephalon

The MR is immediately caudal to the IPN and is located underneath the GC (Figure 2d,e). At the level of the MR, the neurites of the mCherry‐positive somata in the GC form a compact fibrous bundle, which then extends ventrally to enter the MR (Figure 2d, arrowheads). In addition, a separate bundle emerging from the GC travels around the outer border of the MLF (Figure 2d, arrows). The labeled cell bodies in the MR predominantly cluster along the midline at the center of the nucleus (Figure 2d,e), although a few somata stray toward the ventrolateral edge of the MR (Figure 2d, stars). Cell somata in the MR exhibit robust mCherry expression, and some emit neurites with a trajectory that can be traced to the GC (Figure 2e, 1, inset, arrowheads; Figure 2e2). Morphologically, the MR appears as an elongated ovoid nucleus at its rostral section (Figure 2d) that tapers into a narrow fusiform shape toward its caudal end (Figure 2e). At the caudal MR, the nucleus appears to be split into two halves along the midline, similar to observations made in the caudal vIPN. This midline gap is a narrow area characterized by the absence of labeled fibers (Figure 2e, filled arrow), although a few labeled cells are found to be present (Figure 2e, stars).

The noradrenergic locus coeruleus (LC) is located in the isthmal tegmentum at the ventrolateral periphery of the GC (Ma 1994a, 1994b) and manifests as large distinct neurons with ventrolaterally oriented dendrites strongly positive for mCherry (Figure 2d).

Somata in the superior reticular formation (SRF) express mCherry robustly and can be distinguished from their neighbors by their large size and distinct fusiform shape with thick, ramified neurites (Figure 2d,e). These SRF somata intermingle with labeled neurites, although a preference for ipsilateral side is observed (Figure 2c–e). Some of the SRF neurites appear to be connected with the GC (Figure 2c1,e, arrows with asterisk) and the MR (Figure 2e, arrows). Others extend toward the ventral border of the brain, aggregating with the plexus near the crossed tectobulbar tract (= tractus tectobulbaris cruciatus, TTBc) area (Figure 2c–e).

Originating from the optic tectum (= Tectum opticum, TeO), the tectobulbar tract (= tractus tectobulbaris, TTB) runs dorsoventrally and bifurcates toward the medial hindbrain. A bilaterally labeled plexus of fibers bordering the IPN and MR nuclei near the midline is observed in the area of the TTBc at the ventral‐most border of the brain (Figure 2c–e). As the TTBc runs caudalward, thick fibers appear in the area and become more prominent (Figure 2g, arrowheads)

**FIGURE 4:**
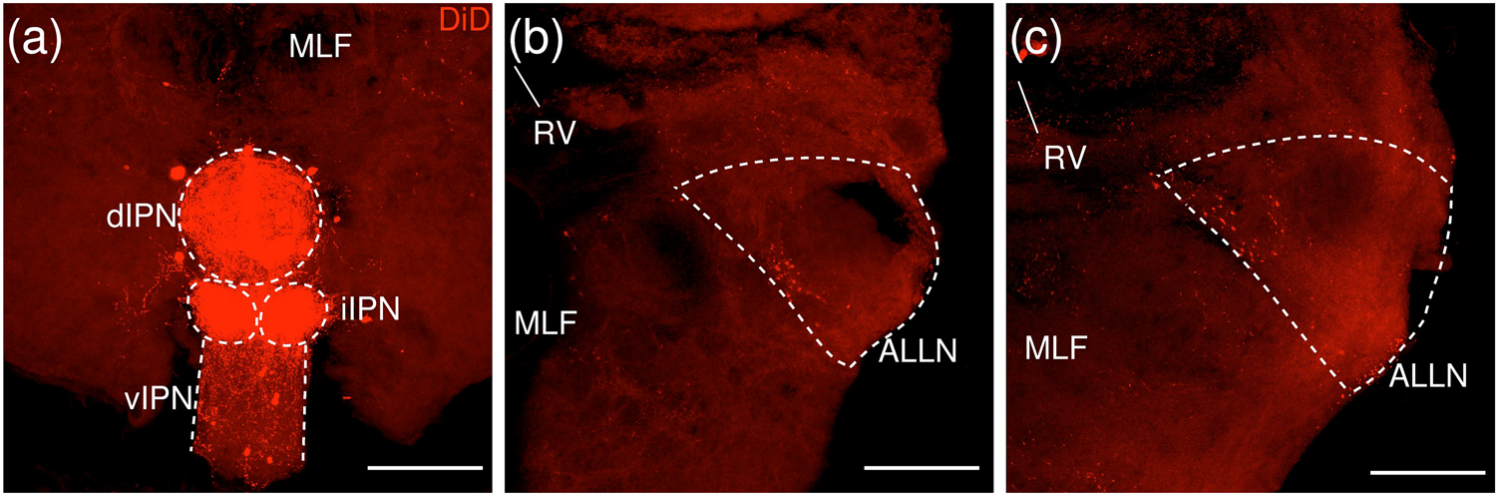
(a–c) Coronal sections of the adult zebrafish brain showing labeled areas following application of the lipophilic tracer DiD (red) to the d/iIPN. Pictures are confocal optical projections of serial cryotome‐cut coronal sections. All panels are oriented with dorsal upward. For abbreviations, see the list. (a) Level of the IPN. Dotted outline shows the IPN. (b, c) Level of the ALLN. Dotted outline shows the ALLN. Scale bars = 150 µm.

The secondary octaval population (SO) (McCormick and Hernandez, 1996) located underneath the RV along the midline exhibits moderate expression of mCherry (Figure 2f,g) and emits fine, dorsoventrally oriented neurites that project ventrally (Figure 2g, arrows). A separate cohort of neurites from the SO sweep out toward the area of the anterior lateral line nerve (ALLN), a bilateral triangular region at the lateral border of the brain (Figure 2f, arrows). The ALLN is distinct in its morphology and is characterized by parallel fibers compactly organized in a diagonally oriented pattern with a diffuse scattering of small, intermingled somata (Figure 2f,h,i, arrowheads). DiI tracing on the d/iIPN shows the labeled axonal terminal in the ALLN, which supports that the d/iIPN neurons may directly innervate the ALLN region (Figure 4). Some of the labeled fibers emerge from the ALLN region to enter the medial octavolateralis nucleus (MON). They eventually radiate dorsally to enter the granular eminence (EG) of the cerebellum (Figure 2f, arrowheads), where fibers studded with numerous irregular varicosities are distributed (Figure 2f, arrows with asterisk). Due to the dense meshwork of neuropils in the region, it is difficult to precisely discern the MON from the ALLN.

On the right side of the brain, the facial sensory root (VIIsr) is robustly positive for mCherry, emerging as a thick bundle of tightly intertwined fibers close to the midline (Figure 2h,i). The VIIsr travels diagonally outward, extending to the dense neuropil in the area of the ALLN (Figure 2i, arrows).

The intermediate reticular formation (IMRF) is likewise labeled, albeit as a much more diffuse plexus (Figure 2h–j).

###### 3.1.1.1.3 Caudal Rhombencephalon and MS

With regard to the far caudal regions of the brain (i.e., medulla oblongata [MO] and MS), data from only one sample were obtained.

The facial nerve motor nucleus (VIIm) expresses mCherry strongly, revealing a cluster of large somata densely packed in a narrow strip that extends diagonally toward the ventrolateral border of the brain (Figure 2j, filled arrowheads), where its root (facial motor root, VIImr) appears interspersed with a few large stomata (Figure 2j, filled arrowheads with asterisk) (Mueller et al. 2004). Although the VIIsr and the VIIm are bilateral structures, labeling is predominantly detected on one side (Figure 2h,i,j).

The dense plexus immediately adjacent to the VIIm is presumed to be the descending octaval nucleus (DON) (Figure 2j). This area contains multiple large somata weakly positive for mCherry lined neatly along the VIIm–DON boundary (Figure 2j, arrows), as well as smaller, sporadically scattered somata (Figure 2j, arrows with asterisk).

A conspicuous bundle of thick fibers studded with round somata crosses the midline at the level of the IMRF (Figure 2j, arrowheads); a similar, albeit less substantial, bundle is observed further caudally to cross the IRF (Figure 2k, arrowheads), extending from the DON to ramify in the region of the inferior raphe (IR), which lies on the ventral midline (Figure 2k, 1). The stomata in the IR, akin to those in the MR, exhibit robust expression of mCherry (Figure 2k2), although they are much smaller in comparison.

The vagal nerve motor nucleus (Xm) flanks the lateral border of the RV (Mueller et al. 2004) and is composed of a neuropil interspersed with a few small somata expressing mCherry (Figure 2k, arrows).

As with the SRF and IMRF, the inferior reticular formation (IRF) contains a labeled plexus of fibers, although no somata are detected (Figure 2k,l). The bulbospinal tract (= tractus bulbospinalis, TBS) emerges adjacent to the IRF (Figure 2k) and expands in size as it runs caudally. At the spinal cord, the TBS is displaced laterally (Wullimann et al. 1996) and multiple thick fibers are seen (Figure 2l).

In the spinal cord, the funiculus lateralis pars ventralis (Flv) contains short, diagonally oriented fibers arranged in parallel (Figure 2l, arrows), while a thick neurite is present in the funiculus ventralis (Fv) (Figure 2l, arrowhead).

##### 3.1.1.2 Regions Rostral to IPN

**FIGURE 5:**
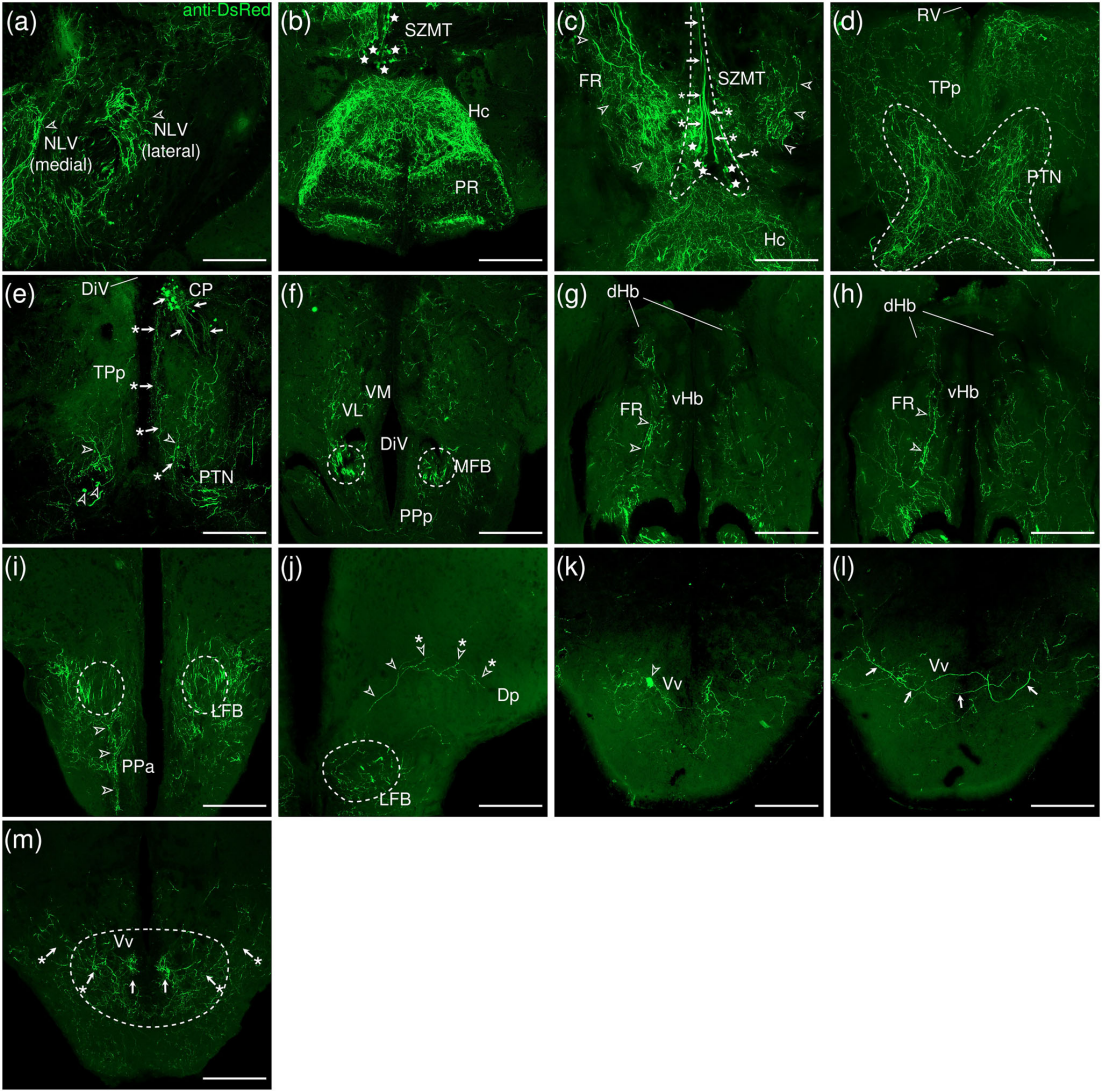
Anterograde trans‐synaptic tracing from the d/iIPN with VSV‐mCherry; regions rostral to IPN. (a–m) Coronal sections of the adult zebrafish brain showing labeled regions rostral to the IPN following injection of trans‐synaptic anterograde tracer VSV‐mCherry in the d/iIPN. Pictures are confocal optical projections of serial cryotome‐cut coronal sections immunostained for mCherry (red) with anti‐DsRed (green). Figures are arranged in caudorostral order from the IPN. All panels are oriented with dorsal upward. For abbreviations, see the list. (a) Arrowheads point to the fibers in the NLV. (b, c) Stars point to somata in the SZMT. (c) Dotted outline shows the SZMT. Arrows point to a labeled fiber bundle travelling along the coronal midline in the SZMT, and arrows with asterisk point to the ramification of the aforementioned fiber bundle as it radiates toward the Hc. (d) Dotted outline shows the butterfly‐shaped plexus in the PTN. (e) Arrows point to the small cluster of somata in the CP and the associated neurites; arrows with asterisk point to fibers in the TPp that run vertically along the midline; and arrowheads point to somata in the PTN. (f) Dotted outline shows the MFB. (g, h) Arrowheads point to the FR. (i) Dotted outline shows the LFB, and arrowheads point to fibers emerging from the area of the LFB to extend ventrally into the PPa. (j) Dotted outline shows the LFB; arrowheads point to the trajectory of the neurite traveling into the Dp; and arrowheads with asterisk point to the arborization of the aforementioned neurite. (k) Arrowheads point to a large somata in the caudal Vv. (l) Arrows point to a neurite that spans both hemispheres of the brain in the Vv. (m) Dotted outline shows the rostral Vv; arrows point to a bilateral distribution of fibers concentrated at the midline; and arrows with asterisk point to the aforementioned distribution of neurites gradually dissipating. Scale bars = 150 µm.

**FIGURE 6:**
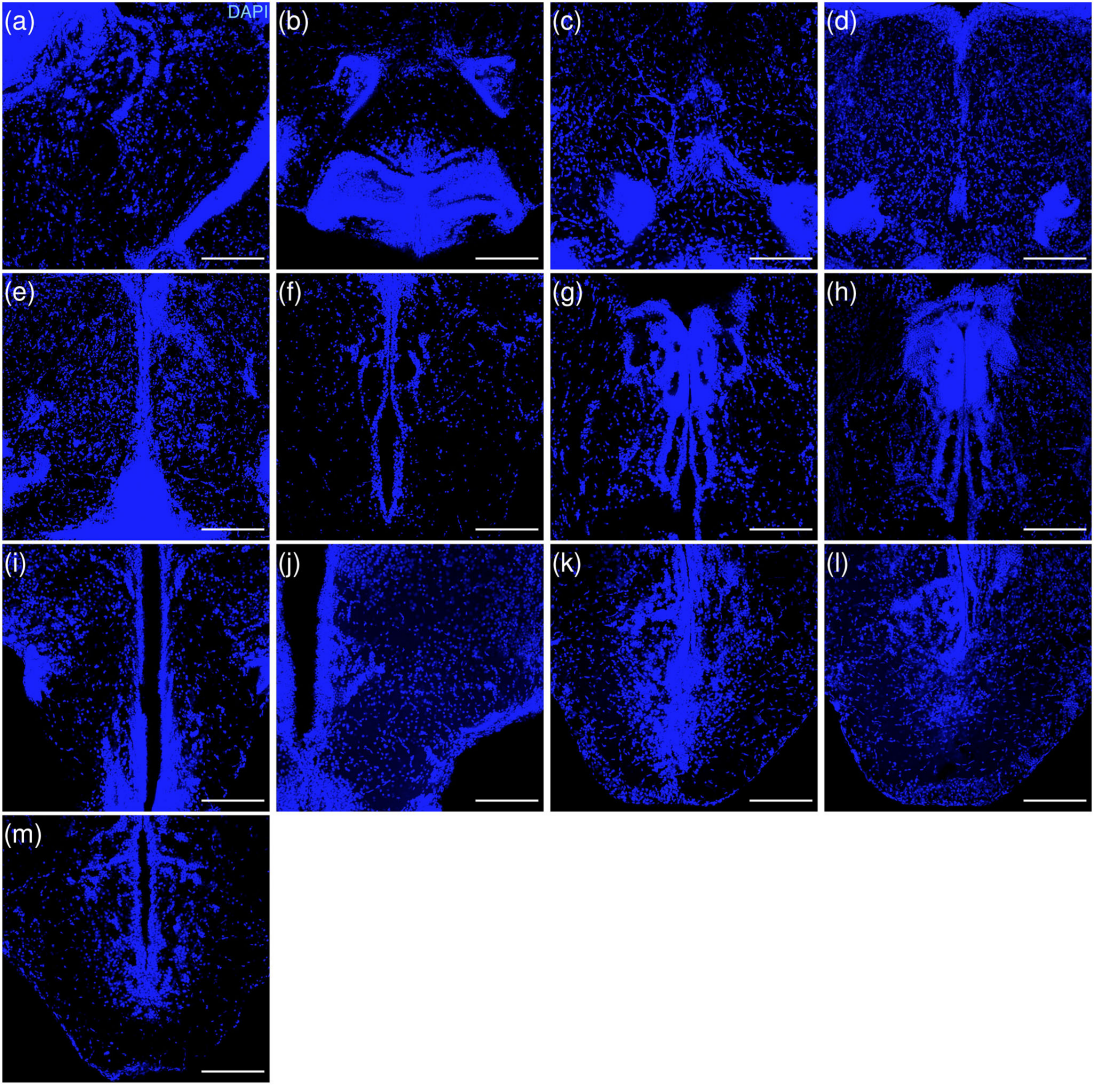
DAPI staining corresponding to Figure 5.

The level of sections is indicated in Figure 2a. Figure 5 shows serial sections arranged in caudorostral order from the IPN to the telencephalon. Expression of mCherry reporter protein is conspicuously weaker in regions rostral to the IPN in comparison to those caudal (see Figure 6 for counterstaining with the corresponding panel number of Figure 5).

###### 3.1.1.2.1 Mesencephalon and Diencephalon

In the coronal plane, the rostral part of the nucleus lateralis valvulae (NLV) is split in each of the hemispheres into medial and lateral regions. Fibers weakly positive for mCherry are present within the confines of both regions of the NLV (Figure 5a, arrowheads).

Labeling in the caudal zone of the periventricular hypothalamus (Hc) is characterized by a dense neuropil of compactly intertwined fibers that outline the trapezoid structure that is the paired posterior recess of the diencephalic ventricle (PR) (Figure 5b,c).

The subventricular zone of mesencephalic tegmentum (SZMT) is a narrow, triangular region located above the Hc. A labeled fiber bundle travels along the coronal midline in a vertical orientation in the SZMT (Figure 5c, arrows) and then ramifies into multiple thick branches that radiate ventrally toward the Hc (Figure 5c, arrows with asterisk). At the level of the multifurcation, several small spherical somata intermingle with the labeled fibers (Figure 5b,c, stars). The bilateral FR at the side of the SZMT is weakly positive for mCherry, although it is observed to be more robustly labeled on the left.

The rostral part of the posterior tuberal nucleus (PTN) is covered by a compact butterfly‐shaped neuropil (Figure 5d, outlined) and sparsely contains small round somata (Figure 5e, arrowheads). Dorsal to the PTN, the central posterior thalamic nucleus (CP) contains a small cluster of somata emitting neurites that project ventrally toward the PTN (Figure 5e, arrows). In between the CP and PTN, labeled fibers in the periventricular nucleus of the posterior tuberculum (TPp) run vertically along the midline and appear to enter the PTN (Figure 5e, arrows with asterisk).

Multiple‐labeled short, thick strands of fibers in the diencephalon are identified as the medial forebrain bundle (MFB) (Figure 5f, outlined) based on their proximity to the posterior part of the parvocellular preoptic nucleus (PPp) bordering the diencephalic ventricle (DiV) and their location under the ventrolateral (VL) and ventromedial thalamic nucleus (VM).

Labeling in the dHb is predominantly found in the dHb on the left and to a lesser extent on the right (Figure 5g). The labeled fibers in the dHb can be traced to the FR; the bilateral but preferentially ipsilateral distribution of labeled fibers in the Hb coincides with the labeling in the FR, which is substantially more robust on the left (Figure 5g,h, arrowheads). There is a conspicuous absence of labeled structures in the ventral Hb (vHb).

###### 3.1.1.2.2 Telencephalon

The lateral forebrain bundle (LFB) is a paired tract containing short multi‐stranded fibers finely positive for mCherry that are arranged vertically in the area of the LFB (Figure 5i,j). Some fibers appear to interweave with the LFB and extend ventrally into the anterior part of the parvocellular preoptic nucleus (PPa) (Figure 5i, arrowheads). A continuous fiber radiates toward the dorsal pallium (Dp) in the telencephalon (Figure 5j, arrowheads), where it arborizes (Figure 5j, arrowheads with asterisk) and enters the posterior zone of the dorsal telencephalic area (Dp).

In the ventral telencephalon, labeled cell somata and a loosely organized plexus of neurites are detected in the ventral nucleus of the telencephalic area (Vv) (Figure 5k,m) rostral to the anterior commissure. The caudal Vv contains a single large soma (Figure 5k, arrowhead) and a continuous neurite that crosses the midline to span both hemispheres (Figure 5l, arrows). In the rostral Vv (Figure 5m, outlined), the bilateral distribution of neurites appears to be concentrated near the midline (Figure 5m, arrows) and gradually dissipates as it spreads outward, becoming more diffuse (Figure 5m, arrows with asterisk).

###### 3.1.1.2.3 Anterograde Trans‐Synaptic Tracing From iIPN

**FIGURE 7:**
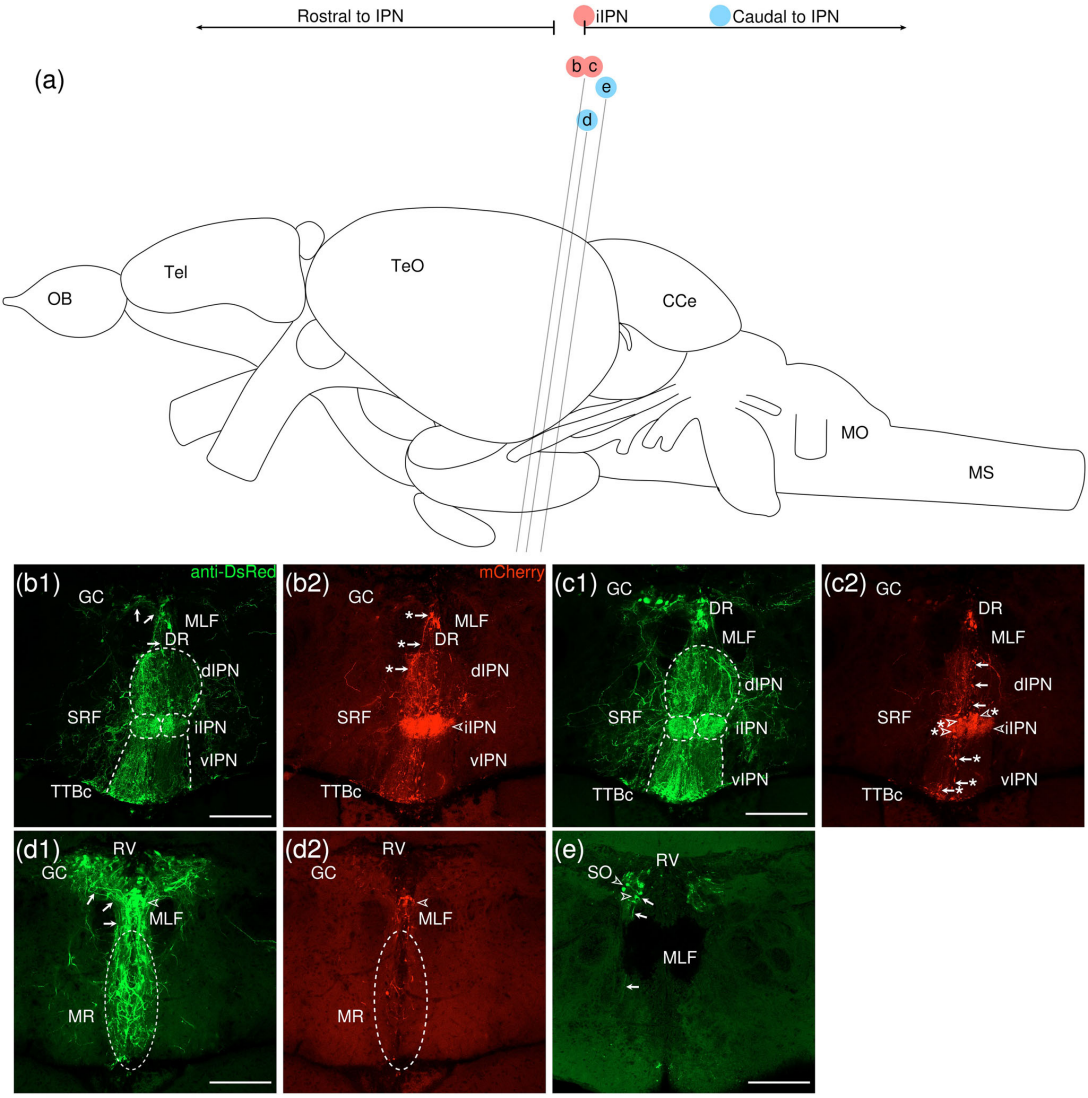
Anterograde trans‐synaptic tracing from the iIPN with VSV‐mCherry. (a) Schematic diagram of the adult zebrafish brain indicating the level of sections (adapted from Wullimann et al. 1996). Red denotes the injection site at the iIPN (b, c); blue denotes the regions caudal to the IPN (d, e). (b–e) Coronal sections of the adult zebrafish brain showing labeled areas in the iIPN (b, c) and regions caudal to the IPN (d, e) following injection of trans‐synaptic anterograde tracer VSV‐mCherry in the iIPN. Pictures are confocal optical projections of serial cryotome‐cut coronal sections immunostained for mCherry (red; b2, c2) with anti‐DsRed (green; b1, c1, d, e). Figures are arranged in rostrocaudal order from the IPN. All panels are oriented with dorsal upward. For abbreviations, see list. (b, c) Level of the IPN. Dotted outline in b1 and c1 shows the IPN, and arrowheads in (b2) and (c2) refer to the injection site of VSV‐mCherry at the iIPN. Arrows in (b1) point to fibers exiting the dIPN to enter the GC, and arrows with asterisk in b2 point to somata in the DR and the associated neurites that enter the dIPN. Arrows in c2 point to the vertically oriented neurites interspersed with small somata in the dIPN; arrows with asterisk point to fibers in the vIPN; and arrowheads with asterisk point to the large round somata in the iIPN. (d) Level of the MR and GC. Dotted outline shows the MR, and arrows point to neurites from the GC that extend toward the MR. (d2) Pictures prior to immunostaining show labeled areas following injection of the trans‐synaptic anterograde tracer VSV‐mCherry in the iIPN to show the expression of the mCherry. (e) Arrowhead point to somata in the SO, and arrows point the associated neurites. Scale bars = 150 µm.

**FIGURE 8:**
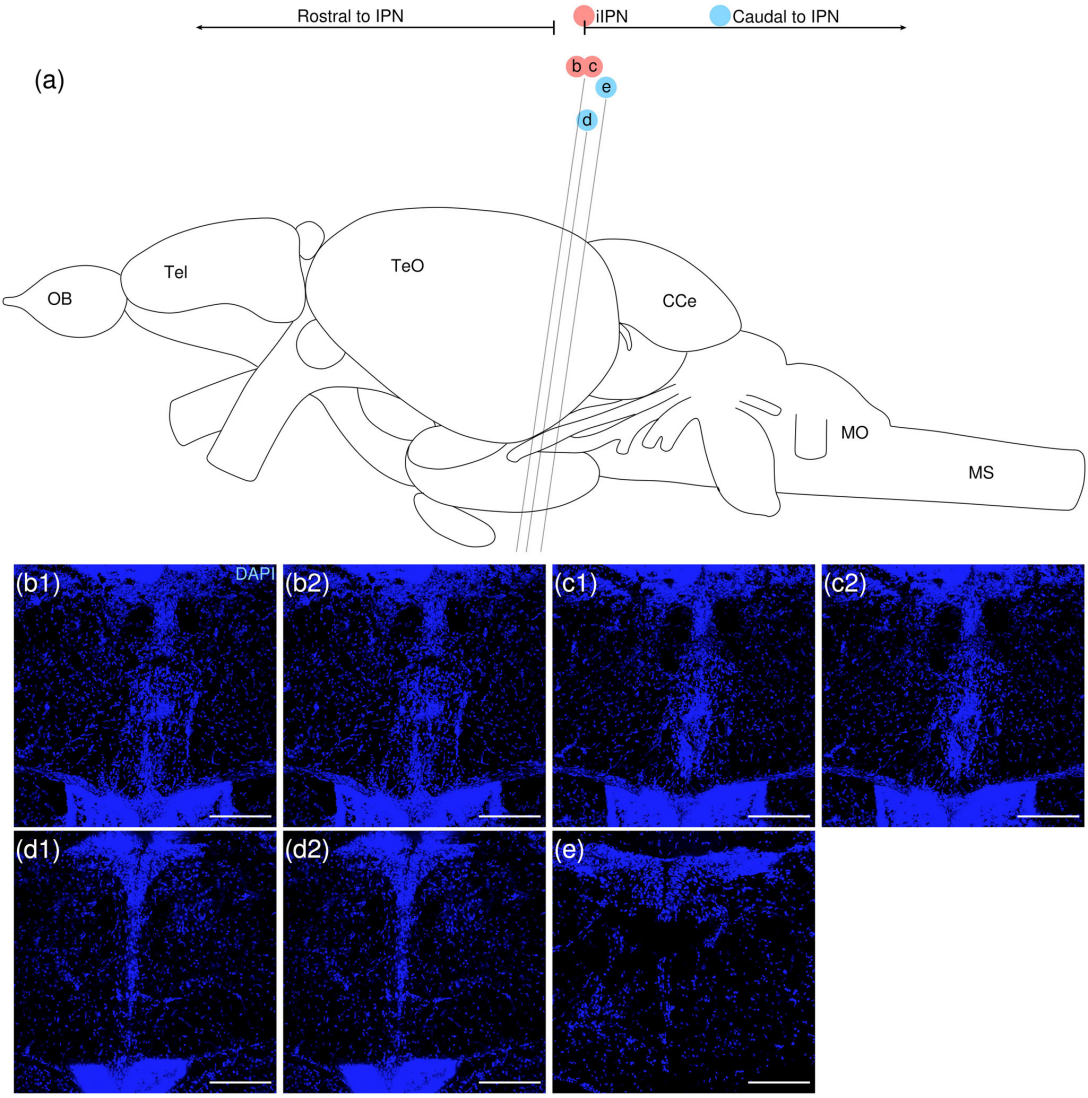
DAPI staining corresponding to Figure 7.

VSV‐mCherry was injected into the iIPN as a trans‐synaptic anterograde tracer. One sample in which strong mCherry expression is virtually restricted to the intermediate subnuclei was obtained. The overall mCherry expression and subsequent amplified signal in this sample are noticeably weaker than in other samples in this study, suggesting that VSV propagation was limited in this particular sample (Figure 7) (see Figure 8 for counterstaining with the corresponding panel number of Figure 7).

The level of sections is indicated in Figure 7a. Figure 7b–e shows serial sections arranged in rostrocaudal order from the IPN.

The injection site at the iIPN expresses high levels of mCherry (Figure 7b2,c2, arrowhead), with several round somata present (Figure 7c, 2, arrowheads with asterisk). The iIPN emits neurites in dorsal, ventral, and lateral directions, with fibers traveling to the dIPN, vIPN, and SRF, respectively.

In the dIPN, vertically oriented neurites interspersed with small somata span the entire subnucleus (Figure 7c, 2, arrows). The triangular DR on top of the dorsolateral edge of the dIPN (Figure 7b,c) contains multiple large somata emitting ventrally projecting neurites that enter the dIPN (Figure 7b, 2, arrows with asterisk). Although the high intensity of fluorescence in the iIPN due to intense mCherry expression has caused difficulty in discerning between afferent and efferent neurites, the neurites present in the dIPN are likely to be a combination of those emitted by somata in the aforementioned triangular region and those originating from the iIPN.

The pattern of neurite projection is more clearly defined in the vIPN, with neurites from the iIPN traveling roughly parallel to each other throughout the extent of the vIPN (Figure 7c, 2, arrows with asterisk). Although labeled neurites and somata are present throughout the dIPN and vIPN, there is a preferential predominance to the left hemisphere. Some neurites radiate laterally from the iIPN and spread out to span the SRF (Figure 7c, 1).

The GC is bilaterally labeled and contains large round somata nestled within a plexus of neurites (Figure 7b–d). Some of the fibers that exit the dIPN appear to make a turn and course into the GC (Figure 7b, 1, arrows). From the GC, some neurites travel ventrally to go around the MLF and toward the MR (Figure 7d, arrows), where somata and thick neurites are densely packed together (Figure 7d, 1). The MR nucleus is very finely positive for mCherry (Figure 7d, 2).

A cluster of mCherry‐expressing somata is present at the peduncle of the ventral GC dorsal to the MR (Figure 7d1,d2, arrowhead). The area these somata are located in appears to be a continuation of the DR identified in Figure 7c with regard to the location, distribution pattern, and relatively robust mCherry expression of the detected somata (Figure 7).

The SO includes several large round somata (Figure 7e, arrowheads), some of which emit weakly labeled neurites that extend ventrally (Figure 7e, arrows). Signal in regions caudal to the SO is faint and sparse even following amplification with immunohistochemistry staining, thus not included in the present study.

#### 3.1.2 Anterograde Trans‐Synaptic Tracing From GC

The GC is reported to receive projections from the dIPN, and a reciprocal connection between the dIPN and GC has been previously suggested (Agetsuma et al. 2010). Its anterior (aGC) and posterior (pGC) sections appear to be anatomically distinct based on the organizational distribution of cells and their morphology as described above (Figure 2c–e). In the present study, VSV‐mCherry was injected into the aGC and pGC as a trans‐synaptic tracer.

##### 3.1.2.1 Anterior GC

**FIGURE 9:**
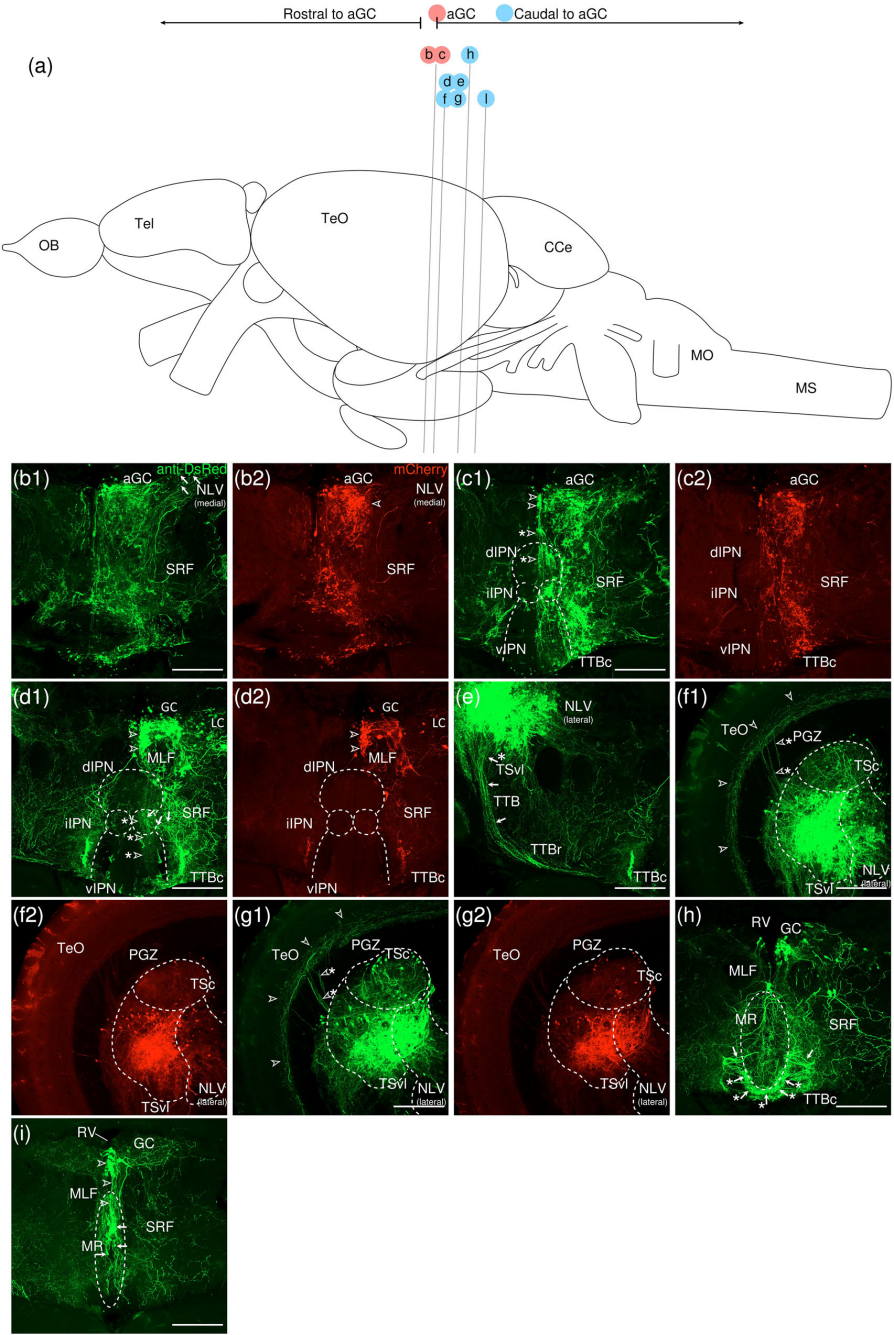
Anterograde trans‐synaptic tracing from the anterior GC with VSV‐mCherry. (a) Schematic diagram of the adult zebrafish brain indicating the level of sections (adapted from Wullimann et al. 1996). Red denotes the injection site at the aGC (b, c); blue denotes the regions caudal to the aGC (d–i). (b–i) Coronal sections of the adult zebrafish brain showing labeled areas in the aGC (b, c) and regions caudal to the aGC (d–i) following injection of trans‐synaptic anterograde tracer VSV‐mCherry in the aGC. Pictures are confocal optical projections of serial cryotome‐cut coronal sections immunostained for mCherry (red; b2, c2) with anti‐DsRed (green; b1, c1, d‐i). Figures are arranged in rostrocaudal order from the aGC. All panels are oriented with dorsal upward. For abbreviations, see list. (b) Level of the aGC. Arrow in (b1) points to large round somata in the NLV, and arrowheads in (b2) refer to the injection site of VSV‐mCherry at the aGC. (c) Level of the aGC and IPN. Dotted outline shows the IPN. Arrowheads in c1 point to somata arranged along the midline, and arrowheads with asterisk point to the neurites of the aforementioned somata. (d1,2) Level of the IPN. Dotted outline shows the IPN. Arrowheads refer to the injection site of VSV‐mCherry at the aGC. Arrows point to the thick neurites connecting the iIPN and the SRF, and arrowheads with asterisk point to the vertically oriented neurites in the iIPN and in the vIPN. (d2) Pictures prior to immunostaining show labeled areas following injection of the trans‐synaptic anterograde tracer VSV‐mCherry in the iIPN to show the expression of the mCherry. (e) Arrows point to the bundle of fibers in the TTB traveling along the edge of the brain, and arrows with asterisk point to the multifurcation of the TTB at the level of the TSvl. (f, g) Dotted outline shows the TSc (top), the TSvl (left), and the NLV (right). Arrowheads point to the narrow strip of neurites along the stratum album centrale layer of the TeO, and arrowheads with asterisk point to the fine neurites emerging from the TSvl to enter the PGZ. (f2, g2) Pictures prior to immunostaining show labeled areas following injection of the trans‐synaptic anterograde tracer VSV‐mCherry in the iIPN to show the expression of the mCherry. (h, i) Level of the MR. Dotted outline shows the MR. Arrows in (h) point to the thick horizontal neurites at the lateral peripheral of the MR, and arrows with asterisk point to the U‐shape around the ventral border of the MR formed by the aforementioned neurites. Arrows in (i) point to the large somata in the caudal MR, and arrowheads point to the GC somata and the associated neurites that enter the MR. Scale bars = 150 µm.

**FIGURE 10:**
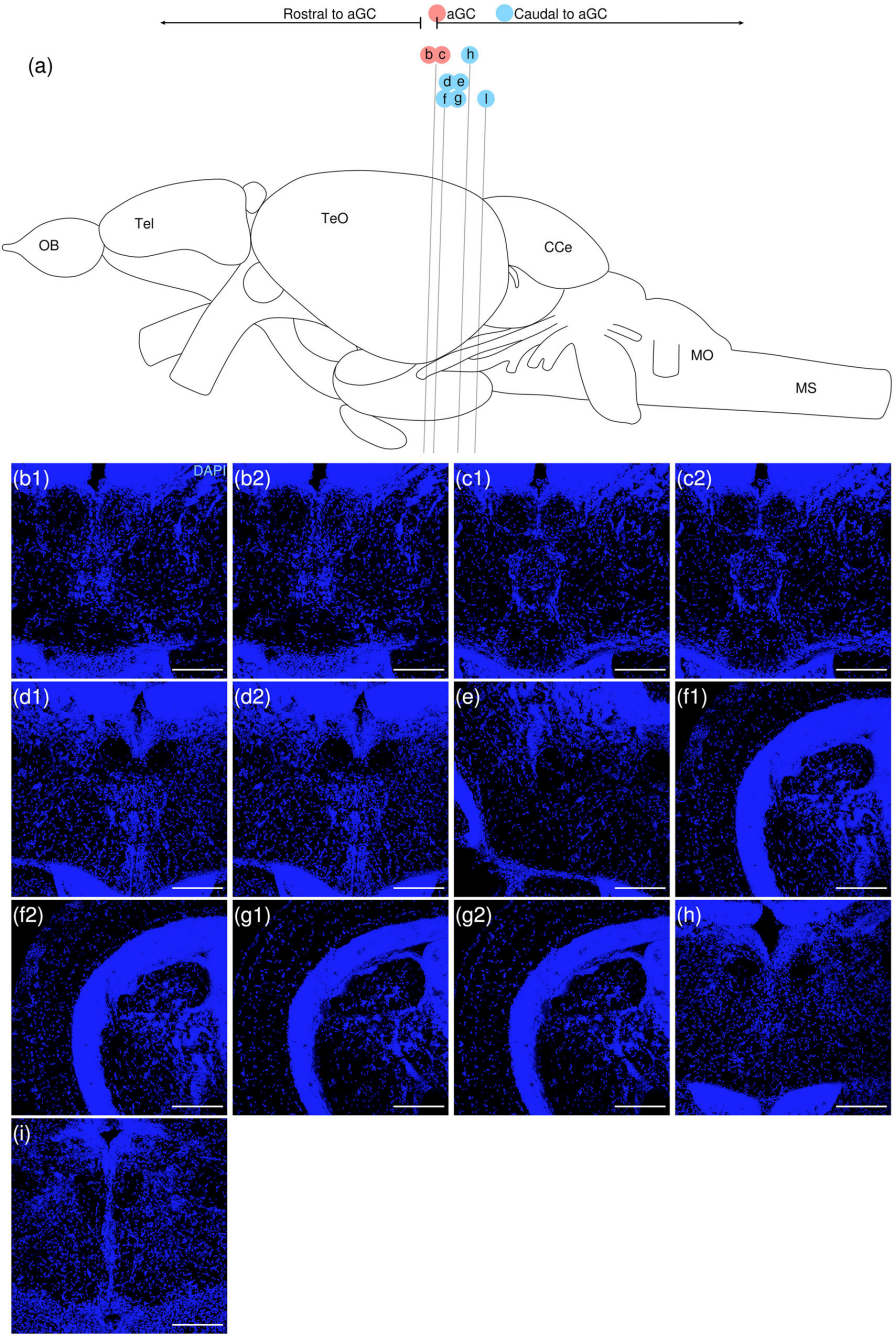
DAPI staining corresponding to Figure 9.

VSV‐mCherry was injected into the aGC as a trans‐synaptic anterograde tracer, and one sample was obtained. A large cluster of somata robustly expressing mCherry in the right aGC was determined to be the injection site (Figure 9b, 2, arrowhead; see Figure 10 for counterstaining with the corresponding panel number of Figure 9).

The level of sections is indicated in Figure 9a. Figure 9b–i shows serial sections arranged in rostrocaudal order from the aGC.

In the coronal plane of the IPN, the majority of somata expressing mCherry are arranged along the midline in a vertical orientation (Figure 9c1,d, arrowheads). Some of the somata in the GC have neurites that extend ventrally into the dIPN (Figure 9c, 1, arrowheads with asterisk). Although vertically oriented neurites are also found in the iIPN and vIPN (Figure 9d, arrowheads with asterisk), it is difficult to determine if they are a continuation of the fibers observed in the dIPN.

At the caudal end of the IPN, neurites emerging from the right GC nuclei transverse around the MLF to encircle it (Figure 9d) and extend continuously into the SRF at the sides of the IPN with an ipsilateral predominance to the side of the VSV‐infected GC. A thick neurite connects the iIPN to the SRF plexus (Figure 9d, 1, arrows). SRF neurites appear to spread into the TTBc, which took on the appearance of two compact strips of strongly mCherry‐positive neurites on both sides of the vIPN (Figure 9d).

The fibrous bundle at the area of the TTBc appears to expand and ramify into multiple thick, horizontally oriented fibers at the periphery of the MR (Figure 9h, arrows), forming a U‐shaped outline around the ventral border of the MR (Figure 9h, arrows with asterisk). The rostral MR appears as a plexus of arborized neurites that is predominantly denser along the midline (Figure 9h,i). Likewise, the caudal MR contains large somata expressing mCherry (Figure 9i, arrows). Several somata in the GC emit neurites that project into the MR nucleus (Figure 9i, arrowheads).

The uncrossed tectobulbar tract (= tractus tectobulbaris rectus, TTBr) runs along the ventrolateral edge of the midbrain and makes a dorsal turn to join the vertically oriented TTB. In the TTB, a coherent bundle made up of long individual fibers arranged in parallel (Figure 9e, arrows) multifurcates at the level of the ventrolateral nucleus of the torus semicircularis (TSvl) (Figure 9e, arrow with asterisk).

The TTB appears to enter the TSvl (Figure 9e), and a few very fine TTB fibers emerge from the dorsolateral surface of the TSvl to penetrate the periventricular gray zone (PGZ) of the TeO as individual fibers (Figure 9f, arrowheads with asterisk, g, arrowheads with asterisk) and spread as a narrow strip consisting of intertwined neurites along the stratum album centrale layer of the TeO (Figure 9f,g, arrowheads).

The TSvl exhibits robust expression of mCherry as a dense neuropil containing a significant population of large cell somata (Figure 9f,g). The meshwork of neurites in the TSvl and the lateral region of the NLV appears to be closely intertwined. The lateral NLV contains somata intermingled in a dense plexus strongly positive for mCherry (Figure 9f,g). The central nucleus of the TS (TSc) contains mCherry‐expressing somata and a dense plexus of fibers (Figure 9f,g), although labeling is sparse in comparison with that detected in the TSvl and lateral NLV.

The medial region of the NLV at the lateral corner of the aGC is likewise labeled, with multiple strands of neurites arranged in a parallel pattern and large round somata present (Figure 9b, 1, arrows).

Most notably, labeling of the TTBr, TTBc, and TS is observed only in the left hemisphere of the brain, contralateral to the right hemisphere in which the GC is infected with the VSV vector (Figure 9e–g). No labeling can be detected in the TTBr, TTBc, and TS on the ipsilateral side.

##### 3.1.2.2 Posterior GC

**FIGURE 11:**
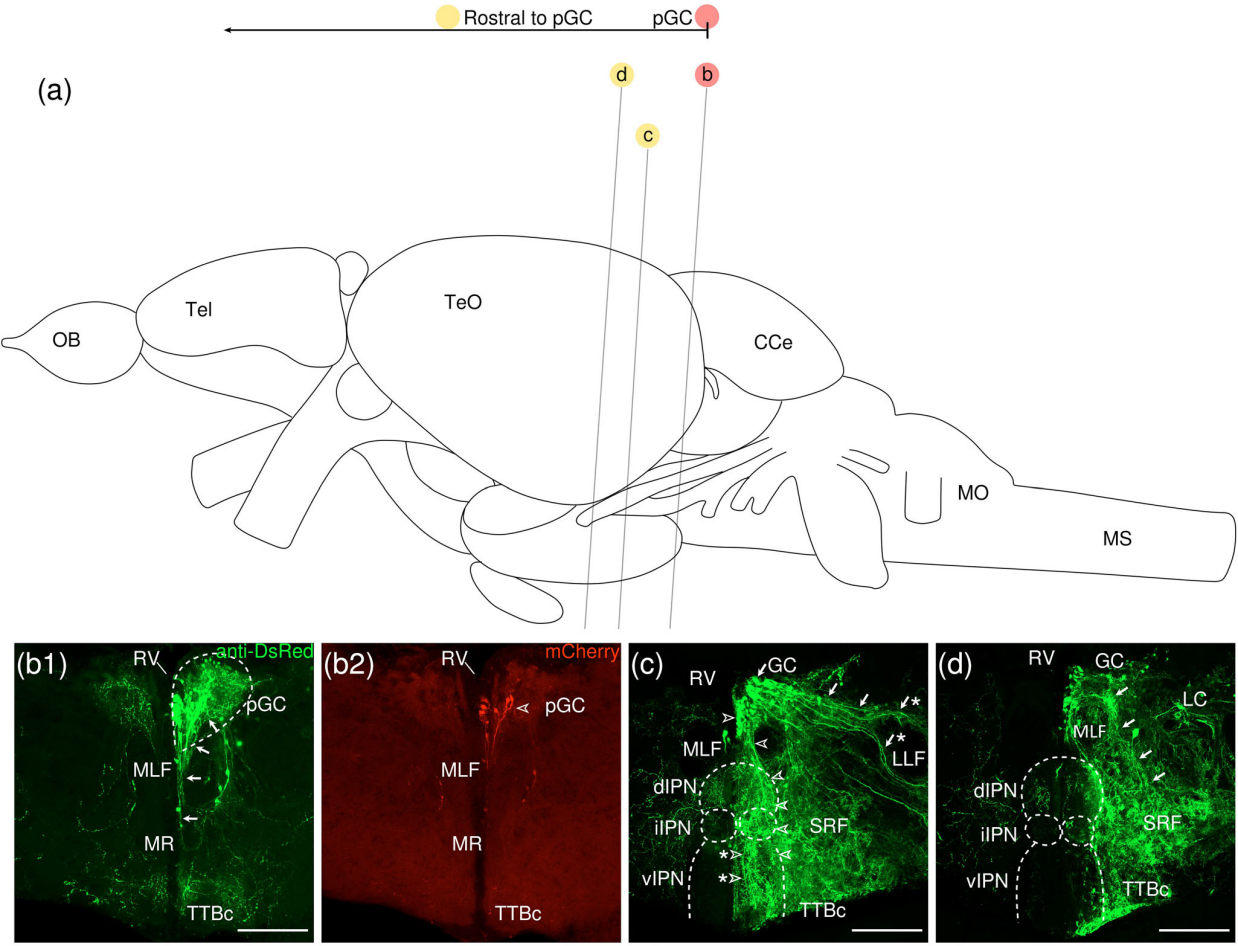
Anterograde trans‐synaptic tracing from the posterior GC with VSV‐mCherry. (a) Schematic diagram of the adult zebrafish brain indicating the level of sections (adapted from Wullimann et al. 1996). Red denotes the injection site at the pGC (b); yellow denotes the regions rostral to the pGC (c, d). (b–d) Coronal sections of the adult zebrafish brain showing labeled areas in the pGC (b) and regions rostral to the pGC (c, d) following injection of trans‐synaptic anterograde tracer VSV‐mCherry in the pGC. Pictures are confocal optical projections of serial cryotome‐cut coronal sections immunostained for mCherry (red; b2) with anti‐DsRed (green; b1, c, d). Figures are arranged in caudorostral order from the pGC. All panels are oriented with dorsal upward. For abbreviations, see the list. (b) Level of the pGC. Dotted outline in b1 shows the pGC. Arrows in b1 point to the somata in the pGC and the associated neurites that extend into the MR, and the arrowhead in (b2) refers to the injection site of VSV‐mCherry at the pGC. (c, d) Level of the IPN. Dotted outline shows the IPN. Arrows in (c) point to the population of GC somata located at the dorsolateral edge of the GC; arrows with asterisk point to the bifurcation of the neurites of the aforementioned somata; arrowheads point to the population of GC somata located at the ventromedial edge of the GC along the midline and the trajectory of the associated neurites along the right lateral border of the dIPN; and arrowheads with asterisk point to the aforementioned neurites turning medially to travel along the midline at the level of the iIPN. Arrows in (d) point to neurites that appear to connect the SRF and the GC. Scale bars = 150 µm.

**FIGURE 12:**
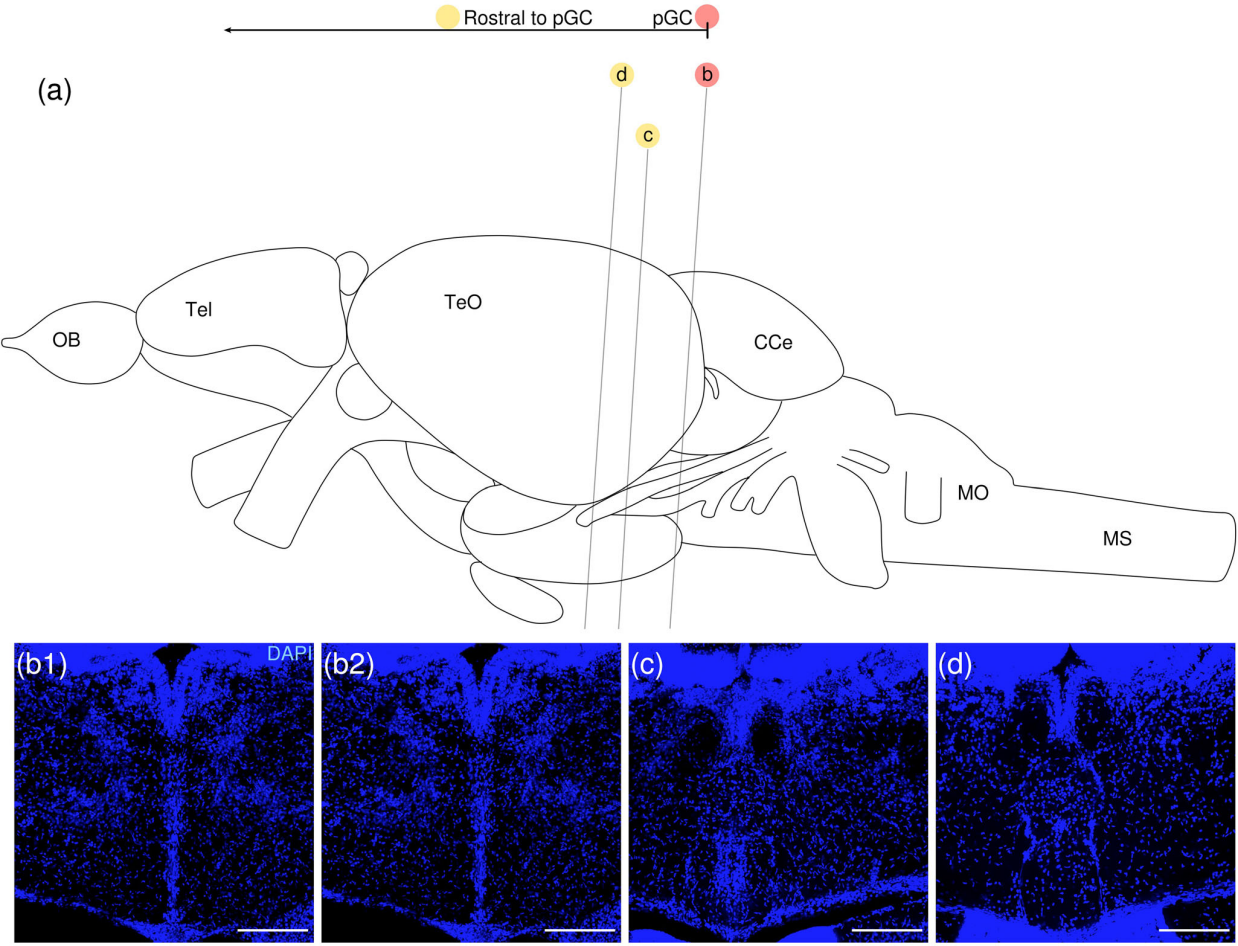
DAPI staining corresponding to Figure 11.

VSV‐mCherry was injected into the right pGC nucleus as a trans‐synaptic anterograde tracer, and one sample was obtained. The injection site can be identified by the small cluster of somata located around the peduncle of the GC nucleus near the midline that expresses high levels of mCherry (Figure 11b, 2, arrowhead; see Figure 12 for counterstaining with the corresponding panel number of Figure 11).

The level of sections is indicated in Figure 11a. Figure 11b–d shows serial sections arranged in caudorostral order from the pGC.

Amplification of the mCherry reporter protein shows labeled neurites bilaterally present in the GC, although the signal is ipsilaterally stronger in the right GC nucleus in which VSV was injected. The VSV‐injected GC nucleus displays a much denser plexus of neurites (Figure 11b, 1), and cell somata close to the midline emit neurites that extend ventrally into the MR nuclei (Figure 11b, 1, arrows).

At the level of the IPN, two distinct populations of somata robustly expressing mCherry are distributed within the right GC nucleus (Figure 11c, arrows and arrowheads). One population (arrows) is located at the dorsolateral edge of the GC nucleus, with neurites traveling diagonally toward the lateral longitudinal fascicle (LLF). Upon encountering the LLF, the fiber bundle bifurcates to go around it (Figure 11c, arrows with asterisk). The other cohort (arrowheads) is organized along the ventromedial edge of the GC near the midline, with neurites extending ventrally toward the IPN and appearing to project along the right lateral border of the dIPN (Figure 11c, arrowheads). However, as the neurites approach the iIPN, these projections turn medially toward the midline and course into the vIPN (Figure 11c, arrowheads with asterisk).

The IPN appears to be split into two halves along the midline, with the left half labeled much more sparsely in comparison to the right. Toward the rostral IPN, projections from the GC stomata predominantly travel along the lateral border of the IPN, forming an outline (Figure 11d). An LC neuron strongly positive for mCherry is present lateral to the VSV‐injected GC nucleus (Figure 11d).

The SRF exhibits moderate mCherry expression and includes a network of thick neurites densely interwoven together (Figure 11d); some of these neurites appear to be connected to the GC (Figure 11d, 1, arrows). The SRF neurites additionally extend to the TTBc area where they aggregate with a plexus of thick neurites (Figure 11c,d).

### 3.2 Retrograde Tracing

Green represents the native fluorescence from the retrograde tracer, and red represents the amplification of the tracer signal by immunohistochemistry staining.

#### 3.2.1 Retrograde Monosynaptic Tracing From d/iIPN

HSV‐GFP was injected to the d/iIPN as a monosynaptic retrograde tracer. One sample was obtained.

##### 3.2.1.1 IPN and Regions Caudal to IPN

**FIGURE 13:**
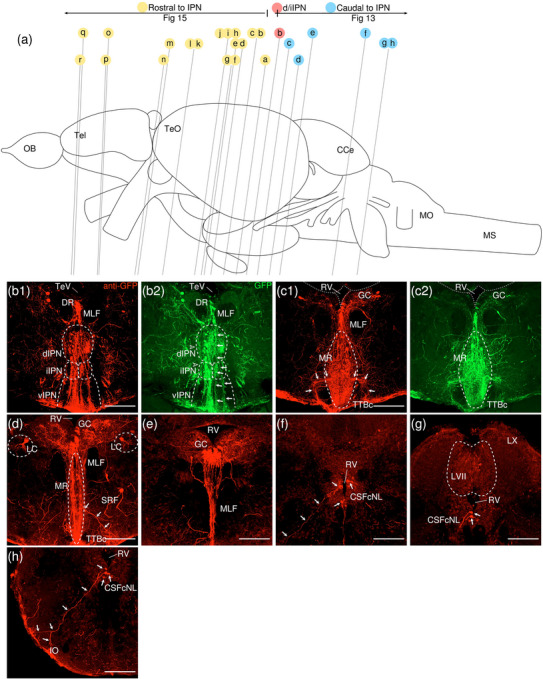
Retrograde monosynaptic tracing from the d/iIPN using HSV‐GFP; regions caudal to IPN. (a) Schematic diagram of the adult zebrafish brain indicating the level of sections (adapted from Wullimann et al. 1996). Red denotes the injection site at the d/iIPN (7b); blue denotes the regions caudal to the IPN (7c‐h); yellow denotes the regions rostral to the IPN (8a–r). (b–h) Coronal sections of the adult zebrafish brain showing labeled areas in the d/iIPN (b) and regions caudal to the IPN (c–h) following injection of the monosynaptic retrograde tracer HSV‐GFP in the d/iIPN. Pictures are confocal optical projections of serial cryotome‐cut coronal sections immunostained for GFP (green; b2, c2) with anti‐GFP (red; b1, c1). Figures are arranged in rostrocaudal order from the IPN. All panels are oriented with dorsal upward. For abbreviations, see the list. (b) Level of the IPN. Dotted outline shows the IPN, and arrowheads in (b2) refer to the injection site of HSV‐GFP at the dIPN and the iIPN. Somata expressing GFP in the IPN are localized within the dorsal and intermediate subnuclei (arrowheads), and several emit fine processes that continue into the vIPN (arrows). (c, d) Level of the MR and GC. Dotted outline in (c, d) shows the MR (center) and in (d) shows the LC (left and right). Arrows point to neurites exiting the rostral MR to extend into the TTBc area. (e) Level of the caudal GC showing neurites forming an inverted bird cage‐like structure. (f) Arrows point to the fine, long‐projecting neurites of the CSF‐cNLs lining the RV. (g, h) Level of the LVII. Arrows point to the CSF‐cNLs and the associated neurites entering the IO. Scale bars = 150 µm.

**FIGURE 14:**
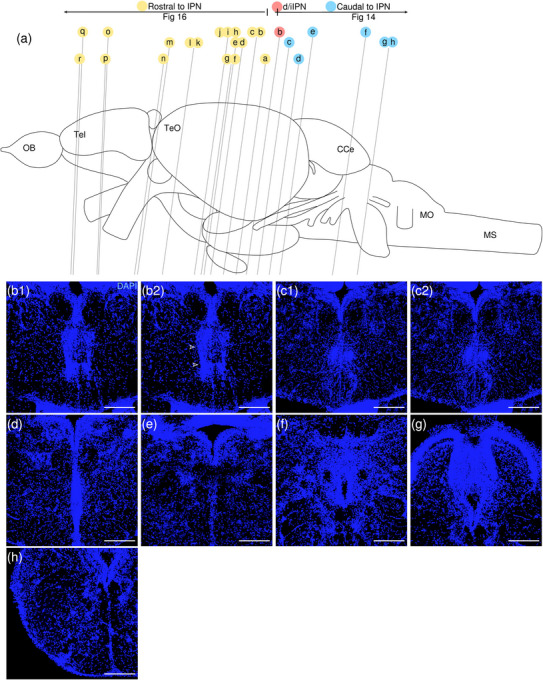
DAPI staining corresponding to Figure 13.

The levels of sections are indicated in Figure 13a. Figure 13b–h shows serial sections arranged in rostrocaudal order from the IPN to the MS (see Figure 14 for counterstaining with the corresponding panel number of Figure 13).

###### 3.2.1.1.1 IPN

Somata expressing GFP in the IPN are localized within the dorsal and intermediate subnuclei (Figure 13b, 2, arrowheads); several emit fine processes that continue into the vIPN (Figure 13b, arrows). Flanked by the MLF, the DR on top of the dIPN contains somata that predominantly gather at the dorsal apex and send neurites into the IPN.

###### 3.2.1.1.2 Rostral Rhombencephalon

The MR expresses GFP robustly throughout (Figure 13c). In the rostral MR, somata show a localization preference to the dorsal aspect of the nucleus, with the ventral half predominantly consisting of a dense plexus. Some neurites exit the MR at approximately midway, where they travel outward horizontally before taking an abrupt turn at almost 90 degrees to extend ventromedially into the TTBc area (Figure 13c, 1, arrows).

At the caudal MR, the nucleus becomes narrower and contains neurites interdigitated with neurons (Figure 13d). Neurites in the MR appear to fasciculate and run dorsoventrally in a vertical orientation, straddling the midline column. Toward the ventral aspect of the MR, some fibers exit the main bundle and ramify extensively into the TTBc (Figure 13d, arrows). The SRF flanks the MR on both sides as a neuropil composed of fine processes and the large, irregularly shaped neurons characteristic of the reticular formation (Figure 13d).

At the level of the MR, most GC neurons lie on the dorsolateral surface of the nucleus (Figure 13c,d). Toward the caudal end of the GC, somata aggregate at the midline, as seen in the results of the anterograde trans‐synaptic tracing previously described. Caudal GC somata emit long neurites that extend ventrally to form an inverted bird cage‐like structure at the level of the MLF before converging into a moderately thick bundle of intertwined fibers (Figure 13e). Likewise with the caudal MR, a cohort of neurites exit the main tract and branch into the TTBc (Figure 13d, arrows). The large, distinct cells of the LC that are lateral to the GC on both sides of the brain are brightly labeled (Figure 13d).

In the hindbrain, somata lining the walls of RV are provisionally termed cerebrospinal fluid (CSF)‐contacting neuron‐like cells (CSF‐cNLs) due to their characteristic localization at the ventricular walls and their fine, long‐projecting neurites (Figure 13f, arrows) (see Section 4).

###### 3.2.1.1.3 Caudal Rhombencephalon and MS

The facial lobe (LVII) contains small cell bodies that aggregate close to its center (Figure 13g).

Several CSF‐cNLs are found abutting the ventral pole of the RV in the brainstem (Figure 13g, arrows, h, arrows). As with those located more rostrally (Figure 13f, arrows), they emit distinctly long neurites that transverse from the ventricular perimeter to the border of the brain. These CSF‐cNL neurites appear to converge into a single strand shortly after emerging, which then travel radially across the ventral aspect of the brain before arborizing to enter the inferior olive (IO) at the ventral border (Figure 13h, arrows).

##### 3.2.1.2 Regions Rostral to the IPN

###### 3.2.1.2.1 Mesencephalon

**FIGURE 15:**
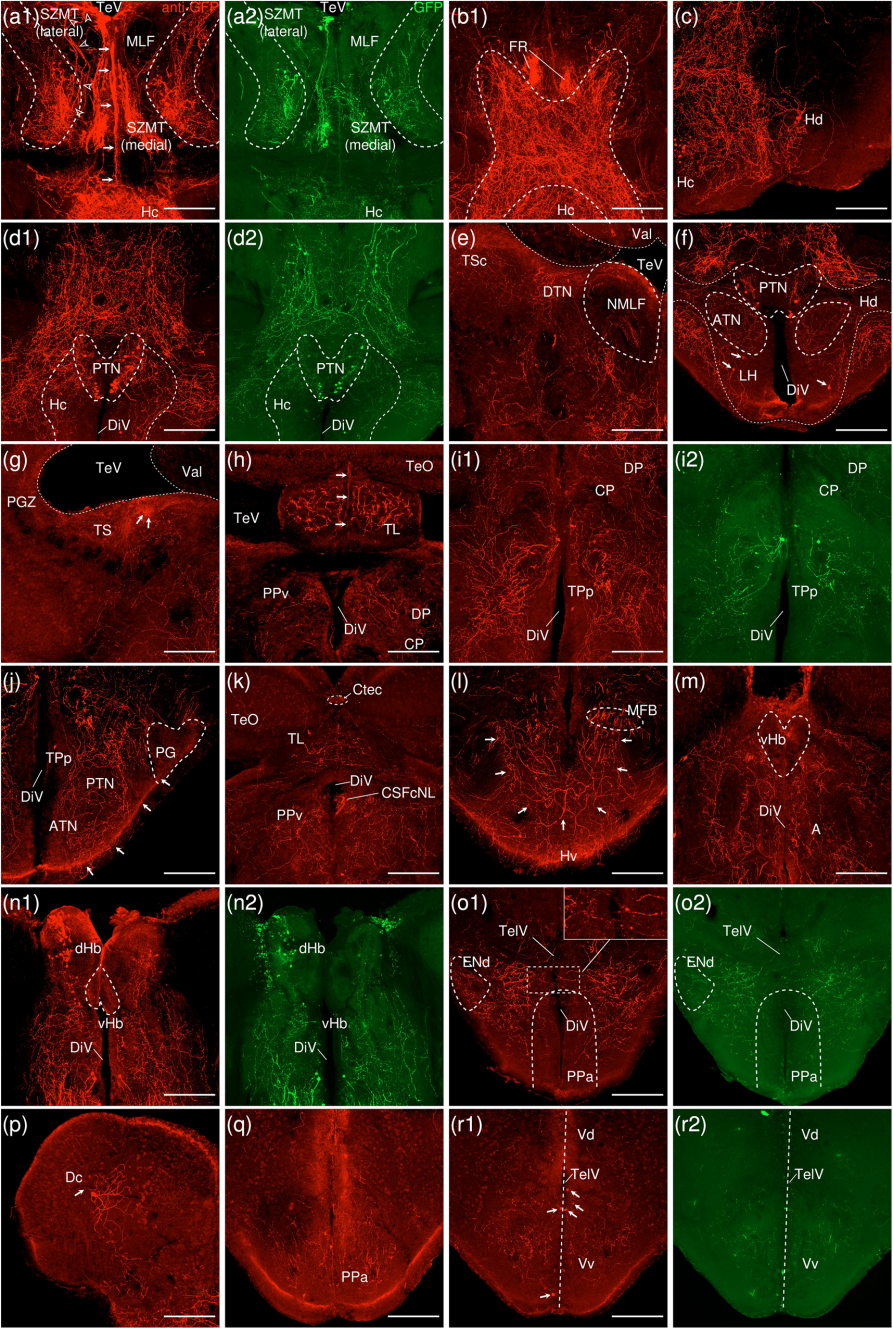
Retrograde monosynaptic tracing from the d/iIPN using HSV‐GFP; regions rostral to IPN. (a–r) Coronal sections of the adult zebrafish brain showing labeled regions rostral to the IPN following injection of monosynaptic retrograde tracer HSV‐GFP in the d/iIPN. Pictures are confocal optical projections of serial cryotome‐cut coronal sections immunostained for GFP (green) with anti‐GFP (red). Figures are arranged in caudorostral order from the IPN. All panels are oriented with dorsal upward. For abbreviations, see the list. (a) Level of the SZMT. Dotted outlines show the bilateral, boomerang‐shaped plexuses that laterally flank the medial SZMT. Arrows point to the diagonally oriented cohort of medial SZMT neurites that descend to the Hc. Arrowheads point to the other cohort of medial SZMT neurites that can be traced to the neurons in the lateral SZMT. (b, c) Dotted outline in (b) shows the butterfly‐shaped neuropil dorsal to the Hc. Some collaterals of the neuropil extend into the Hc and the Hd. (d) Dotted outlines show the PTN (top) and the Hc (bottom). Somata in the PTN appear to emit neurites dorsally into the caudal aspect of the butterfly‐shaped neuropil. (e) Level of the DTN and TSc. Dotted outline shows NMLF. (f) Level of the tuberal nucleus (PTN and ATN) at the ventral aspect of the brain. Arrows point to somata in the LH. (g) Arrows point to somata in the TSc emitting neurites that arborize into a plexus. (h) Level of the TL showing varicosity‐bearing neurites covering the entirety of the region. Arrows point to a tract of neurites running through the center of the TL. (i) Level of the DP, CP, and TPp. (j) Dotted outline shows PG. Arrows point to a bundle of neurites running along the ventralmost border of the brain to the level of the PG. (k) Dotted outline shows Ctec. (l) Dotted outline shows MFB. Arrows point to a plexus of neurites roughly arranged in a U‐shape in an unknown region dorsal. (m) Level of the vHb and A, showing the irregularly shaped neurites in A running vertically. (n) Level of the Hb, showing labeled somata in both the dHb and the vHb. The dHb exhibits a preferentially ipsilateral distribution of somata to the left dHb. (o) Dotted outlines show the ENd (left) and the PPa (center bottom). Inset shows neurites from the two hemispheres meeting at the midline in the PPa. (p) Level of the Dc. Arrow points to a single, large somata in the Dc emitting multiple thick neurites that arborize extensively. (q) Vertically oriented neurites arranged roughly in parallel and several small somata in the PPa. (r) Level of the ventral telencephalic area. Dotted line shows the midline. Arrows point to stomata at the border of the TeV along the midline. Scale bars = 150 µm.

**FIGURE 16:**
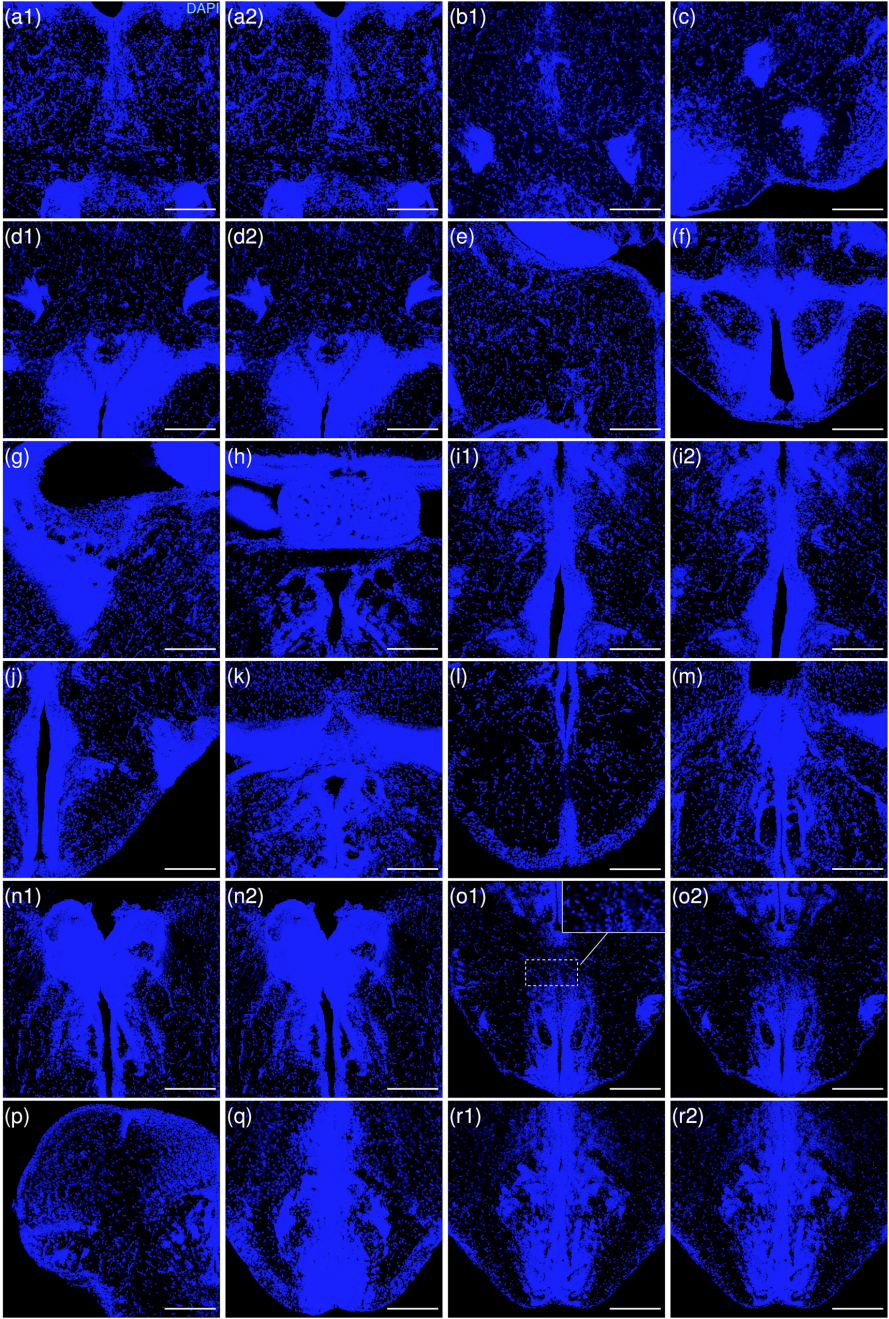
DAPI staining corresponding to Figure 15.

The lateral SZMT consists of a layer of large somata lining the ventral wall of the telencephalic ventricle (TeV) and is strongly positive for GFP (Figure 15a; see Figure 16 for counterstaining with the corresponding panel number of Figure 15). Somata in the lateral SZMT give rise to neurites that travel close to the midline, and they extend vertically into the medial SZMT as a coherent arrangement of neurites. In comparison to the lateral SZMT, GFP expression in the medial SZMT is noticeably weaker (Figure 15a, 2). Another subpopulation of somata in the lateral SZMT gives rise to neurites that arc around the outer perimeter of the MLF and enter the bilateral, boomerang‐shaped plexuses that laterally flank the medial SZMT (Figure 15a). These plexuses consist of densely packed neurites interspersed with neurons and are robustly positive for GFP (Figure 15a, 2).

Based on neurite projection trajectory, localization, GFP expression, and morphology, the medial SZMT appears to contain two distinct cohorts of neurites—one is strongly positive for GFP (Figure 15a, 2) and can be directly traced to the neurons in the lateral SZMT. This cohort runs bilaterally in a diagonal orientation and appears to terminate at the level of the boomerang‐shaped plexuses (Figure 15a, 1, arrowheads). The other is vertically oriented and descends along the midline column into the Hc, a dense trapezoid region in the ventral aspect of the brain (Figure 15a, 1, arrows).

###### 3.2.1.2.2 Diencephalon

The conspicuously butterfly‐shaped neuropil that lies dorsal to the Hc is one of the most heavily labeled structures in the mesencephalon (Figure 15b, outlined). As the neuropil approaches the ventral floor of the brain, some collaterals extend into the Hc and the dorsal zone of the periventricular hypothalamus (Hd) (Figure 15b,c). The FR can be delineated from this neuropil due to its more compact arrangement of fibers forming two dense, distinct bundles (Figure 15b).

The caudal part of the PTN contains GFP‐expressing cell bodies, which predominantly cluster at its ventral pole (Figure 15d). These somata appear to emit neurites dorsally into the caudal aspect of the butterfly‐shaped plexus. At this level, the plexus maintains its morphology as a densely packed plexus of tightly intertwined neurites and a smattering of round somata. Some neurites from the PTN extend into the surrounding Hc but appear to be mostly confined to the dorsal aspect of the Hc and gradually dissipate toward the ventral floor of the brain (Figure 15d).

In the ventral aspect of the brain, the majority of cell bodies are contained in the PTN, close to the boundary of the anterior tuberal nucleus (ATN) (Figure 15f). Somata in the PTN extend neurites that ramify into the neuropil in the Hd surrounding the lateral recess of the diencephalic ventricle (LR). Some labeled cell bodies are present in the lateral hypothalamic nucleus (LH) (Figure 15f, arrows).

The nucleus of MLF (NMLF), DTN, and TSc are neighboring structures that are contiguous with each other, but the plexuses of fibers in each of the regions do not appear to be interconnected (Figure 15e). The TSc, a subdivision of the torus semicircularis (TS), contains several somata (Figure 15g, arrows) emitting neurites that arborize into a meshwork of neurites.

The entirety of the torus longitudinalis (TL) is covered with varicosity‐bearing neurites with irregular diameters, as it extends longitudinally (Figure 15h,k). A thin fibrous tract runs through the center of the nucleus (Figure 15h, arrows).

The ventral part of the periventricular pretectal nucleus (PPv), the dorsal posterior thalamic nucleus (DP), and the CP are situated along the ventricular walls of the DiV, which runs along the midline. Neurites emerge from the PPv at the dorsalmost aspect of the DiV and travel diagonally toward the DP (Figure 15h).

Neurites in the CP extend into the TPp, where several somata expressing GFP are found along the midline (Figure 15i). Fibers in the TPp ramify and spread outward ventrolaterally in a diagonally descending pattern, forming a moderately dense plexus within both hemispheres before diving further into the ATN (Figure 15i,j).

A bundle of long neurites runs along the ventralmost border of the brain to the level of the preglomerular nucleus (PG) (Figure 15j, arrows). These neurites also run through the ATN, but it is difficult to determine if the PG and ATN are connected.

The commissura tecti (Ctec) is embedded with several somata (Figure 15k).

Near the dorsal pole of the DiV, the region is lined with several somata emitting thick neurites that appear to extend ventrolaterally along the subcommissural organ (Figure 15k); these somata are presumed to be CSF‐cNLs. Ventral to the CSF‐cNLs, the PPv contains a loosely organized plexus on both sides of the DiV (Figure 15k).

The ventral aspect of the brain contains a plexus of neurites roughly arranged in a U‐shape in an unknown region dorsal to the ventral zone of the periventricular hypothalamus (Hv) (Figure 15l, arrows). The MFB manifests as layer of particularly short neurites lying on top of the U‐shaped plexus.

The neurites in the anterior thalamic nucleus (A) take on an irregular appearance and run in a vertical orientation (Figure 15m).

The Hb contains neurons intensely positive for GFP (Figure 15n, 2). In the dorsal subdivision of the Hb, the population distribution pattern of somata exhibits an ipsilateral preference to the left dHb, which contains two clusters of large round somata robustly positive for GFP (Figure 15n). The right dHb consists predominantly of neurites, and the few somata that are present express GFP much less intensely. In the vHb, several somata very weakly expressing GFP are present on the left, but the right vHb contains only neurites (Figure 15n). In comparison to the somata in the dHb, vHb somata are markedly smaller in size.

###### 3.2.1.2.3 Telencephalon

The dorsal part of the entopeduncular nucleus (ENd) contains a small plexus of GFP‐expressing neurites within both hemispheres (Figure 15o, 2), a portion of which dives ventrally into the PPa (Figure 15o). The other portion of the plexus extends medially and thickens as it approaches the unknown region between the ENd and PPa, before thinning out upon encountering the PPa. The two cohorts of neurites from the two hemispheres meet at the midline in the PPa (Figure 15o, inset).

The central zone of the dorsal telencephalic area (Dc) contains a single, large GFP‐positive neuronal cell body emitting multiple thick neurites (likely to be dendrites) that arborize extensively in the medial direction (Figure 15p, arrow). However, the primary collaterals appear to be enclosed within the confines of the Dc and do not penetrate the other subregions of the dorsal telencephalic area in the immediate vicinity.

The rostral PPa contains vertically oriented fibers arranged in a roughly parallel pattern, and in its ventral aspect, it contains several small somata (Figure 15q).

The dorsal nucleus of the ventral telencephalic area (Vd) is innervated very faintly only by a few fine fibers, but the ventral nucleus of the ventral telencephalic area (Vv) underneath is moderately positive for GFP (Figure 15r). The few somata labeled in the ventral telencephalic area are localized at the border of the TeV along the midline (Figure 15r, 1, arrows).

#### 3.2.2 Retrograde Trans‐Synaptic Tracing From d/iIPN

VSV(RABV‐G)‐GFP was injected to the d/iIPN as a trans‐synaptic retrograde tracer. One sample was obtained.

**FIGURE 17:**
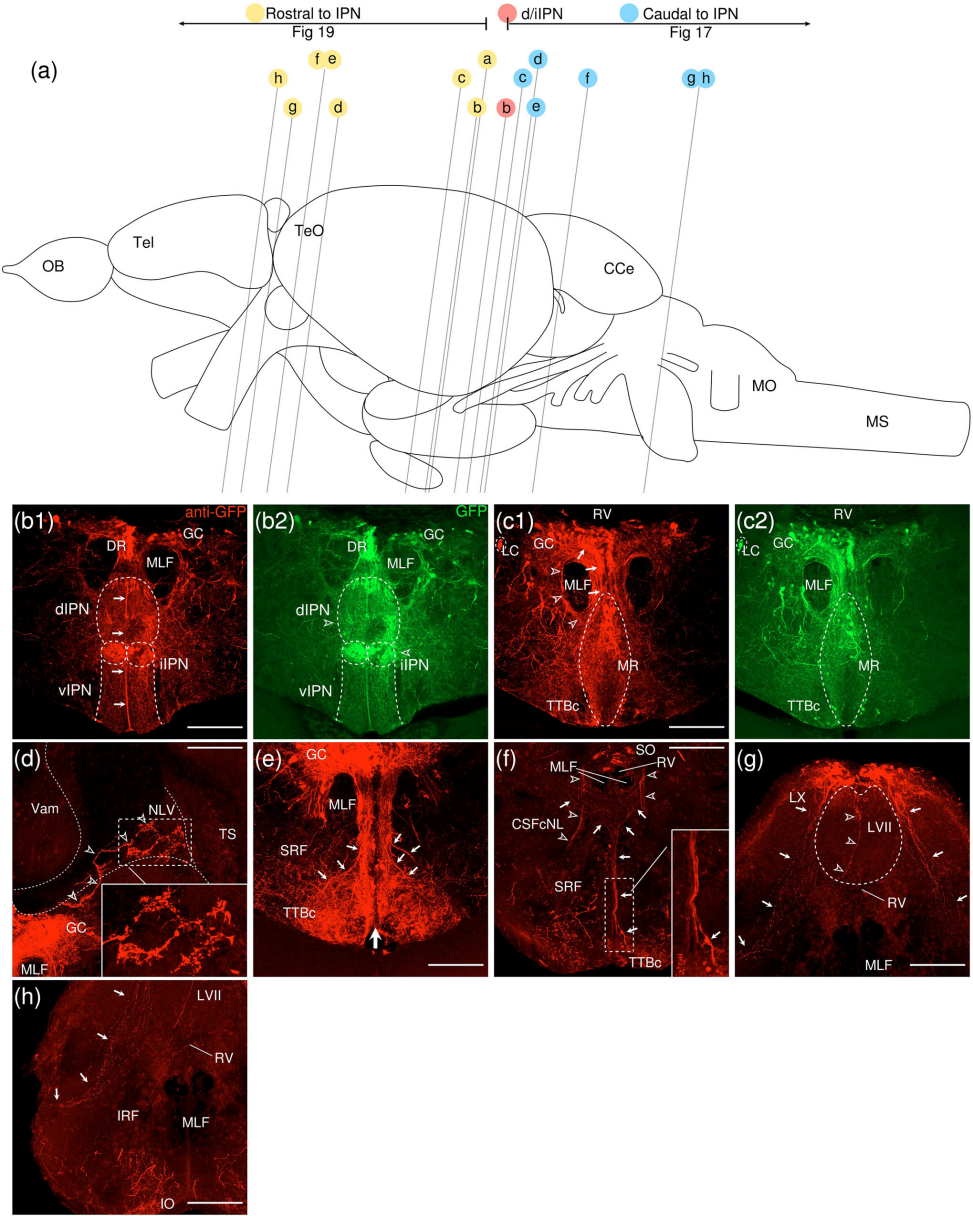
Retrograde trans‐synaptic tracing from the d/iIPN using VSV(RABV‐G)‐GFP; regions caudal to IPN. (a) Schematic diagram of the adult zebrafish brain indicating the level of sections (adapted from Wullimann et al. 1996). Red denotes the injection site at the d/iIPN (9b1); blue denotes the regions caudal to the IPN (9c–h); yellow denotes the regions rostral to the IPN (10a–h). (b–h) Coronal sections of the adult zebrafish brain showing labeled areas in the d/iIPN (b) and regions caudal to the IPN (c–h) following injection of the trans‐synaptic retrograde tracer VSV(RABV‐G)‐GFP in the d/iIPN. Pictures are confocal optical projections of serial cryotome‐cut coronal sections immunostained for GFP (green) with anti‐GFP (red). Figures are arranged in rostrocaudal order from the IPN. All panels are oriented with dorsal upward. For abbreviations, see the list. (b) Level of the IPN. Dotted outline shows the IPN, and arrowheads in (b2) refer to the injection site of VSV(RABV‐G)‐GFP at the dIPN and the iIPN. (c) Level of the MR and GC. Dotted outlines show the MR (center) and the LC (top left). Arrows point to the neurites of GC somata extending into the MR, and arrowheads point to a smaller subset of neurites from the GC encircling the lateral border of the MLF and looping into the MR. (d) Level of the NLV and GC. Inset shows the irregular morphology of one subpopulation of NLV somata, and arrowheads show the winding trajectory of the NLV neurites. (e) Arrows point to the fibers that diverge from the main branch of the pair of fascicles sandwiched by the MLF to enter the TTBc, and the filled arrow points to the unlabeled gap along the midline. (f) Inset shows the long‐projecting neurite emitted by a large somata lying close to the midline, arrows point to the trajectory of the aforementioned somata, and arrowheads point to CSF‐cNL neurites that gather at the sides of the MLF. (g, h) Dotted outline in (g) shows LVII, and arrowheads in (g) point to a thin fiber emitted by LVII somata that travels dorsolaterally across the nucleus. Arrows point to the neurites emitted by the densely packed somata in the dorsal pole of the LX. Scale bars = 150 µm.

**FIGURE 18:**
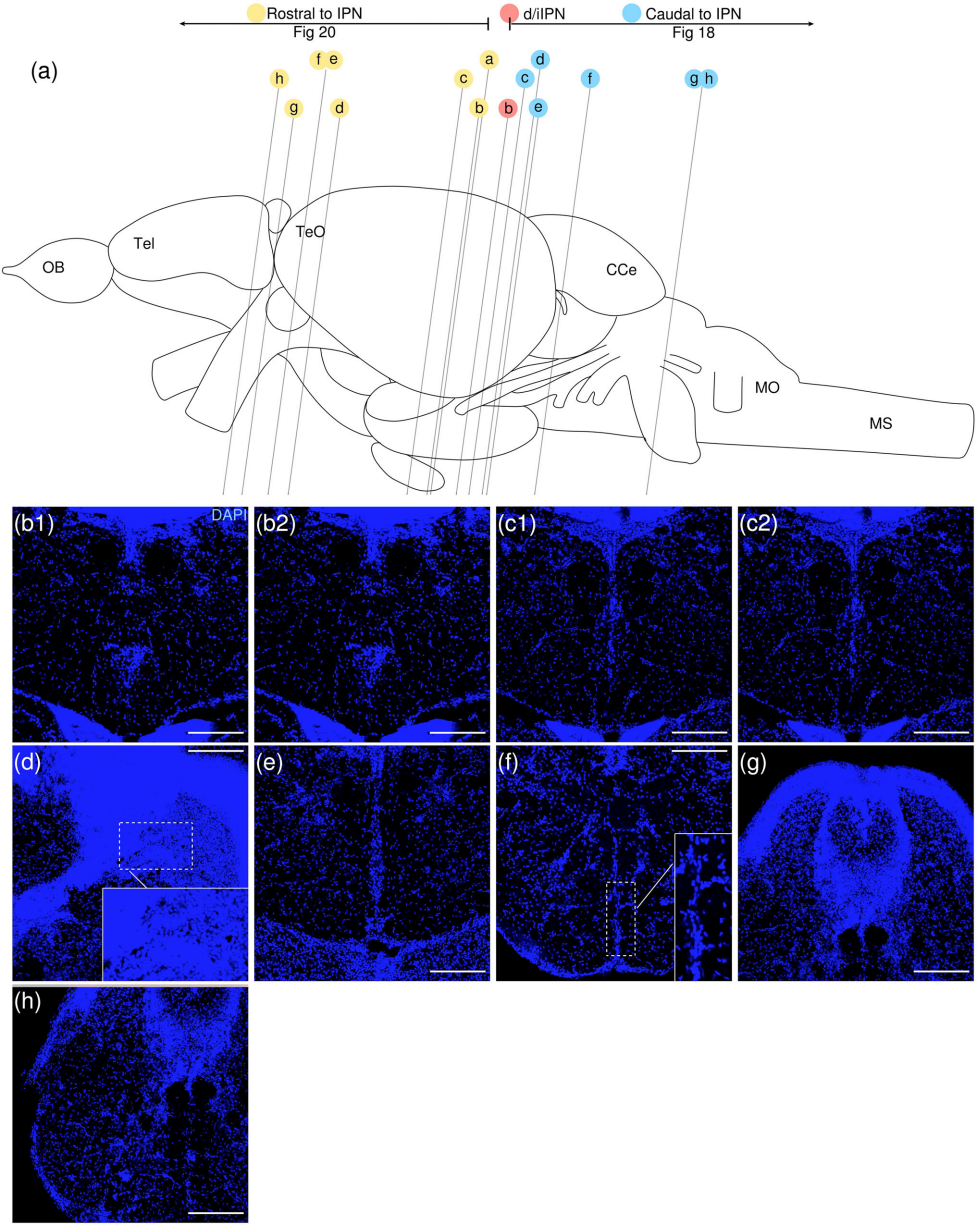
DAPI staining corresponding to Figure 17.

The levels of sections are indicated in Figure 17a. Figure 17b–h shows serial sections arranged in rostrocaudal order from the IPN (see Figure 18 for counterstaining with the corresponding panel number of Figure 17).

##### 3.2.2.1 IPN and Regions Caudal to IPN

###### 3.2.2.1.1 IPN

Somata expressing the GFP reporter protein derived from the viral tracer are localized in the dorsal and intermediate subnuclei of the IPN (Figure 17b), indicating successful delivery of the viral tracer to the target region. Within the dIPN, the somata lie at the dorsalmost boundary of the subnucleus; in the iIPN, most of the labeled somata are situated close to the center. A thin labeled neurite runs along the midline of the IPN, traversing either the dorsal, intermediate, and ventral subnuclei or the septal structure (Figure 17b, arrows). Dorsal to the IPN, cell bodies in the DR aggregate at the dorsal apex of the nucleus and are robustly labeled (Figure 17b).

###### 3.2.2.1.2 Rostral Rhombencephalon

The majority of cell bodies in the GC lie at the dorsolateral perimeter of the nucleus, and their neurites extend into the ventrally located MR (Figure 17c, arrows). A smaller subset of neurites emerging from the GC, however, encircles the lateral border of the MLF and loops into the MR (Figure 17c, arrowheads). The LC situated at the ventrolateral corner of the GC is labeled (Figure 17c) and is robustly positive for GFP (Figure 17c, 2).

The entirety of the MR is covered in a dense neuropil that gradually dissipates toward its ventral pole (Figure 17c). The dorsal aspect is interdigitated with large GFP‐expressing somata that are predominantly clustered in the middle of the nucleus (Figure 17c, 2), while smaller somata line the ventrolateral boundary. The ventral MR is bilaterally flanked by the TTBc, which is punctuated with small varicosities (Figure 17c).

Somata in the NLV appear to take on two different morphologies—one subpopulation is comparatively much larger and is irregularly shaped (Figure 17d, inset), whereas the remainder appear to be small and round. These somata are embedded in a winding plexus of neurites (Figure 17d, arrowheads), some of which extend toward the vicinity of the GC and ramify into fine processes.

Immediately caudal to the MR, GC neurons extend neurites that fasciculate to bilaterally form thick, dense bundles with an unlabeled gap along the midline (Figure 17e, filled arrow). This pair of fascicles is interspersed with large neurons toward the ventral aspect. These neurons project in a dorsoventral orientation, and some neurites diverge from the main branch near the dorsal apex of the TTBc to enter the dense neuropil located there (Figure 17e, arrows). The remainder of neurites continue directly into the TTBc. At this level, somata in the TTBc aggregate near the floor of the region.

The dorsal surface of the RV is dotted with distinct cell bodies associated with long‐projecting processes (Figure 17f). These cells, tentatively termed CSF‐cNLs as described above, are embedded in the ependymal lining of the RV. The CSF‐cNLs send long, vertically projecting neurites that gather into two fine tracts on both sides of the MLF (Figure 17f, arrowheads).

In the TTBc, a large neuron lying close to the midline gives rise to a long‐projecting neurite (Figure 17f, arrows, inset, arrows). It collects into a thin, coherent fascicle that travels along the midline (Figure 17f, arrows). Toward the vicinity of the MLF, the neurites in the tract become finer. The tract then bifurcates to cup the underside of the MLF before dissipating.

###### 3.2.2.1.3 Caudal Rhombencephalon and MS

Toward the posterior hindbrain, the apex of the lobus facialis (LVII) is enveloped by the lobus vagus (LX) on both sides. The dorsal pole of the LX is densely covered with somata emitting long‐projecting fibers that travel in two coherent bundles, flanking the LVII (Figure 17g, arrows). As the fibers traverse the LX, they become finer and more loosely organized. At the level of the MLF, the bundles make a turn toward the lateral edge of the brain (Figure 17g,h, arrows).

Somata in the LVII are much smaller in comparison to those in the LX and congregate around the dorsocentral aspect of the nucleus (Figure 17g). A thin fiber emitted by LVII somata travels dorsolaterally across the nucleus (Figure 17g, arrowheads).

The IO lies at the floor of the brain and appears as a labeled plexus of neurites (Figure 17h).

##### 3.2.2.2 Regions Rostral to IPN

**FIGURE 19:**
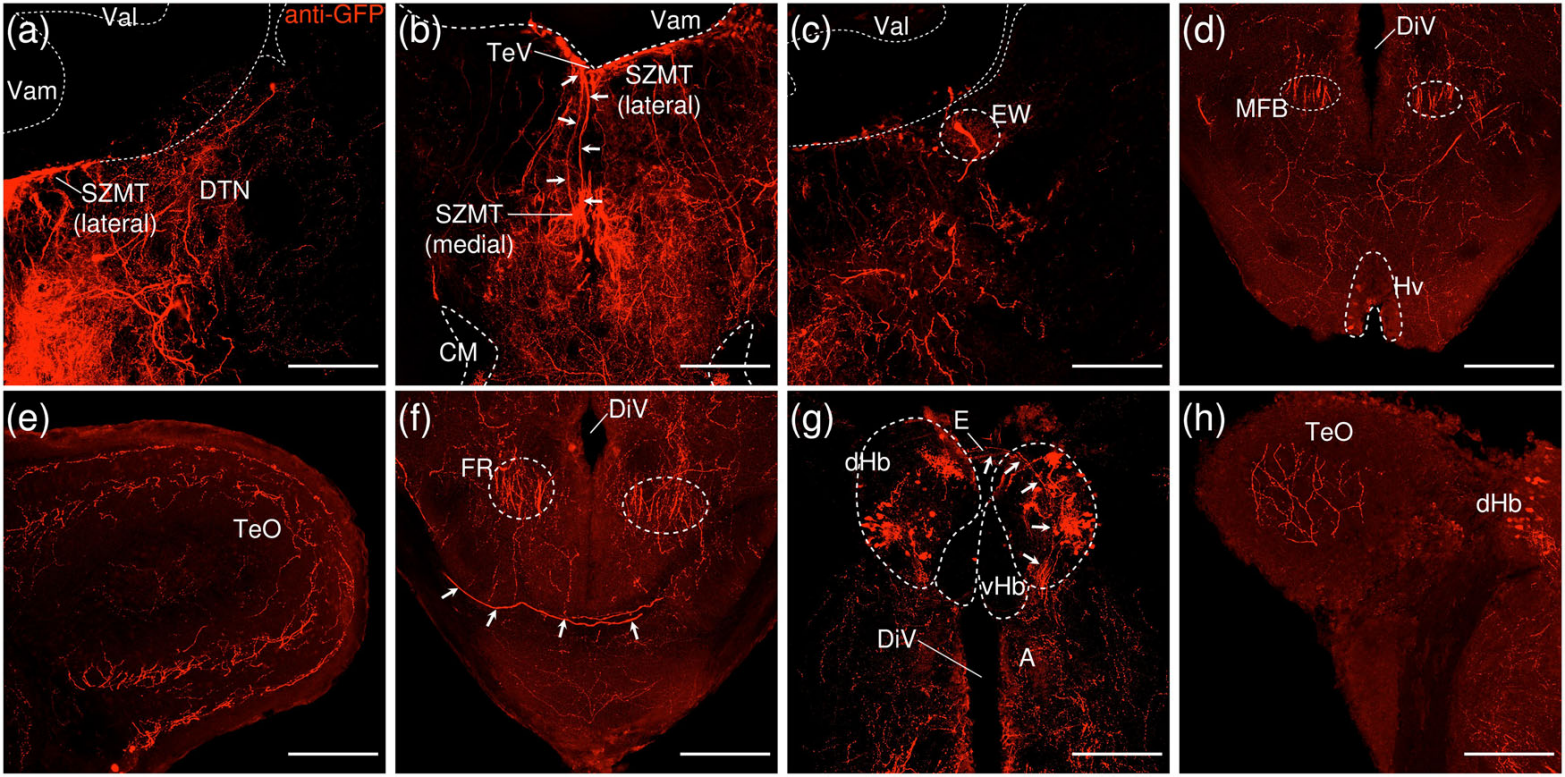
Retrograde trans‐synaptic tracing from the d/iIPN using VSV(RABV‐G)‐GFP; regions rostral to IPN. (a–h) Coronal sections of the adult zebrafish brain sections showing labeled regions rostral to the IPN following injection of trans‐synaptic retrograde tracer VSV(RABV‐G)‐GFP in the d/iIPN. Pictures are confocal optical projections of serial cryotome‐cut coronal sections immunostained for GFP (green) with anti‐GFP (red). Figures are arranged in caudorostral order from the IPN. All panels are oriented with dorsal upward. For abbreviations, see the list. (a) Level of the DTN and the lateral SZMT. (b) Level of the SZMT showing both the lateral and medial subregions. Dotted outlines show the bilateral CM, and arrows show processes from the lateral SZMT running dorsoventrally to enter the medial SZMT. (c) Dotted outline shows the EW. (d) Dotted outlines show the bilateral MFB (top) and the Hv (bottom). (e) Level of the TeO showing the layer organization. (f) Arrows point to the two thick neurites crossing the midline in the ventral region of the diencephalon. (g) Level of the Hb showing the large neurons contained in the dHb. Dotted outlines show the dHb (top) and the vHb (bottom). (h) Level of the rostral TeO and dHb. Scale bars = 150 µm.

**FIGURE 20:**
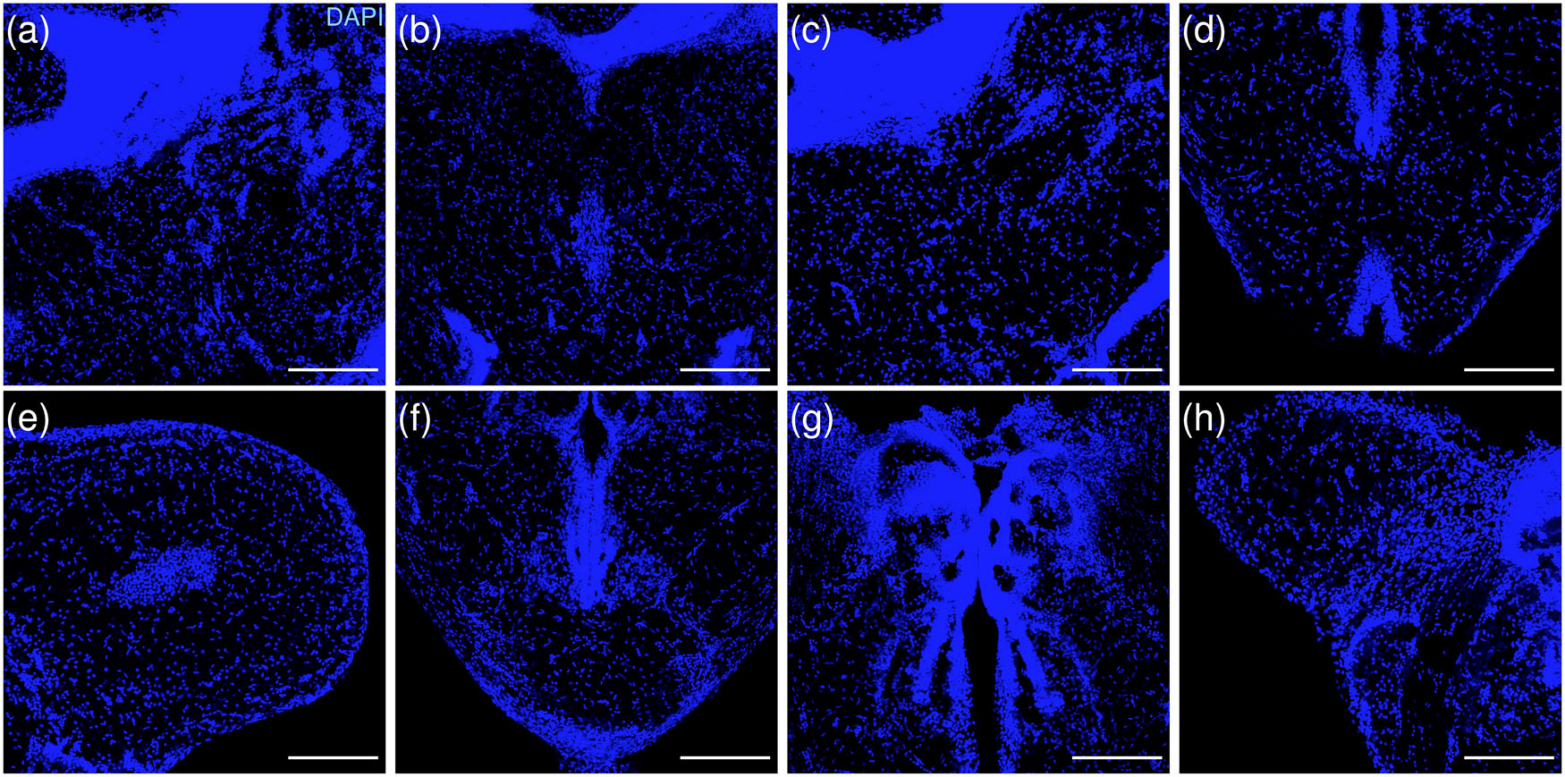
DAPI staining corresponding to Figure 19.

The level of sections is indicated in Figure 17a. Figure 19 shows serial sections arranged in caudorostral order from the IPN to the telencephalon (see Figure 20 for counterstaining with the corresponding panel number of Figure 19).

###### 3.2.2.2.1 Mesencephalon

A plexus of fine neurites reaches the DTN (Figure 19a). As it approaches the vicinity of the lateral SZMT, the diameter of the neurites increases.

The lateral SZMT underlies the ventrolateral surface of the TeV and is composed of a layer of cells arranged along the ventral ventricular walls. These somata send dorsoventrally running processes into the medial SZMT (Figure 19b, arrows) and its immediate vicinity. The medial SZMT can be distinguished from the dense plexus surrounding it due to its comparatively denser and thicker neurites.

A large, distinct neuron in the Edinger–Westphal (EW) nucleus emits a thick, ventrally oriented neurite (Figure 19c).

The TeO is split into multiple layers, but only some of these layers appear to have conspicuous connections with the d/iIPN (Figure 19e). The layers within the TeO are distinct from each other in morphological thickness, and each of them contains a neuropil made up of extensively ramified fibers (Figure 19h).

Although we did not discern between the individual layers of the TeO, judging from the localization of the signal, it is possible that the stratum marginale, stratum fibrosum et griseum superficiale, and stratum album centrale are positively labeled.

###### 3.2.2.2.2 Diencephalon

Toward the ventral pole of the brain, a bilateral, loosely packed bundle of short fibers is arranged in parallel in the MFB (Figure 19d). Labeled somata in the Hv are situated toward the middle of the nucleus. The region between the MFB and Hv, which has not been annotated in the adult zebrafish brain atlas (Wullimann et al. 1996), contains a sparse plexus running through it (Figure 19d).

Two thick fibers cross the midline in the ventral region of the diencephalon (Figure 19f, arrows).

In the Hb, somata in the Hb are confined within the dHb (Figure 19g). Although somata are preferentially located in the medial subregion of the dHb, the dHb contains dense clusters of cell bodies throughout the entirety of the subnuclei in both hemispheres. The vHb appears to be unlabeled. The bundle of fibers that the FR is composed of can be traced into the E connecting the two Hb hemispheres (Figure 19g, arrows).

#### 3.2.3 Retrograde Trans‐Synaptic Tracing From iIPN

##### 3.2.3.1 IPN and Regions Caudal to IPN

VSV(RABV‐G)‐GFP was injected into the iIPN as a trans‐synaptic retrograde tracer. Two samples were obtained from the injection of VSV(RABV‐G)‐GFP into the iIPN, and in both samples, the injection site was restricted to the intermediate subnuclei.

**FIGURE 21:**
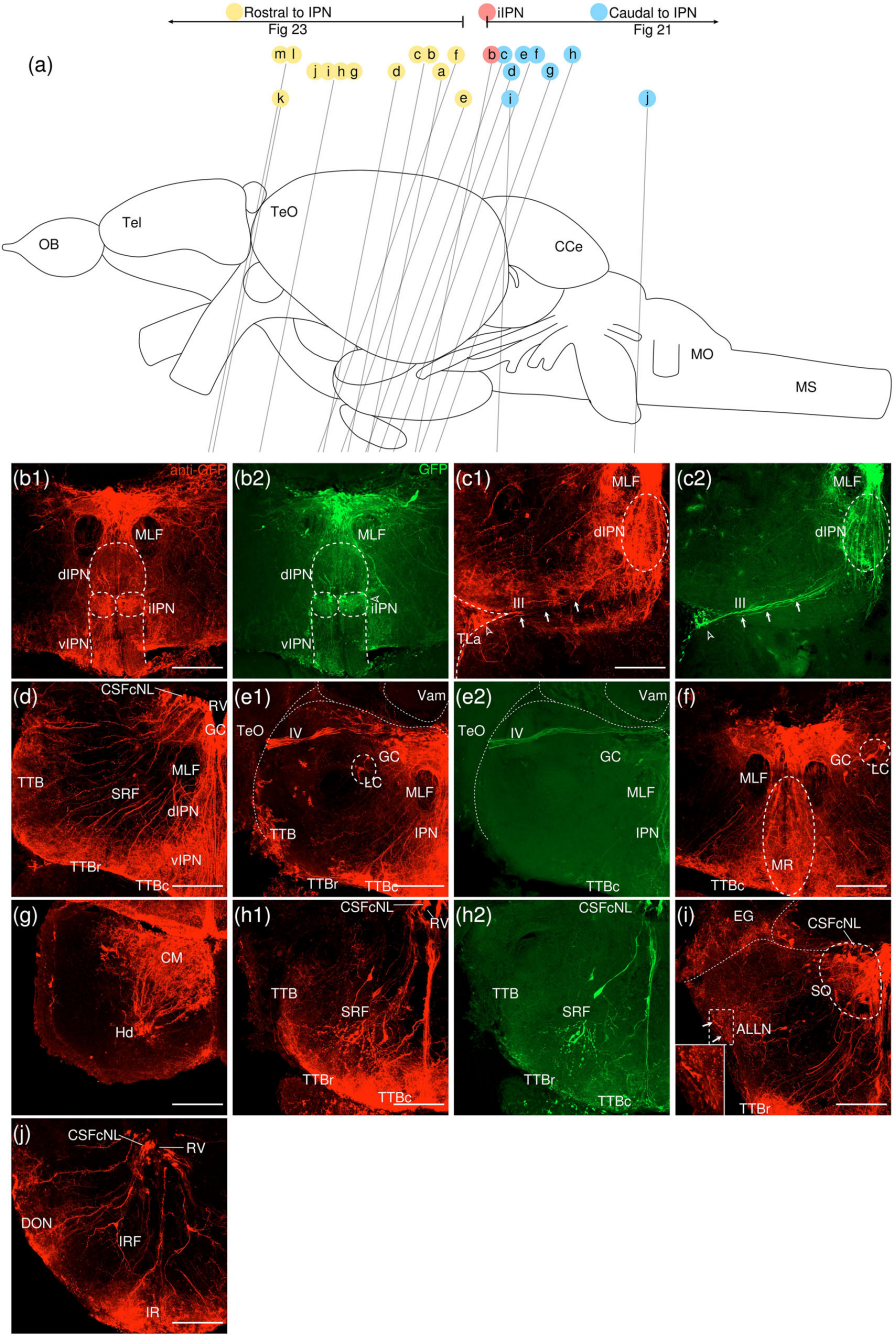
Retrograde trans‐synaptic tracing from the iIPN using VSV(RABV‐G)‐GFP; regions caudal to IPN. (a) Schematic diagram of the adult zebrafish brain indicating the level of sections (adapted from Wullimann et al. 1996). Red denotes the injection site at the iIPN (11b1); blue denotes the regions caudal to the IPN (11c–j); yellow denotes the regions rostral to the IPN (10a–m). (b–i) Coronal sections of the adult zebrafish brain showing labeled areas in the d/iIPN (b) and regions caudal to the IPN (c–j) following injection of the trans‐synaptic retrograde tracer VSV(RABV‐G)‐GFP in the iIPN. Pictures are confocal optical projections of serial cryotome‐cut coronal sections immunostained for GFP (green) with anti‐GFP (red). Figures are arranged in rostrocaudal order from the IPN. All panels are oriented with dorsal upward. For abbreviations, see the list. (b–d) Level of the IPN. Dotted outlines in (b, c) show the IPN; the arrowhead in (b2) refer to the injection site of VSV(RABV‐G)‐GFP at the iIPN; and arrows in (c) point to the III. (d, e) Level of the IPN and GC. (e) Dotted outline shows the LC. (f) Level of the MR and GC. Dotted outlines show the MR (center) and the LC (top right). (g) Level of the CM and Hd at the ventralmost region of the brain showing neurons in the Hd emitting neurites that enter the CM. (h) Level of the SRF, TTB, TTBr, and TTBc showing CSF‐cNLs lining the ventral pole of the RV. (i) Dotted outline shows the SO; inset shows the ALLN; and arrows point to the hallmark morphology of the ALLN as parallel fibers organized in a diagonal pattern. Caudal rhombencephalon and medulla spinalis (j). (j) Level of hindbrain CSF‐cNLs showing CSF‐cNL neurites entering the DON and IR. Scale bars = 150 µm.

**FIGURE 22:**
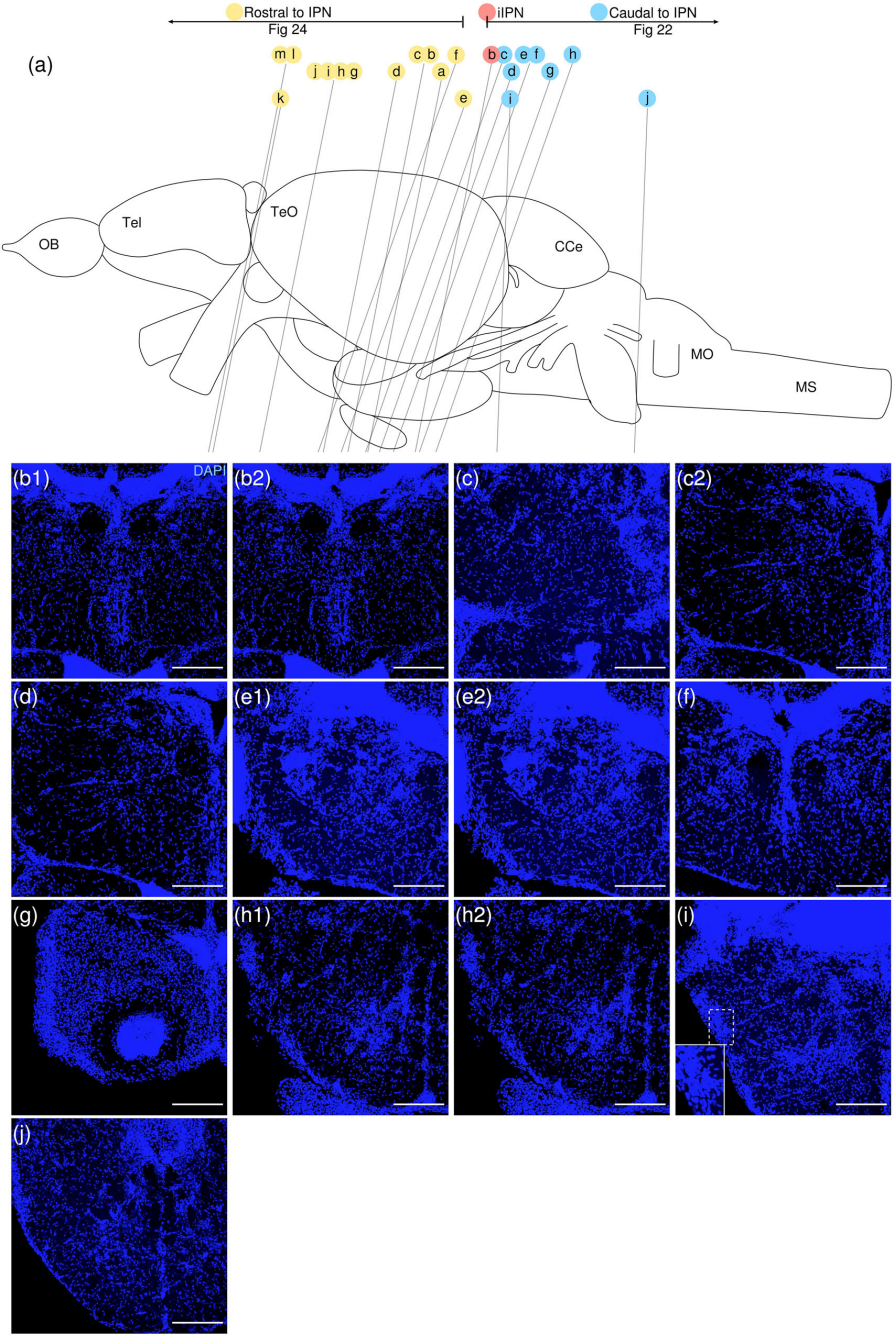
DAPI staining corresponding to Figure 21.

The injection site at the iIPN contains several round somata nestled in the plexus confined within the boundaries of the subnucleus (Figure 21b), indicating that the retrograde viral tracer VSV(RABV‐G)‐GFP was successfully delivered to the target region (see Figure 22 for counterstaining with the corresponding panel number of Figure 21).

In the dIPN, vertically oriented neurites span the entirety of the subnucleus (Figure 21b–d). The vIPN contains a neat arrangement of neurites running parallel to each other throughout the entirety of the subnucleus (Figure 21b,d) and is most likely composed of neurites emitted from the cell bodies in the iIPN (Kinoshita and Okamoto 2023).

The many somata positive for GFP located in the GC are predominantly clustered along the midline column (Figure 21b), which is characteristic of the caudal GC as described above. Some GC processes extend horizontally instead of traveling ventrally into the IPN (Figure 21b).

The oculomotor nerve (III) is robustly positive for GFP (Figure 21c). The fascicle of long, parallel‐running fibers emerges near the ventral periphery of the dIPN and travels toward the medial border of the brain (Figure 21c, arrows). It then arcs ventrolaterally and encounters a small cluster of GFP‐positive cells adjacent to the torus lateralis (TLa) (Figure 21c, arrowhead; Figure 24c).

In the coronal plane, two separate populations of somata are delineable in the area of the GC (Figure 21d). The dorsalmost population lining the lateral walls of the RV is presumed to be CSF‐cNLs on the basis of their location and fine, long‐projecting processes. CSF‐cNL processes radiate into the TTB and TTBr at the border of the brain, passing through the SRF.

Caudal GC neurons concentrate along the midline just beneath the ventral pole of the RV (Figure 21d) and give rise to comparatively thicker neurites that travel vertically through the IPN. A smaller subpopulation of GC neurons emits neurites that go around the MLF before rejoining the main group at the dIPN. Another cohort of GC neurites do not enter the IPN but instead travel along the outer boundaries of the IPN to enter the TTBc directly. At the sides of the GC, the large LC neurons are clearly labeled (Figure 21e,f).

The trochlear nerve (IV) runs longitudinally beneath the cerebellum and spans the length of the coronal plane (Figure 21e). Nerves can be distinguished from other fascicles in the brain on the basis of their distinct morphology as long, thick fibers arranged in parallel to each other. The IV nerve is conspicuously positive for GFP without amplification with IHC staining (Figure 21e).

The MR takes on a granular appearance, and the plexus contained inside the MR shows extensive ramification (Figure 21f). Neurites from the GC run ventrally into the MR as with the IPN, but some part ways with the main cohort and veer off sideways at roughly midpoint to enter the TTBc at its lateral aspect instead.

The TTB runs along the lateral border of the brain as a swath of coarsely packed neuropil (Figure 21d,e).

The Hd contains a moderate cluster of neurons (Figure 21g). These Hd neurons emit radially traversing neurites that enter the corpus mamillare (CM), where they appear to ramify extensively to form a compact plexus. The CM itself also contains multiple small neurons.

CSF‐cNLs lining the ventral pole of the RV give rise to a densely packed fascicle that runs along the midline column and into the TTBc where it arborizes extensively (Figure 21h). A few neurites peel off from the main fascicle at approximately halfway to join the neuropil in the SRF. A separate cohort of CSF‐cNL neurites does not fasciculate but instead arcs ventrally to enter the SRF directly (Figure 21h).

The SRF is robustly positive for GFP and can be easily identified according to its distinctively large, irregularly shaped neurons (Figure 21h). It appears to be closely associated with the TTBc and TTBr.

The majority of somata in the SO aggregate at its dorsal surface, with the ventral aspect consisting mostly of a dense meshwork of neurites (Figure 21i). A few fine neurites emerging from the SO can be traced into the area of the ALLN at the lateral border of the brain. In this coronal section, the ALLN neuropil predominantly consists of puncta. Nevertheless, the hallmark morphology of the ALLN, previously described as parallel fibers organized in a compact diagonal pattern, is visible at the boundary (Figure 21i, inset, arrows).

###### 3.2.3.1.1 Caudal Rhombencephalon and MS

Further caudally into the hindbrain, the GFP‐expressing CSF‐cNLs continue to be embedded in the ependyma of the ventricle (Figure 21j). CSF‐cNL neurites at this level maintain their characteristic fineness and long‐projecting properties but take on a jagged appearance. A thin tract of CSF‐cNL neurites travel along the midline before ramifying to enter the moderately GFP‐positive IR; another cohort of CSF‐cNL neurites project radially to enter the DON through the IRF.

##### 3.2.3.2 Regions Rostral to IPN

**FIGURE 23:**
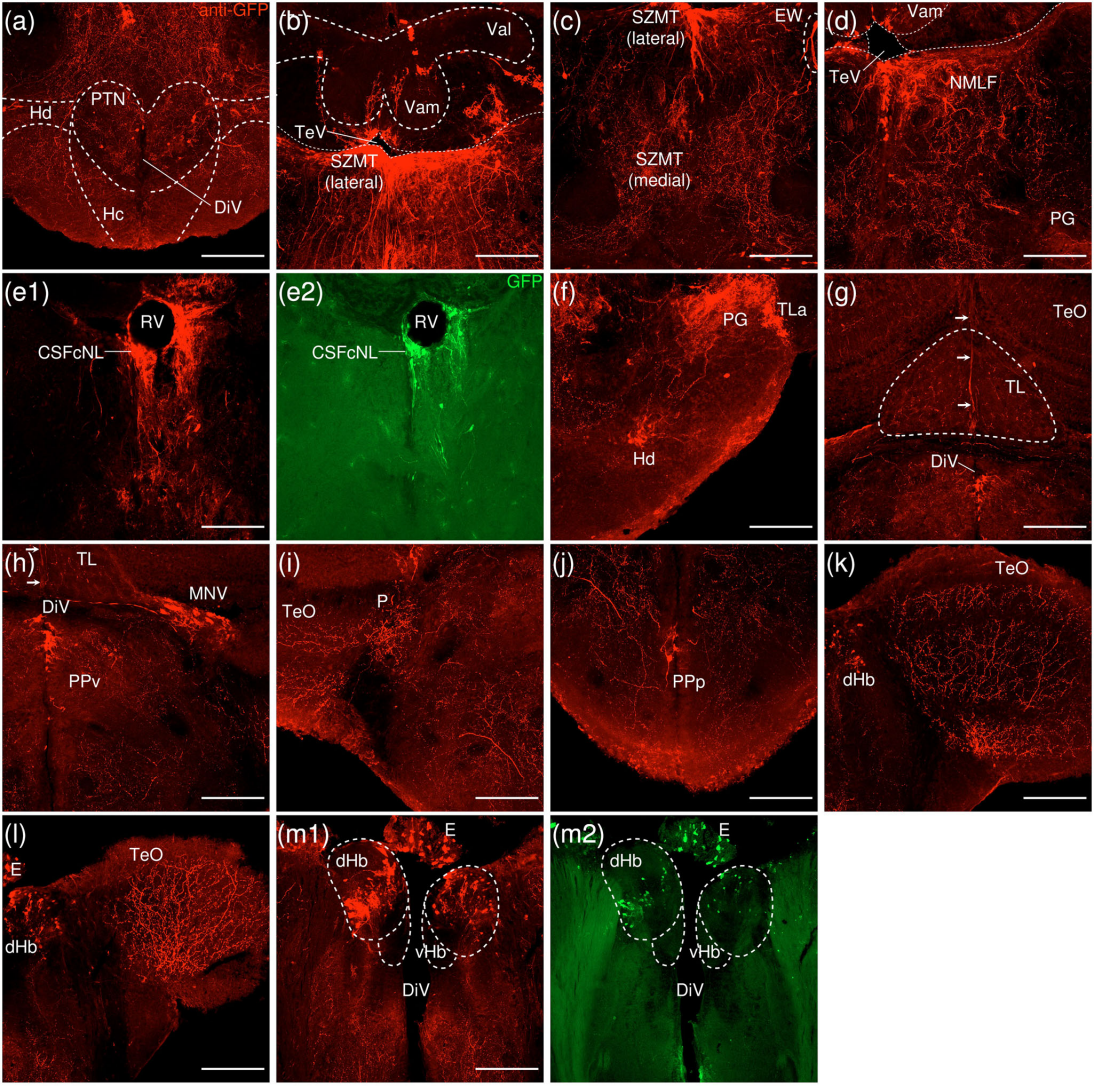
Retrograde trans‐synaptic tracing from the iIPN using VSV(RABV‐G)‐GFP; regions rostral to IPN. (a–h) Coronal sections of the adult zebrafish brain showing labeled regions rostral to the IPN following injection of trans‐synaptic retrograde tracer VSV(RABV‐G)‐GFP in the iIPN. Pictures are confocal optical projections of serial cryotome‐cut coronal sections immunostained for GFP (green) with anti‐GFP (red). Figures are arranged in caudorostral order from the IPN. All panels are oriented with dorsal upward. For abbreviations, see the list. (a) Level of the periventricular hypothalamus showing the Hd and Hc. (b) Level of the cerebellum and SZMT. Dotted outline shows the Val and Vam. (c) Level of the SZMT and EW. Dotted outline shows the EW. (d) Level of the TeV. (e) CSF‐cNLs lining the ventral perimeter of the RV. (f) Level of the PG and Hd. (g) Dotted outline shows the TL, and arrows point to the fibrous tract running along the midline of the TL. (h) Somata in the MNV and PPv. (l) Plexus in P appears to be contiguous with the TeO neuropil. (j) Somata in the PPp near the midline. (k, l) Level of the TeO showing the distinct layers in the TeO; image (l) shows a more rostral region of the TeO. (m) Level of the Hb showing the large neurons contained in the dHb, with neuronal distribution showing an ipsilateral preference to the left dHb. Dotted outlines show the dHb (top) and the vHb (bottom). Scale bars = 150 µm.

**FIGURE 24:**
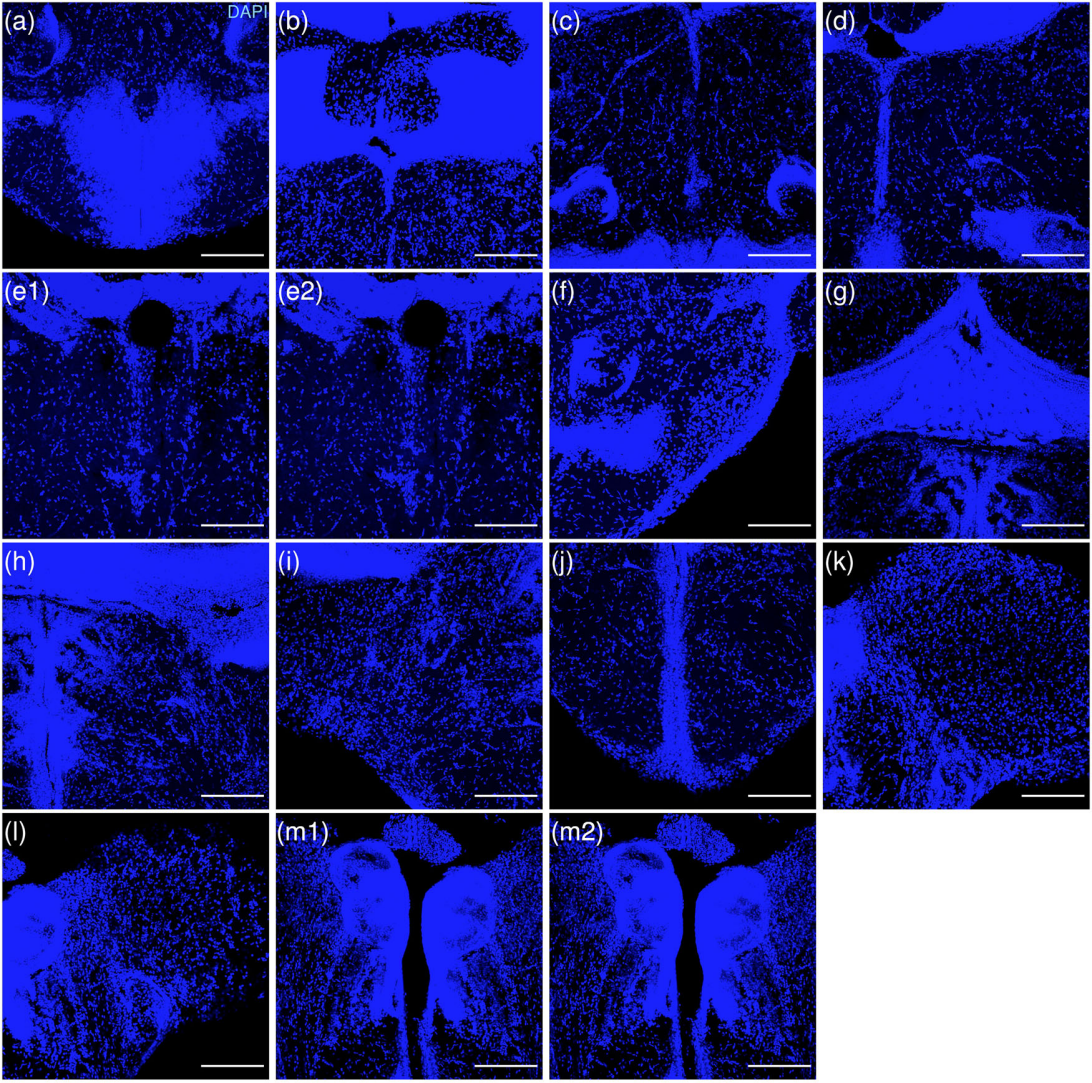
DAPI staining corresponding to Figure 23.

The level of sections is indicated in Figure 21a. Figure 23 shows serial sections arranged in caudorostral order from the IPN to the telencephalon (see Figure 24 for counterstaining with the corresponding panel number of Figure 23).

###### 3.2.3.2.1 Mesencephalon and Diencephalon

The periventricular hypothalamus is split into two subregions: the Hc and the Hd. The Hd flanks the ventromedially located Hc on both sides (Figure 23a). Hd somata express GFP weakly and congregate in the dorsal aspect of the Hd. Somata in the Hc and Hd appear to be morphologically different, with Hc somata taking on a round shape and those in the Hd being irregularly shaped.

The medial division of valvula cerebelli (Vam) contains somata that give rise to varicosity‐bearing plexuses scattered across the structure (Figure 23b). Posterior to the Vam, the lateral SZMT has a layer of somata arranged horizontally along the ventral TeV border close to the midline (Figure 23b). These cell bodies emit long, fine processes that cascade ventrally. Dorsolateral to the lateral SZMT, the EW contains a large neuron that emits a thick, ventrally coursing neurite (Figure 23c).

The nucleus of MLF (NMLF) is a relatively large region at the midline, located directly underneath the TeV (Figure 23d). The majority of neurons in the NMLF are organized along or close to the midline column, with a few scattered within the region.

In the hindbrain, CSF‐cNLs line the perimeter of the RV and emit their characteristically long neurites in virtually all directions (Figure 23e). Although CSF‐cNL neurites benefit from signal amplification with anti‐GFP, CSF‐cNL cell bodies are intensely positive for GFP (Figure 23e, 2).

The preglomerular nucleus (PG) is composed of a dense, arched neuropil that abuts the TLa. Some neurites exit the PG neuropil and travel toward the Hd, which contains a moderate cluster of somata (Figure 23f). Some of the Hd somata emit horizontally oriented neurites that extend out toward the lateral margin of the brain, whereas another cohort of neurites veers up toward the PG.

The TL is weakly innervated, containing a sparse plexus with a smattering of puncta spread throughout the region (Figure 23g). The ventrolateral corners of the TL are flanked by the mesencephalic nucleus of the trigeminal nerve (MNV) as a dense arrangement of somata (Figure 23h). Neurites from the somata appear to extend horizontally underneath the TL, crossing the midline. Ventral to the TL, the PPv sandwiches the DiV (Figure 23h) with its cell bodies lining the walls of the DiV at its dorsal aspect. The PPp at the ventral aspect of the brain contains several somata distributed along the midline (Figure 23j).

The pretectal nucleus (P) contains an intricately intertwined plexus that appears to be contiguous with the neuropil in the TeO (Figure 23i). The rostral TeO shows an extensively arborized plexus of neurites studded with varicosities that spreads over the bulk of the structure (Figure 23k,l).

The dHb is strongly labeled, containing GFP‐expressing somata in both hemispheres (Figure 23m, 2). Although the distribution of somata is bilateral, it is predominant in the medial subregion of the dHb (Figure 23m). Somata in the dHb emit ventrally projecting axons that exit the Hb to form the FR. Dorsal to the dHb, somata in the epiphysis (E) are brightly labeled for GFP (Figure 23m, 2).

#### 3.2.4 The Similarity of Cerebrospinal Fluid‐Contacting Neurons (CSF‐cNs) and CSF‐cNLs

**FIGURE 25:**
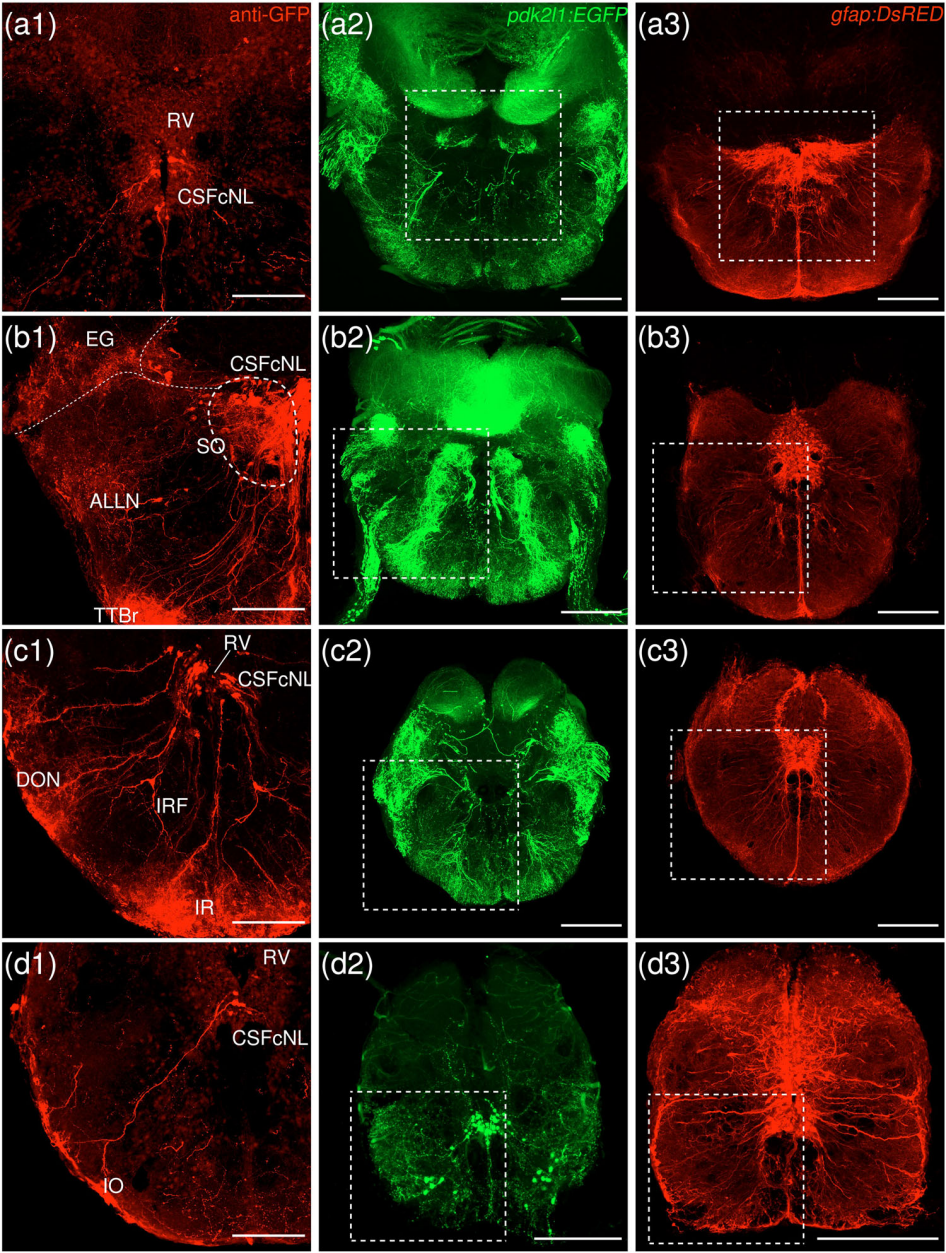
Comparison of CSF‐cNLs, CSF‐cNs, and radial glia. (a–d) Coronal sections of the adult zebrafish brain sections showing labeled CSF‐cNLs, CSF‐cNs, and radial glia. Pictures are confocal optical projections of serial cryotome‐cut coronal sections. (a1–d1) Adapted from Figures 2 , 3, 4, 5, 6, 7, 8, 9, 10, 11, 12. Scale bars = 150 µm. (a2–d2) *Tg(pkd2l1:GAL4; UAS:GFP)* immunostained for GFP (green). Scale bars = 250 µm. (a3–d3) *Tg(gfap:dTomato)* immunostained for dTomato (red). Scale bars = 250 µm. All panels are oriented with dorsal upward. For abbreviations, see the list.

To further confirm the similarity between CSF‐cNs and CSF‐cNLs, we used the transgenic line *Tg(pkd2l1:GAL4; UAS:GFP)*, which has been reported to label CSF‐cNs in larval zebrafish (Djenoune et al. 2017). Through the comparison of the staining pattern in the approximate coronal planes, we observed that both CSF‐cNs and CSF‐cNLs exhibit fibers of similar morphology that extend to the pial surface. In the caudal rhombencephalon, the fibers emitted by the CSF‐cNs and CSF‐cNLs specifically extend to the ventrolateral horn (Figure 25c1,c2,d1,d2).

On the contrary, the radial glial cells labeled in *Tg(gfap:dTomato)* fish (van Raamsdonk et al. 1984; Shimizu et al. 2015; Johnson et al. 2016) are widely distributed, covering most regions of the caudal rhombencephalon (Figure 25a3,b3,c3,d3); this distinguishes the CSF‐cNs from the radial glial cells. Likewise, in the rostral rhombencephalon, both CSF‐cNs and CSF‐cNLs exhibit the fibers that are mostly distributed around the midline of the ventral brain (Figure 25c1,c2,d1,d2), allowing discernment of radial glia cells (Figure 25c3,d3).

## 4 Discussion

**FIGURE 26:**
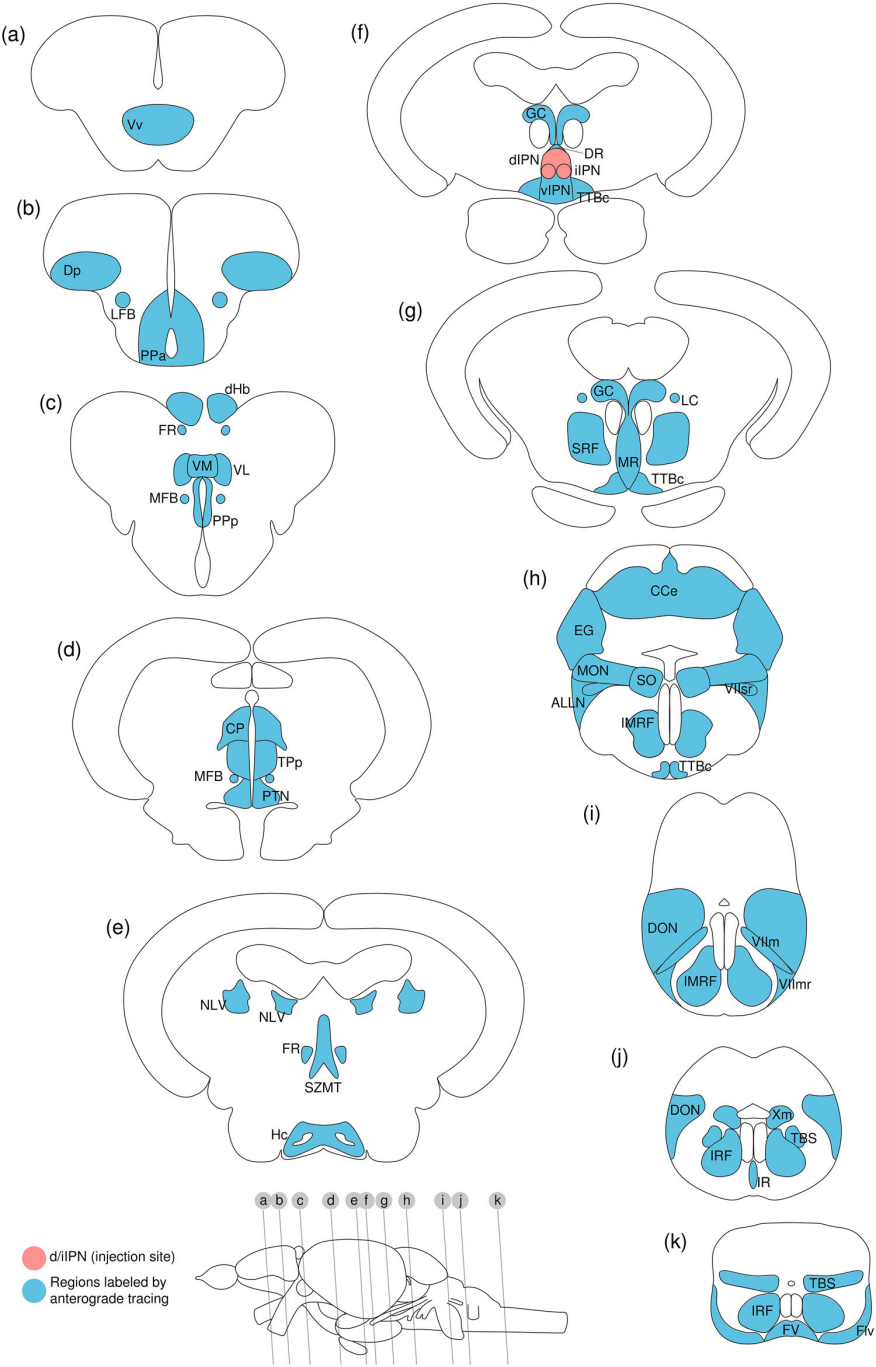
Schematic diagram of coronal sections of the adult zebrafish brain summarizing the regions labeled following injection of VSV‐mCherry as a trans‐synaptic anterograde tracer in the d/iIPN. (a–e) Regions rostral to the IPN. (f) d/iIPN (injection site). (g–k) Regions caudal to the IPN. The level of sections is indicated at bottom left. Diagrams are arranged in rostrocaudal order. All panels are oriented with dorsal upward. For abbreviations, see list.

**FIGURE 27:**
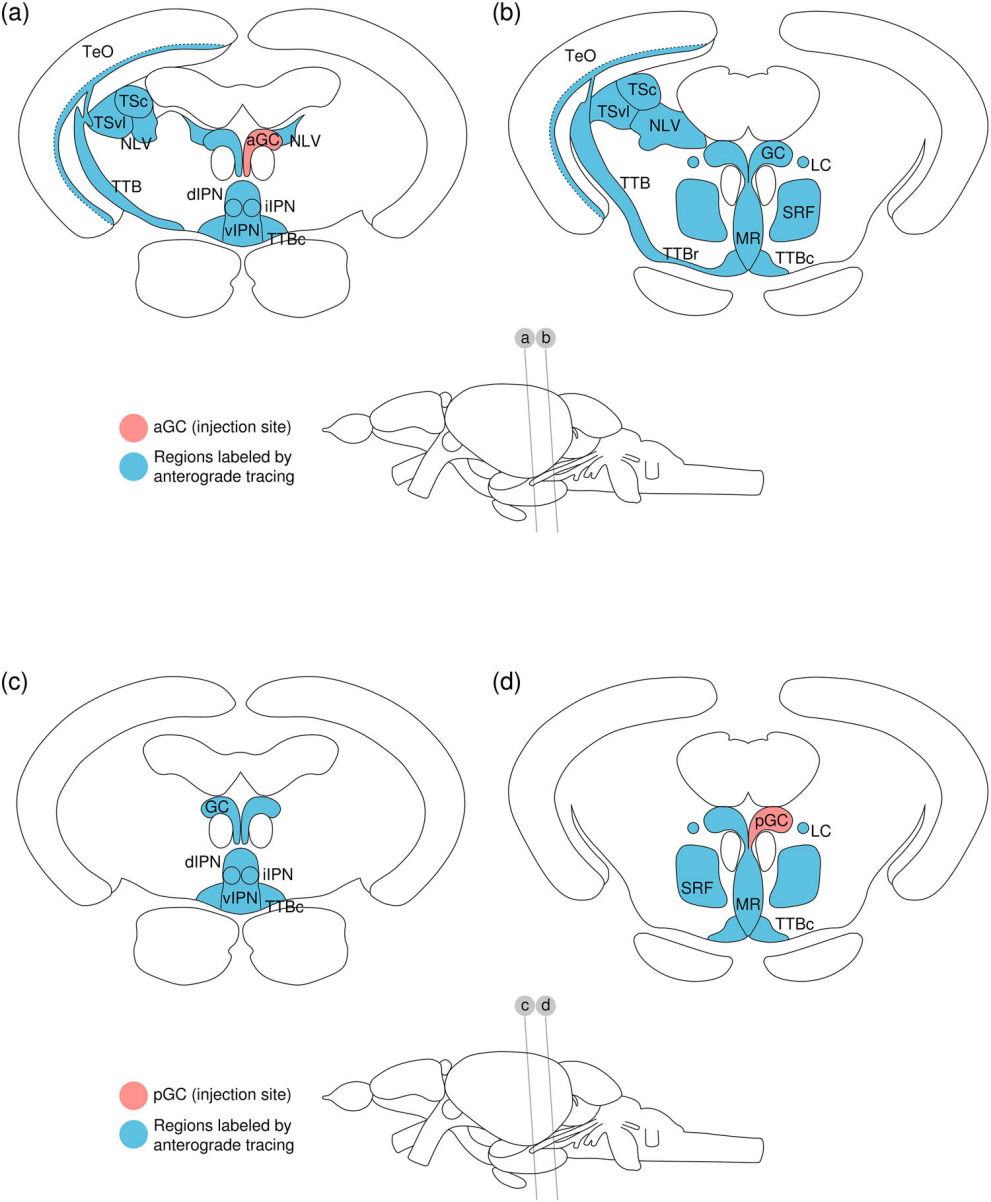
Schematic diagram of coronal sections of the adult zebrafish brain summarizing the regions labeled following injection of VSV‐mCherry as a trans‐synaptic anterograde tracer in the GC. (a) aGC (injection site). (b) Regions caudal to the aGC. (c) Regions rostral to the pGC. (d) pGC (injection site). The level of sections is indicated underneath (a, b) and (c, d). Diagrams are arranged in rostrocaudal order. All panels are oriented with dorsal upward. For abbreviations, see list.

**FIGURE 28:**
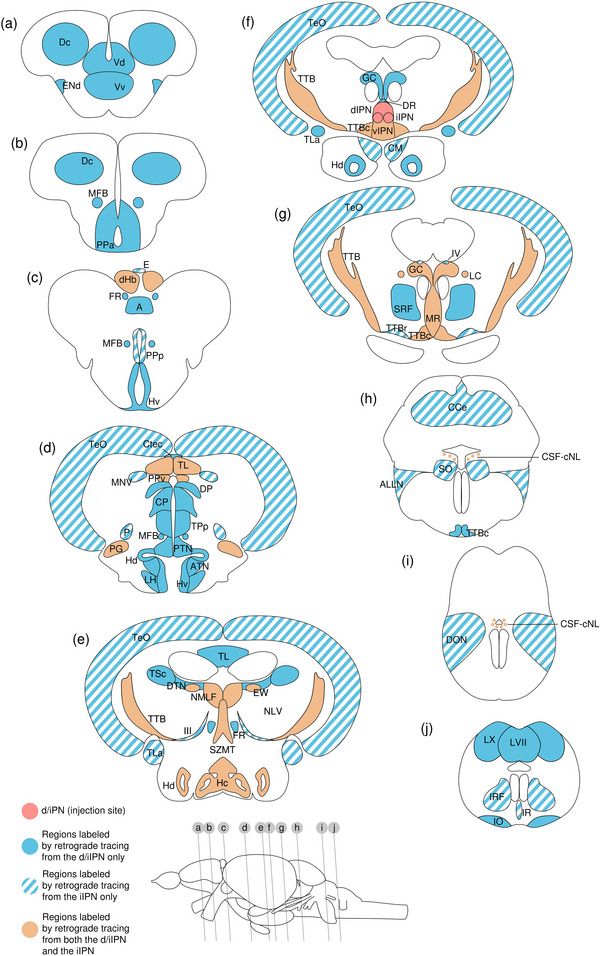
Schematic diagram of coronal sections of the adult zebrafish brain summarizing the regions labeled following injection of VSV(RABV‐G)‐GFP as a trans‐synaptic retrograde tracer in the d/iIPN or the iIPN. (a–e) Regions rostral to the IPN. (f) d/iIPN (injection site). (g–j) Regions caudal to the IPN. The level of sections is indicated at bottom left. Diagrams are arranged in rostrocaudal order. All panels are oriented with dorsal upward. For abbreviations, see list. Solid blue colors represent the labeled regions by the d/iIPN injection, and striped blue color represents the labeled regions exclusively traced by the iIPN injection, whereas the yellow color represents the labeled regions traced both from d/iIPN and iIPN injection.

**FIGURE 29:**
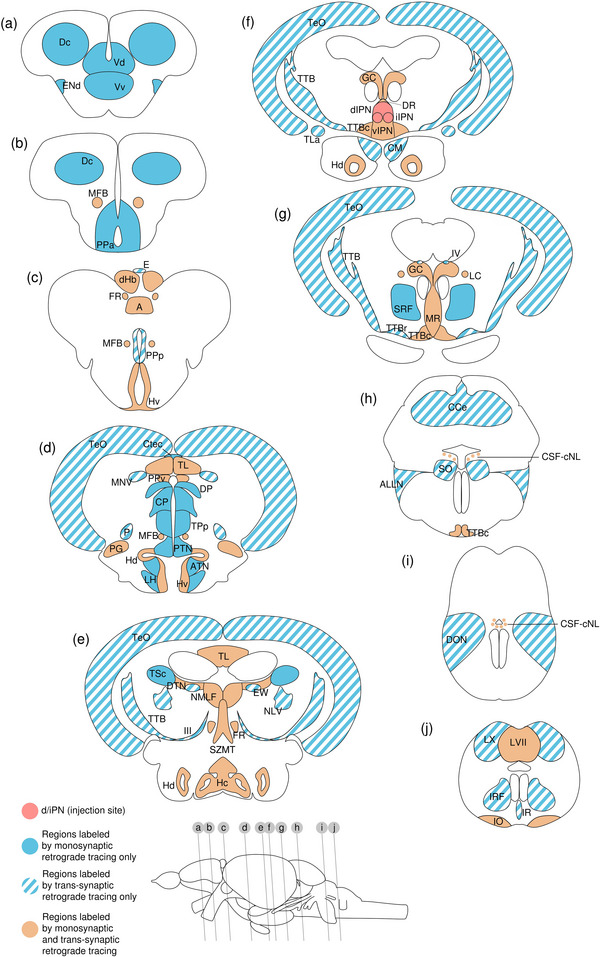
Schematic diagram of coronal sections of the adult zebrafish brain summarizing the regions labeled following injection of HSV‐GFP as a monosynaptic retrograde tracer and VSV(RABV‐G)‐GFP as a trans‐synaptic retrograde tracer in the d/iIPN (including d/iIPN and iIPN injections). (a–e) Regions rostral to the IPN. (f) d/iIPN (injection site). (g–j) Regions caudal to the IPN. The level of sections is indicated at bottom left. Diagrams are arranged in rostrocaudal order. All panels are oriented with dorsal upward. For abbreviations, see list. Solid blue colors represents the labeled regions exclusively by the monosynaptic retrograde viral tracing (HSV), and striped blue color represents the labeled regions exclusively traced by the trans‐synaptic retrograde viral tracing (VSV(RABV‐G)) (d/iIPN and iIPN injection), whereas the yellow color represents the labeled regions traced both from monosynaptic and trans‐synaptic viral tracer.

Although the functional studies have revealed the importance of d/iIPN in regulating emotional behavior, coping with social conflicts, engaging in navigation, and making decisions (Agetsuma et al. 2010; Lee et al. 2010; Duboué et al. 2017; Dragomir et al. 2019; Cherng et al. 2020; Nakajo et al. 2020; Palumbo et al. 2020; Okamoto et al. 2021; Kinoshita and Okamoto 2023; Petrucco et al. 2023; Handa et al. 2025; Matsumata et al. 2025), the comprehensive connection map is unknown. We mapped the connectivity from d/iIPN using anterograde and retrograde trans‐synaptic viral tracing, retrograde mono‐synaptic viral tracing, and DiI to lay the anatomical foundation for the IPN's link with various brain regions (Figures 26, 27, 28, 29). Here, we discuss each connection with some functional conjectures.

### 4.1 Technical Considerations

Our results are based on injecting anterograde viral tracers into the d/iIPN of three fish and into the GC of two (Table 3). In retrograde tracing, we injected monosynaptic HSV into the d/iIPN of one fish, and the trans‐synaptic VSV into the d/iIPN of one fish and the iIPN of two fish (Table 3).

**TABLE 3:** Conditions of Virus Microinjection **Anterograde trans‐synaptic tracing using VSV mCherry**.

d/iIPN	A–P (µm)	Depth (µm)	Head length/width (mm)	Body length/width (mm)	The volume of injected virus solution (nL)	Tracing duration (days)
210306s3	10	1150	51.0/27.0	261/27	100	5
210603s5	10	1150	51.5/28.0	274/31	100	5

iIPN						
210306s4	10	1100	53.5/29.0	258/30	100	5

GC						
210306s2	10	1150	52.0/30.0	263/37	100	5
210306s6	10	1200	49.0/30.5	257/35	100	5

Retrograde mono‐synaptic tracing using HSV‐GFP						
d/iIPN	A–P (µm)	Depth (µm)	Head length/width (mm)	Body length/width (mm)	The volume of injected virus solution (nL)	Tracing duration (days)
211105s3	9	1400	59.0/30.0	287/32	50	6

Retrograde trans‐synaptic tracing using VSV(RABV‐G)‐GFP						
d/iIPN	A–P (µm)	Depth (µm)	Head length/width (mm)	Body length/width (mm)	The volume of injected virus solution (nL)	Tracing duration (days)
220128s6	9	1450	56.0/46.0	295/37	100	5

iIPN						
220121s4	9	1400	51.0/33.0	298/33	100	5
220121s1	9	1450	48.0/35.0	299/37	50	5

There may be variances in each stereotaxic microinjection due to individual anatomical differences, even though the injection sites were targeted with reference to the designated coordinates based on the anatomical landmarks on the skull. The relatively small sample size is due to the difficulty of in vivo microinjections and of striking a balance between the viability of the fish and the delivery of infectious particles deep into the brain.

Notably, due to the trans‐synaptic feature of viral tracers, the direction of propagation may not be easily discernible in the event of reciprocal connections. The signal intensity may be diluted as a function of multiplicity in trans‐synaptic connections. Additionally, labeling in highly reciprocal regions experiences a boost due to additive production in trans‐synaptic viral tracing. The term “neurite” is used to delineate the undistinguished projection of axons and dendrites, whereas we use the term “fiber” to refer to the axonal projection. Although we acknowledge the importance of identifying them as axons or dendrites with respect to their directionalities, the detailed information of these circuits should be investigated through monosynaptic tracing in future studies.

### 4.2 The d/iIPN Reciprocally Connects With the dHb and the GC

The dHb is directly upstream of the IPN (Aizawa et al. 2005; Gamse et al. 2005; Agetsuma et al. 2010; Amo et al. 2010; Chou et al. 2016; Kinoshita and Okamoto 2023). Following the injection of retrograde viral tracers in the IPN, dHb somata exhibit clear fluorescent signals (Figure 19g), showcasing the precision and effectiveness of in vivo viral tracing in this study. In addition to the retrograde labeling seen in the dHb, fibrous signal in the dHb and FR following anterograde tracing is observed (Figure 5g), implying reciprocal connections between the dHb and IPN.

Although the backward connections from the d/iIPN to the dHb have not been explicitly mentioned in previous studies, we noticed that somata were unexpectedly labeled in the dIPN following the placement of DiI on the dHb (see figure 1e in Aizawa et al. 2005). The left dHb is reported to receive indirect visual input, whereas the right Hb receives direct olfactory input, sending exteroceptive information to the IPN (Miyasaka et al. 2009; Dreosti et al. 2014; Zhang et al. 2017; Fore et al. 2018).

The GC, corresponding to the mammalian PAG, is a conserved nucleus across vertebrates (Olson et al. 2017). Its longitudinally extended structure lies ventrally beneath the RV and extends rostrally into the mesencephalon (Wullimann et al. 1996). The results from our anterograde and retrograde tracings show that the entirety of the GC is covered with robust signals (Figures 2b–e and 7c,d), and due to the trans‐synaptic tracing method, the GC somata are labeled. These reciprocal connections demonstrated in our results are consistent with previous findings (Agetsuma et al. 2010).

### 4.3 The d/iIPN Connects With the Neural Modulator Systems Related to Emotion and State Regulation

Our results confirm that the iIPN is anterogradely connected with the MR (Figure 2d,e) (Agetsuma et al. 2010; Chou et al. 2016; Kinoshita and Okamoto 2023). We also show that the GC is reciprocally connected with the MR (Figures 9i,13c, and 17c) and that the MR is robustly labeled following monosynaptic retrograde tracing (HSV) from the d/iIPN (Figure 13c). The MR exhibits a prominent ascending projection to the pallium in adult zebrafish (Shibayama et al. 2024). Additionally, the DR is labeled in all d/iIPN‐injected samples (Figures 7b,c and 13b). In mammals, the serotonergic system has been suggested to be a major regulator of emotions (Hensler 2006; Paul and Lowry 2013) and has been recently revealed to play a critical role in controlling states of alertness, valance learning, and motor adaptation (Stephenson‐Jones et al. 2012; Amo et al. 2014; Kawashima et al. 2016; Lovett‐Barron et al. 2017; Yang et al. 2021). Our results support the findings that the d/iIPN may control the serotonergic system and functionally regulate distinct aversive responses (Agetsuma et al. 2010; Paul and Lowry 2013; Commons 2016).

The LC (Figures 2d and 9d), the SRF, (Figures 2d,e, and 9d), and the IRF (Figures 2k and 21j) exhibit strong labeling in either anterograde or retrograde tracing. Specifically, LC and SRF neurons are retrogradely labeled by the monosynaptic retrograde tracer (HSV), indicating that LC neurons project directly to the d/iIPN (Figure 13d). LC and SRF neurons are well characterized through their neurotransmitters; most noradrenergic neurons are located in the LC (Ma 1994a), whereas the cholinergic neurons are primarily situated in the SRF (Mueller et al. 2004). A recent study in larval zebrafish reported that in futile attempts to disrupt expected visual flow, noradrenergic neurons in the LC progressively activate brainstem radial astrocytes, accumulating evidence that current actions are ineffective and consequently drive changes in behavioral states (Mu et al. 2019). The detailed observations obtained through immunohistochemistry suggest the possibility that the LC innervates the IPN and MR (Ma 1994b). The IRF is labeled only when anterograde tracing is initiated from the d/iIPN and not from the GC (Figure 2k).

We report here that in anterograde viral tracing, some of the PTN somata (Figure 5d), as well as neurites en passant in the periventricular nucleus of the posterior tuberculum (TPp) (Figure 5e), are positively labeled. In comparison, only TPp somata are labeled in retrograde tracing (Figure 15i). The posterior tuberculum is composed of the TPp and PTN (Wullimann et al. 1996) and includes neurons immunoreactive to tyrosine hydroxylase (TH) antibodies; this suggests that the posterior tuberculum is the primary nucleus of the dopaminergic system (Rink and Wullimann 2004). The positively labeled PTN neurons are morphologically similar to the small round cells observed in anterograde tracing and the pear‐like cells in the TPp (Figure 5e), which are two of the three types of TH neurons reported previously (Rink and Wullimann 2004). The neurons in the PTN and in the TPp are also positive for orexin and neuropeptide Y immunoreactivity, which is sensitive in fasting zebrafish (Yokobori et al. 2012, 2011). In the thalamus, somata in the CP are conspicuously labeled in anterograde tracing (Figure 5e), but only sparsely labeled neurites are observed in the rest of the thalamic nucleus, including the ventrolateral (VL) and VM (Figure 5f). Other thalamic nuclei, such as the dorsal posterior thalamic nucleus (DP) and the A, are positively labeled by retrograde tracing (Figure 15h,i). Ventral to the TPp, the Hc is strongly labeled in both anterograde and retrograde tracings (Figures 5b and 15b,c). Prey capture and predatory behavior in zebrafish are related to its feeding state, which is under the control of the hypothalamic–pituitary–interrenal axis (Filosa et al. 2016). Diverse threats could activate the various neuropeptidergic neurons in the hypothalamus that initiate defensive behaviors by relaying signals to descending motor neurons in larval zebrafish (Lovett‐Barron et al. 2020).

### 4.4 The d/iIPN Connects With the Modules Related to Motion Detection

In anterograde tracing, through the subtraction of the regions traced from the iIPN, we show that the dIPN neurons are connected with vestibular nuclei (Figures 2f,j and 21i,j) such as the MON, the DON, and the (SO). In addition to vestibular signals, the ALLN projection area, which is the entry point of anterior lateral line inputs in the central brain (Coombs et al. 1989), shows conspicuous signals that indicate its connection with the dIPN (Figures 2f,4, and 21i).

### 4.5 The d/iIPN May Receive Inputs From the Proprioception and Interoceptive Circuits

Through retrograde tracing, we found labeled neurons in the vagal lobe (LX) (indirect connection) and in the facial lobe (LVII) (direct connection) (Figures 13g and 17g); these nuclei mayreceive somatosensory signals such as gustatory sensation, proprioception, and nociception (Stefan Nilsson and Susanne Holmgren 1984). Intriguingly, CSF‐cNLs embedded in the ependyma of the RV exhibit robust signal in all retrograde tracing samples (Figures 13h, 17f, 21h–j, 23e, and 25). Their characteristic localization at the ventricular walls and fine, long‐projecting neurites resemble those of the CSF‐cNs involved in the proprioception of locomotion and posture (Vígh et al. 2004; Wyart et al. 2009; Orts‐Del'Immagine et al. 2014; Nakamura et al. 2023). Based on the comparison of the CSF‐cNs in the transgenic line (*Tg(pkd2l1:GAL4; UAS:GFP)*) to the CSF‐cNLs identified in this study, it seems likely that they share a similar morphology and anatomical location (Figure 25). Recent studies in larval zebrafish have shown that CSF‐cNs project to the hindbrain motor column and provide feedback control in locomotor speed (Böhm et al. 2016; Hubbard et al. 2016; Wu et al. 2021). Similarly, in rodents, CSF‐cNs control locomotion (Nakamura et al. 2023) and skilled movement (Gerstmann et al. 2022). Our results implicate that the d/iIPN could be connected with these proprioception circuits.

### 4.6 The d/iIPN Is Reciprocally Connected With Motor Control Circuits

We have identified the motor pathways labeled by anterograde and retrograde tracings from the d/iIPN, but no motor nuclei have been concurrently labeled by both tracing directions. The TBS, which is part of the motor pathway, is anterogradely labeled (Figure 2l). In mammals, the TBS originates from the reticulospinal neurons (RSN) (Jankowska 2008) and may serve as the descending motor output in controlling the phrenic motor system, autonomic function, and motor control (Holstege 1996; Hardy et al. 1998; Leblond et al. 2000; Jankowska 2008; Huma et al. 2014; Ghali 2017). Our retrograde tracing also labeled neurons in the nucleus of the medial longitudinal fasciculus (nMLF), a small group of the RSN (Figure 15e). In zebrafish larvae, nMLF activity is correlated with the swimming bout that responds to visual stimuli, which directly alters tail position in causality manipulation (Thiele et al. 2014). Evidence indicates that the nMLF may regulate posture and balance through the control of the hypaxial muscles (Thiele et al. 2014; Sugioka et al. 2023). The ablation of neurons in the nMLF has been found to abolish prey capturing behaviors (Gahtan et al. 2002, Gahtan et al. 2005). The vagal nerve nucleus (Xm) (Figure 2k), which innervates the post‐trematic nerve branch and controls the pharyngeal muscle, is suggested to provide motor command (Nilsson 1984; Nilsson and Holmgren 1984; Crucke et al. 2015). Additionally, the Flv (Figure 2l) acts as the exit of rhombencephalic motor signals and carries motor command from the corticospinal tract and the rubrospinal tract in mammals (Felten et al. 2021).

The VIIm and VIImr, both of which are anterogradely labeled (Figure 2j), may control the movement of the jaw and gill covers (Luiten 1976; Gorlick 1989; Tanaka et al. 2007). The motor modalities controlling eye movements, such as the oculomotor nerve (III) and the trochlear nerve (IV), are labeled in retrograde tracing from the d/iIPN (Figure 21c,e). The IO, which has been found to encode visual‐based self‐position (Yang et al. 2021), shows positive signals in retrograde tracing (Figure 13h). Together, these connected motor modalities imply that the d/iIPN may be involved in regulating motor adaptation.

Aside from motor‐related modalities situated in the regions caudal to the d/iIPN, we found labeled neurites en passant in the preoptic area (PP; anterior [PPa] and posterior [PPp]) in anterograde tracing (Figure 5i) and labeled somata in the same region in retrograde tracing (Figure 15q). A recent study has shown that the PP and Hb are involved in homeostatic navigation in the context of temperature gradients (Palieri et al. 2024), suggesting that the PP and Hb jointly support navigation. The PP may play a role in nondirectional reorientation, whereas the Hb is involved in directional movement (Palieri et al. 2024).

The TL and EW nucleus are positively labeled in retrograde tracing (Figures 15h, 19c, and 23g). Nonviral anatomical tracing in zebrafish, carp, and squirrelfish shows that TL projections terminate at the marginal layer of the TeO (stratum marginals) (Ito and Kishida 1978; Ito et al. 2003; Xue et al. 2003; Folgueira et al. 2020). It was reported that the TL also innervates the lateral thalamic nucleus (Folgueira et al. 2020). Functionally, a study has found that TL neurons are connected to the oculomotor nucleus (NIII) (Wullimann and Roth 1994). The ventrolateral subregion of the NIII is activated with saccadic eye movement and has been postulated to be involved in visual prediction (Northmore et al. 1983; Northmore 2017; Yamamoto and Hagio 2021) and has been proposed to function together with lateral line input for goal‐directed movement (Meek 1992). On the other hand, the EW nucleus, as part of the NIII region, is involved in the autonomic regulation of the eye, which includes pupillary constriction in mammals (Kozicz et al. 2011) and visual accommodation in teleost (Wathey 1988; Somiya et al. 1992).

### 4.7 Higher Order Motor Regulatory Substrates Are Situated Downstream of the d/iIPN

The NLV and EG in the corpus cerebelli (CCe) of the cerebellum are labeled in anterograde tracing from the d/iIPN (Figures 2f, 5a, and 9b). In teleosts, the NLV is a choline acetyltransferase‐positive region (Mueller et al. 2004) with the efferent terminals in the cerebellum (Dohaku et al. 2019), and the NLV has been reported to receive axonal innervation from the IR (Lillesaar et al. 2009). Retrograde labeling shows that the NLV and DTN are connected with the d/iIPN (Figures 17d and 19a), which is consistent with anatomical evidence in rats showing innervation of the IPN by the DTN and potentially also by cholinergic neurons (Hemmendinger and Moore 1984; Liu et al. 1984). DTN neurons are known to exhibit head directional responses and are hypothesized to be the central generator of head directional signals in rats (Sharp et al. 2001; Clark and Taube 2012); this function is potentially conserved in zebrafish (Petrucco et al. 2023). The DTN receives neural signals from the brain regions that sense the velocity of the body and eye movements, such as the nucleus prepositus hypoglossi (NPH) and the medial vestibular nucleus (MVe) (Mehlman et al. 2021). These results suggest that the d/iIPN could highly interact with the cerebellar system and head directional circuits, which are anatomically conserved in mammals.

### 4.8 Integrated Sensory Inputs May Be Conveyed to the TeO and the TS via the d/iIPN–GC Tract

Results from anterograde and retrograde tracings show that the d/iIPN is linked with networks that are functionally related to multimodal sensory processing. These nuclei, which include the TeO (homologous to the mammalian superior colliculus) and the TS (homologous to the mammalian inferior colliculus), have been reported to be involved in visual processing, vestibulovisual sensory integration, and visuomotor transformation (Thompson et al. 2016; Isa et al. 2021; Baier and Scott 2024).

The TeO receives topographic visual signals from the retina (Isa et al. 2021). The descending output from the TeO runs through two primary tracks, TTBc and TTBr, and further diverges to multiple regions in the brain stem (Dean et al. 1986, Dean et al. 1989; Sato et al. 2007). These two pathways together modulate orienting movements, such as approach and avoidance, which are, respectively, controlled by the crossed and uncrossed TTB pathways. Our results show that the entire TTB–TBS pathway is positively labeled in anterograde tracing (Figures 2d,e,g and 9e).

In addition to visual signals, the TeO has been shown to respond to other sensory information, such as water current in *Xenopus* (Zittlau et al. 1986) and auditory stimulus in larval zebrafish (Thompson et al. 2016; Isa et al. 2021; Baier and Scott 2024). Our observations (Figure 9f,g) are consistent with the findings that a small proportion of TS projection extends to the TeO (Fame et al. 2006; Sassa et al. 2007) and show linkage of neurites between the TS and TTB (Figure 9e). It is possible that the positive labeling originates from the cholinergic input of the SRF neurons as previously suggested (Mueller et al. 2004). The deep neuropil of the TeO also receives inhibitory input from the hypothalamus (Heap et al. 2018), suggesting that the multimodal integration of TeO signals plays a role in regulating behavioral responses.

Following our results from retrograde tracing from the d/iIPN using VSV and HSV, we reason that the TeO is indirectly connected to the d/iIPN (Figures 15k and 23k,l). In combination with observations from anterograde tracing, we postulate a hierarchical reciprocal connection between the TeO and dIPN (dIPN–aGC–*x*–TeO–TTBc–vIPN–d/iIPN loop circuits, where *x* denotes multiple connections within a hierarchy) (Figures 9e,f,k and 23k).

The TS is found to be positively labeled when retrogradely traced with the monosynaptic HSV from the d/iIPN (Figure 15e,g). When anterogradely traced with the trans‐synaptic VSV, the TS, together with other somatosensory‐related regions such as the ALLN projection region, MON, and DON, is also labeled (Figures 2f,i,k,j and 9e). These results suggest that the above nuclei communicate reciprocally with the d/iIPN through indirect connections.

### 4.9 Possible Roles of the Hb–IPN–GC Circuit in Visuomotor Transformation and in Stress Resilience by Allostasis

We demonstrate that the d/iIPN is connected with distinct brain modalities that potentially regulate motor control, sensory integration, and emotional state through the influence of neuromodulators such as acetylcholine (in the SRF), noradrenaline (in the LC), dopamine (in the TPp), and serotonin (in the raphe) (Figures 2 , 3, and 7d). We show that the d/iIPN may be reciprocally connected with the GC, the homolog of the mammalian PAG. The PAG is well known for controlling defensive behavior in mammals (Lefler et al. 2020) and is deeply involved in fear learning in rats (Johansen et al. 2010). It has recently been proposed that the PAG plays an extended role in calculating prediction errors of aversive stimuli (Ozawa et al. 2017), further linking the function of the d/iIPN to the process of learning and regulating emotion.

Based on the homology between the zebrafish GC and the mammalian PAG, it is possible that the GC or the IPN, which is reciprocally connected with the GC, may also function to calculate prediction error. Information on the interoceptive and exteroceptive status of the body may be conveyed to the IPN and GC through their reciprocal connections with the motor and sensory systems. This is then compared to the predictive codes possibly generated by the Hb–IPN circuit in either the GC or the IPN. The calculated prediction error could then be projected through the TTB to the TeO in order to increase the efficiency of visuomotor transformation in various behaviors such as in targeting opponents in social conflict or in prey capture. This error could also regulate the activities of neuromodulator systems, such as the serotoninergic neurons in the MR and DR, the noradrenergic neurons in the LC, and the dopaminergic neurons in the posterior tuberculum.

In dyadic fights, the experience of winning could synergistically induce a weight shift in the selection of information from other‐directed to self‐centered, concomitantly with the state of the brain becoming more egocentric. This readies their internal states, such as body posture and resilience against stress, in attacking their opponent during social conflict. Animals acquire resilience against stress by adapting to changes in their external environments through predictively readjusting their internal status; this capacity is termed “allostasis” (Schulkin and Sterling 2019; Santamaría‐García et al. 2024). Considering the reciprocal connections of the IPN and GC with multiple interoceptive systems, such as the octavolateralis system, the proprioceptive system, and the vagal autonomic nervous system, and the potentiation of the Hb–IPN–GC circuit in increasing the resiliency to social conflict stress in fish, the Hb–IPN–GC circuit may play a key role in modulating anticipatory biological reactions to upcoming external challenges, that is, allostasis. In mammals, this circuit, in concert with other higher brain centers such as the insula in the cortex, may act as a part of the hierarchical neural networks that regulate stress resilience (Nestler and Russo 2024; Santamaría‐García et al. 2024).

## Author Contributions

A.K.W., B.‐W.C., and H.O. conceived the study, designed the experiments, and wrote the manuscript. A.K.W. and B.‐W.C. carried out all of the experiments and data analysis. H.K. constructed and produced the viruses. C.W. shared her transgenic lines. H.O. supervised the research project. All of the authors read and approved the manuscript.

## Funding

H.O. was supported by the MEXT KAKENHI Grant‐in‐Aid (21H04814, 22H05520, and 23H04976) and partially supported by the Collaboration Fund from Kao Corporation. C.W. was supported by the European Research Council Consolidator Grant Exploratome #101002870.

## Conflicts of Interest

The authors declare no conflicts of interest.

## Peer Review

The peer review history for this article is available at https://doi.org/10.1002/cne.70105.

## Data Availability

The data supporting this study's findings are available from the corresponding author upon reasonable request.
